# Effectiveness of different de-implementation strategies in primary care: systematic review and meta-analysis

**DOI:** 10.1136/bmjmed-2025-001343

**Published:** 2025-09-09

**Authors:** Aleksi Raudasoja, Sameer Parpia, Jussi M J Mustonen, Robin Vernooij, Petra Falkenbach, Yoshitaka Aoki, Anton Barchuk, Marco H Blanker, Rufus Cartwright, Kathryn Crowder, Herney Andres Garcia-Perdomo, Rachel Gutschon, Alex L E Halme, Tuomas P Kilpeläinen, Ilari Kuitunen, Tiina Lamberg, Eddy Lang, Jenifer Matos, Olli P O Nevalainen, Niko K Nordlund, Negar Pourjamal, Eero Raittio, Patrick O Richard, Philippe D Violette, Jorma T Komulainen, Raija Sipilä, Kari A O Tikkinen

**Affiliations:** 1 Finnish Medical Society Duodecim, Helsinki, Finland; 2 Faculty of Medicine, University of Helsinki, Helsinki, Finland; 3 Department of Health Research Methods, Evidence, and Impact, McMaster University, Hamilton, Ontario, Canada; 4 Department of Oncology, McMaster University, Hamilton, Ontario, Canada; 5 Mehiläinen Oy, Helsinki, Finland; 6 Department of Nephrology and Hypertension, University Medical Centre Utrecht, Utrecht, Netherlands; 7 Julius Centre for Health Sciences and Primary Care, University Medical Centre Utrecht, Utrecht, Netherlands; 8 Finnish Coordinating Center for Health Technology Assessment, Oulu University Hospital and University of Oulu, Oulu, Finland; 9 Department of Urology, Nagoya City University Mirai Kousei Hospital, Nagoya, Japan; 10 Department of Medical Informatics, Erasmus Medical Center, Rotterdam, Netherlands; 11 Department of Primary and Long-Term Care, University Medical Centre Groningen, Groningen, Netherlands; 12 Institute of Reproductive and Developmental Biology, Imperial College London, London, UK; 13 Department of Urogynaecology, Chelsea and Westminster NHS Foundation Trust, London, UK; 14 Department of Emergency Medicine, University of Calgary Cumming School of Medicine, Calgary, Alberta, Canada; 15 Department of Surgery, Universidad del Valle, Cali, Colombia; 16 Faculty of Health and Medical Sciences, University of Western Australia, Perth, Western Australia, Australia; 17 Department of Urology, University of Helsinki and Helsinki University Hospital, Helsinki, Finland; 18 Institute of Clinical Medicine, University of Eastern Finland, Kuopio, Finland; 19 Ministry of Public Health of the Dominican Republic, Santo Domingo, Dominican Republic; 20 Hatanpää Health Centre, Wellbeing Services County of Pirkanmaa, Tampere, Finland; 21 Department of Dentistry and Oral Health, Aarhus University, Aarhus, Denmark; 22 Institute of Dentistry, University of Eastern Finland, Kuopio, Finland; 23 Division of Urology, Department of Surgery, Sherbrooke University Hospital, Sherbrooke, Quebec, Canada; 24 Department of Surgery, McMaster University, Hamilton, Ontario, Canada; 25 Department of Surgery, Woodstock Hospital, Woodstock, Ontario, Canada; 26 Department of Surgery, Päijät-Häme Central Hospital, Lahti, Finland

**Keywords:** Education, medical, Clinical audit, Clinical trial, Health policy, Primary health care

## Abstract

**Objective:**

To evaluate the effectiveness of various de-implementation interventions in primary care, targeting care (treatments or tests) that provides no or limited value for patients (low value care).

**Design:**

Systematic review and meta-analysis.

**Data sources:**

Medline and Scopus databases, from inception to 10 July 2024.

**Eligibility criteria for selecting studies:**

Randomised trials comparing de-implementation interventions with placebo or sham intervention, no intervention, or other de-implementation intervention strategies in primary care. Eligible trials provided information on the use of low value care, total volume of care, appropriate care, and health outcomes.

**Data extraction and synthesis:**

Titles, abstracts, and full texts were screened, data were extracted, and risk of bias was assessed independently and in duplicate. Random effects meta-analyses were conducted, and the certainty of evidence was assessed with the Grading of Recommendations Assessment, Development, and Evaluation (GRADE) approach.

**Results:**

13 008 abstracts were screened and 140 were eligible for inclusion in the study. Median follow-up was 287 days (interquartile range 180-365). In 75 (54%) trials the aim was to reduce the use of antibiotics, in 42 (30%) to reduce other drug treatments, in 17 (12%) to reduce imaging, and in 15 (11%) to reduce laboratory testing. The certainty of the evidence was moderate that provider education combined with audit and feedback reduced the use of targeted low value care (odds ratio 0.73, 95% confidence interval (95% CI) 0.63 to 0.84). Provider education (0.86, 95% CI 0.72 to 1.03), audit and feedback (0.82, 0.67 to 1.00), and patient education (0.70, 0.30 to 1.66), and a combination of these strategies (point estimates for odds ratios ranging from 0.57 to 0.64) may reduce the use of targeted low value care (low certainty of evidence for all).

**Conclusions:**

The results suggested with moderate certainty of evidence that provider education combined with audit and feedback reduced the use of targeted low value care. Individual strategies may slightly reduce the use of targeted low value care, but achieving a meaningful impact on low value care may require the use of multiple strategies. The results may be useful for patients, clinicians, policy makers, and guideline developers when deciding on future de-implementation strategies and research priorities.

**Systematic review registration:**

PROSPERO CRD42023411768.

**WHAT IS ALREADY KNOWN ON THIS TOPIC**

- Low value care is defined as care that provides little to no value for patients, may be harmful, and by wasting limited resources is a major problem in modern healthcare, including in primary care
- De-implementation strategies may help achieve higher quality healthcare by decreasing the use of low value care
- No comprehensive systematic review and meta-analysis exists on the effectiveness of de-implementation interventions

**WHAT THIS STUDY ADDS**

- This systematic review identified 140 randomised trials exploring de-implementation strategies in primary care
- Pooled moderate certainty of evidence indicated that provider education combined with audit and feedback reduced the relative risk of targeted low value care by about 23%
- Low certainty of evidence suggested that individual strategies (such as audit and feedback, or patient education) may slightly reduce the use of targeted low value care, but combining patient education with other strategies could reduce the relative risk by about 30-35%

**HOW THIS STUDY MIGHT AFFECT RESEARCH, PRACTICE, OR POLICY**

- Interventions combining provider education with audit and feedback could be prioritised, as this strategy had the highest certainty of evidence
- Patient education, especially as part of combined strategies, deserves greater attention in both de-implementation efforts and future research
- Future practice should focus on implementing reproducible, evidence based interventions and measuring their local impact
- A network meta-analysis could help future research explore which strategies have the greatest impact

## Introduction

 Low value care is defined as care that provides little or no benefit, potentially causes harm, incurs unnecessary costs to patients, or wastes healthcare resources.^1^ Low value care has been estimated to be highly prevalent in primary care; a study in the US estimated that about 75% of antibiotic prescriptions in primary care are inappropriate.^2^ In a systematic review and meta-analysis, overuse of diagnostic tests, such as endoscopies, urine cultures, and knee x ray images, was also found to be common in primary care.^3^

Healthcare costs are rising worldwide. To maintain the sustainability of current healthcare systems and reduce the risks of patient harm, reducing low value care is important. De-implementation refers to strategies for reducing low value care and can be divided into four categories: removing, replacing, reducing, or restricting the use of low value care.^4^

Our scoping review on de-implementation research in randomised controlled trials,^5^ published in 2022, identified 227 trials, 149 conducted in primary care. Although previous reviews and systematic reviews on de-implementation strategies exist,^6^,^8^ collectively these reports included only a minority of published randomized controlled trials and did not perform meta-analyses. We conducted a comprehensive systematic review and meta-analysis of the effectiveness of different de-implementation strategies compared with no intervention or other de-implementation strategies in reducing the use of low value care in primary care.

## Methods

We followed the Preferred Reporting Items for Systematic Reviews and Meta-Analyses (PRISMA) guidelines for this systematic review and meta-analysis.^9^

### Data sources and searches

In collaboration with an experienced information specialist (TL), we developed a comprehensive search strategy for our previous scoping review.^5^ For this systematic review, we updated the search based on the same strategy (online supplemental methods 1). We searched Medline and Scopus for individual and cluster randomised controlled trials of de-implementation interventions, without limits on language, from inception to 10 July 2024. We then identified systematic reviews, searched their reference lists, and added potential articles to the selection process. We also hand searched for study protocols and other study reports to ensure accurate categorisation of the interventions.

### Eligibility criteria

We included randomised controlled trials on all types of de-implementation interventions (both individual and multiple strategy interventions) conducted in primary care. Eligible studies compared a de-implementation intervention with a placebo or sham intervention, or usual care, or with another de-implementation intervention. We included any target group, such as patients with any disease, as well as all healthcare professionals, organisations, and members of the public.

We excluded deprescribing trials, trials only aiming to reduce the use of resources (eg, financial resources or clinical visits), and trials where medical practice, such as laboratory tests, was used as the intervention to reduce the use of another practice (online supplemental methods 2).^5^ We excluded trials that mainly studied paediatric practices (ie, trials were excluded if they reported that most participants were children; trials were included if children were involved but comprised <50% of the study population). We excluded a trial if we did not find a full text manuscript for a conference abstract. Online supplemental methods 2 has further details on the inclusion and exclusion criteria.

### Outcomes and variables

Based on the taxonomy developed in our previous scoping review,^5^ we categorised outcomes from the included trials as use of low value care, total volume of care, appropriate care, and health outcomes. We categorised outcomes as low value care if the outcome was clearly defined to include only low value care or inappropriate care, and otherwise as total volume of care, including potentially both inappropriate and appropriate care. For appropriate care, we used outcomes defined as appropriate care or care in accordance with guidelines, as defined by the authors of the original articles. Also, we aimed to record data on mortality, morbidity, quality of life, and use of healthcare facilities. If outcome data needed for the meta-analysis for low value care and total volume of care outcomes were missing, we contacted the study authors for data and added the data to the meta-analysis.

If outcomes were measured at multiple time points, we extracted data for the last measurement before one year (short term follow-up) and for the last measurement at or beyond one year (long term follow-up). We also collected and evaluated year of publication, unit of randomisation allocation (individual *v* cluster), number of clusters, length of follow-up, number of study participants, intervention categories, risk of bias, implementation theory used, tailoring the de-implementation intervention to study context, and baseline imbalances.

### Study selection and data extraction

We developed standardised forms with detailed guidance for screening abstracts and full texts, extracting the data, and assessing risk of bias. For each study report, two methodologically trained reviewers used these forms to independently screen for eligibility, extract the data, and assess risk of bias.^10^ Disagreements were resolved by discussion between the two reviewers and, if necessary, with an adjudicator (clinician methodologist).

We predefined intervention categories before analysis based on intervention details extracted with a modified TIDieR (template for intervention description and replication) checklist (online supplemental methods 3). Intervention categories were provider education, audit and feedback, decision support, and patient education (online supplemental methods 3). Strategies that did not align with the predefined categories were grouped into an others category and summarised narratively.

### Risk of bias

Informed by previous literature^11 12^ and through iterative discussions and consensus building, we modified the Cochrane risk of bias tool for cluster randomised controlled trials (online supplemental methods 4).^13^ Studies were independently rated in duplicate based on six criteria: randomisation procedure, allocation concealment, blinding of outcome collection, missing outcome data, contamination, and selective reporting. We judged studies to be at a high or low risk of bias for each criterion.

### Analysis

We summarised continuous outcomes as means with standard deviations, and binary outcomes as proportions. When at least three studies reported odds ratios, mean differences, arm specific event rates, or mean values with variance estimates, we conducted a random effects meta-analysis with the DerSimonian-Laird approach with Hartung-Knapp adjustment. We used the meta package in R statistics to perform the meta-analysis and draw the forest plots.^14^ The full code and all data used in the analysis are available at the Open Science Framework.^15^

We reported low value care and total volume of care as both continuous and binary outcomes. To provide one summary estimate, we used odds ratios to represent the treatment effect for continuous and binary outcomes at the final time point measured. We converted effect estimates from continuous variables to standardised mean differences, and subsequently to odds ratios following the guidance of the Cochrane handbook (online supplemental methods 5).^16^ If low value care outcomes were not reported, we used the total volume of care outcome as an indirect estimate for low value care. If treatment effects and confidence intervals (CIs) were not reported, these were estimated with arm specific estimates, including post-intervention means and standard deviations for continuous outcomes and event rates for binary outcomes.^17^ For cluster randomised controlled trials, if effect estimates did not account for clustering or only arm specific estimates were available, we adjusted for clustering (online supplemental methods 5). We calculated the I^2^ statistic and τ^2^ to analyse the heterogeneity in the results. Furthermore, we calculated 95% prediction intervals to estimate the expected range of effects in different settings.^18^

### Subgroup and sensitivity analyses

If at least three trials in the same intervention category and similar control arms were available, we conducted subgroup analyses on randomisation unit, tailoring, theoretical background, and intensity (online supplemental methods 5). We prespecified hypotheses that effect sizes would be larger for cluster randomised trials (*v* individually randomised), tailoring (*v* not tailoring), using theoretical background (*v* not using theoretical background), and higher intensity (*v* lower intensity).

If at least three trials were available in each subgroup, we conducted sensitivity analyses on broader intervention categories (comparing with no intervention only), binary and continuous variables separately (*v* combined), and baseline differences (excluding trials with >10% baseline differences or small trials without baseline periods *v* not excluding these trials). Because large trials with continuous variables may translate to non-intuitive estimates of odds ratios, we conducted meta-analyses with the ratio of means if at least three trials of a specific type of intervention had continuous outcomes.^19^ Online supplemental methods 5 provides the details of the analyses.

### Certainty of evidence

Two methodologically trained reviewers assessed the certainty of the evidence (also known as quality of evidence) with the Grading of Recommendations Assessment, Development, and Evaluation (GRADE) approach.^20^,^24^ We assessed the credibility of subgroup effects with the Instrument to Assess the Credibility of Effect Modification Analyses (ICEMAN).^25^

### Patient and public involvement

Patients and members of the public were not involved in the design or conduct of this study. This was a systematic review for which patient and public involvement was not feasible. Results will be shared with participants via lay summaries and more broadly with the public through press releases, institutional websites, and social media.

## Results

**Figure 1:**
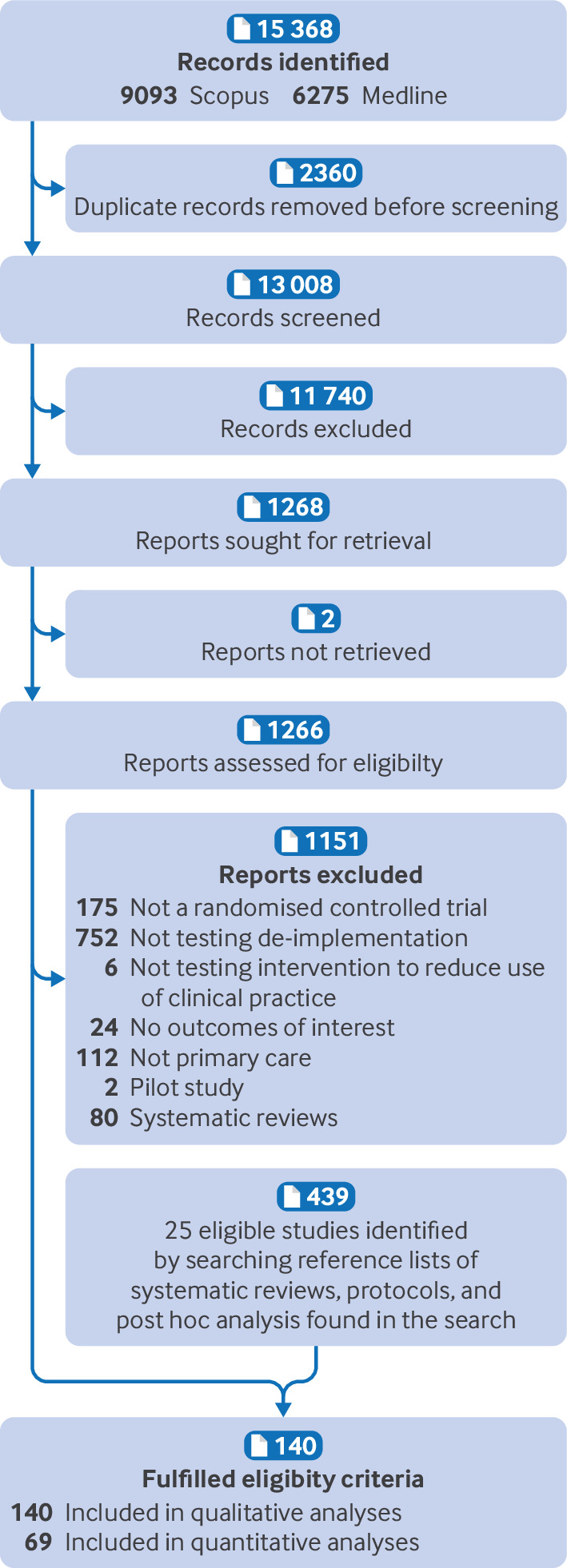
Study flowchart of studies identified from databases and registers

After removing duplicates, we screened 13 008 abstracts and identified 1268 potentially eligible publications, of which 115 trials proved eligible after full text screening. We also included 25 trials after hand searching 439 systematic reviews, protocols, and post hoc analyses, giving a total of 140 trials (figure 1). Finally, after requesting missing data from study authors, we found that 97 trials provided sufficient data for the meta-analysis. We included 69 trials in the quantitative analyses because 28 trials did not have three or more comparable trials for the meta-analysis (figure 1 and online supplemental tables 1 and 2).

### Study characteristics

Of the 140 trials included in the study, 109 (78%) were cluster trials and 31 (22%) were individually randomised trials; 91 (65%) trials compared de-implementation interventions with no intervention and 49 (35%) with another intervention; 37 (26%) trials measured low value care outcomes, 122 (87%) total volume of care, 17 (12%) appropriate care, and 14 (10%) health outcomes. Sixty two (44%) trials used binary outcomes and 78 (56%) continuous outcomes to measure use of low value care. Of the trials that used binary outcomes, two thirds (n=42) aimed to reduce the use of antibiotics, other drug treatments (n=8), imaging (n=9), and laboratory testing (n=5). Of the trials that used continuous outcomes, 33 aimed to reduce the use of antibiotics, other drug treatments (n=34), imaging (n=8), and laboratory testing (n=10). Median follow-up was 287 days (interquartile range 180-365). Median number of providers was 158 (interquartile range 81-372). Online supplemental tables 1-12 provide details of the characteristics of the 140 trials included in the study, such as number of participants (providers and patients) and outcomes. Online supplemental tables 1-12 also provide information on specific study and intervention characteristics with estimated relative effects (ratio of means or relative risk).

### Risk of bias and certainty of the evidence

Of the 140 trials, 139 (99%) had adequate randomisation sequence generation; 95 (68%) had adequate allocation concealment; 114 (81%) had blinded outcome collectors or the data were collected from a database or registry; 63 (45%) had little or no missing data; 109 (78%) had a low risk of contamination; and 131 (94%) had no risk of selective reporting (online supplemental figure 1). We judged all domains as having a low risk of bias (ie, no domain with a high risk of bias) for 26 (19%) trials, one domain with a high risk of bias for 60 (43%) trials, two domains with a high risk of bias for 36 (26%) trials, and three or more domains with a high risk of bias for 18 (13%) trials. The certainty of evidence is presented separately for each intervention category (online supplemental table 13).

### Impact on use of low value care

#### Provider education

**Figure 2:**
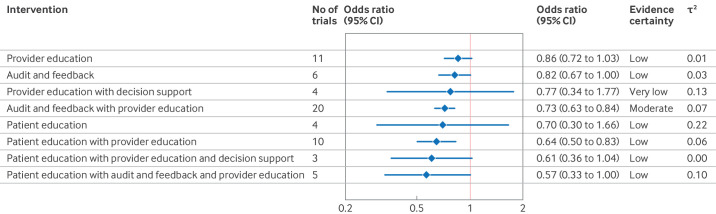
Effectiveness of de-implementation strategies compared with no intervention in reducing the use of low value care. CI=confidence interval

**Figure 3:**
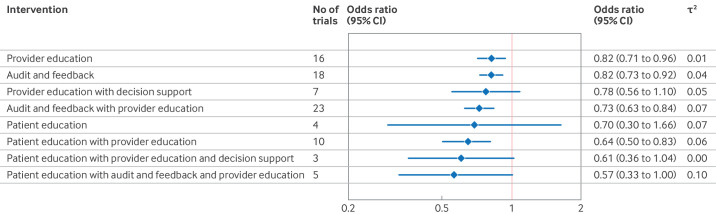
Sensitivity analysis: effectiveness of de-implementation strategies compared with no intervention or educational materials in reducing the use of low value care. CI=confidence interval

Eleven studies^26^,^36^ measured the effect of provider education (*v* no intervention) on low value care (table 1) with a median follow-up of 365 days (interquartile range 165-519). Pooled analysis suggested that provider education may slightly reduce the use of targeted low value care (odds ratio 0.86, 95% CI 0.72 to 1.03; I^2^=36%; τ^2^=0.01; prediction interval 0.65 to 1.15; low certainty of evidence; figure 2 and table 1). Sensitivity analysis, in which the distribution of educational materials was included in the no intervention group, provided similar results (16 trials, odds ratio 0.82, 95% CI 0.71 to 0.96; τ^2^=0.01) (figure 3). Post hoc analysis of trials directly comparing educational meetings versus educational materials suggested a potentially larger reduction in the use of targeted low value care with educational meetings (odds ratio 0.76, 95% CI 0.55 to 1.04; I^2^=30%; τ^2^=0.03). Online supplemental figures 2-8 provide forest plots of all analyses of provider education.

**Table 1:** Pooled effect estimates and certainty of evidence for use of low value care

Intervention (No of studies)	Odds ratio (95% CI)	τ^2^	Estimated relative change (95% CI)*	Certainty of evidence†	Summary
Provider education (n=11)	0.86 (0.72 to 1.03)	0.01	−10% (−21% to 2%)	Low	Provider education may decrease the use of low value care slightly
Audit and feedback (n=6)	0.82 (0.67 to 1.00)	0.03	−13% (−25% to 0%)	Low	Audit and feedback may decrease the use of low value care slightly
Provider education with decision support (n=4)	0.77 (0.34 to 1.77)	0.13	−16% (−55% to 37%)	Very low	Evidence on provider education combined with decision support is very uncertain
Provider education with audit and feedback (n=20)	0.73 (0.63 to 0.84)	0.07	−23% (−32% to −13%)	Moderate	Provider education combined with audit and feedback likely decreases the use of low value care
Patient education (n=4)	0.70 (0.30 to 1.66)	0.22	−16% (−51% to 21%)	Low‡	Patient education may decrease the use of low value care slightly
Patient education with provider education (n=10)	0.64 (0.50 to 0.83)	0.06	−30% (−43% to −13%)	Low	Provider education combined with patient education may decrease the use of low value care
Patient education combined with provider education and decision support (n=3)	0.61 (0.36 to 1.04)	0.00	−31% (−56% to 3%)	Low	Provider education combined with patient education and decision aids may decrease the use of low value care
Patient education combined with audit and feedback and provider education (n=5)	0.57 (0.33 to 1.00)	0.10	−34% (−58% to 0%)	Low	Provider education combined with patient education and audit and feedback may decrease the use of low value care

* We estimated relative change in two steps. We first calculated the relative risk (RR) based on the median control risk of having low value care at follow-up within each intervention category. We then calculated relative change as (1−RR)×100.

† Online supplemental table 13 reports specific assessment of the certainty of the evidence for each intervention.

‡ By excluding one trial with a control arm that received education on viral infections (initially thought to have no effect), pooled odds ratio was 0.51 (95% CI 0.29 to 0.91). We included the trial but did not rate down for inconsistency.

CI, confidence interval.

#### Audit and feedback

Six studies^3037^,^41^ measured the effect of audit and feedback (*v* no intervention) on low value care with a median follow-up of 365 days (interquartile range 297-639). The pooled results suggested that audit and feedback may slightly reduce the use of targeted low value care (odds ratio 0.82, 95% CI 0.67 to 1.00; I^2^=84%; τ^2^=0.03; prediction interval 0.48 to 1.40; low certainty of evidence; figure 2 and table 1). A sensitivity analysis with broader intervention categories suggested a similar effect (18 trials, odds ratio 0.82, 0.73 to 0.92; τ^2^=0.04; figure 3). Online supplemental figures 9-11 provide forest plots for all analyses of audit and feedback.

#### Provider education with decision support

Pooled results (four trials^42^,^45^) suggested that provider education combined with decision support (*v* no intervention) may reduce the use of targeted low value care, but the certainty of the evidence was very low (odds ratio 0.77, 95% CI 0.34 to 1.77; I^2^=54%; τ^2^=0.13; prediction interval 0.12 to 5.00; very low certainty of evidence; figure 2 and table 1). Median follow-up time was 273 days (interquartile range 180-639). Sensitivity analysis, where the distribution of educational materials was included in the no intervention group, gave similar results (seven trials, odds ratio 0.78, 95% CI 0.56 to 1.10; τ^2^=0.05) (figure 3). Online supplemental figures 12 and 13 provide forest plots for all analyses of provider education combined with decision support.

#### Provider education with audit and feedback

In the pooled analysis (20 trials^3646^,^64^), provider education combined with audit and feedback (*v* no intervention) probably reduced the use of targeted low value care (odds ratio 0.73, 95% CI 0.63 to 0.84; I^2^=86%; τ^2^=0.07; prediction interval 0.41 to 1.30; moderate certainty of evidence; figure 2 and table 1). Median follow-up time was 365 days (interquartile range 180-365). Sensitivity analysis comparing education combined with audit and feedback with distribution of educational materials suggested a similar effect (23 trials, odds ratio 0.73, 95% CI 0.63 to 0.84; τ^2^=0.07; figure 3). Online supplemental figures 14-21 provide all analyses of provider education combined with audit and feedback.

#### Patient education

Four trials^65^,^68^ measured the effect of patient education (*v* no intervention) on low value care with a median follow-up of 24 days (interquartile range 7-63). Pooled analysis suggested that patient education may decrease the use of targeted low value care (odds ratio 0.70, 95% CI 0.30 to 1.66; I^2^=62%; τ^2^=0.22; prediction interval 0.07 to 7.47; low certainty of evidence; figure 2 and table 1). Post hoc sensitivity analysis excluding one trial^67^ comparing low value care education with education on viral infections suggested a slightly larger effect (odds ratio 0.52, 95% CI 0.30 to 0.89; τ^2^=0.00). Online supplemental figures 22 and 23 provide forest plots for all analyses of patient education.

#### Patient education with provider education

Pooled analysis (10 trials^69^,^78^) suggested that provider education combined with patient education (*v* no intervention) may reduce the use of targeted low value care (odds ratio 0.64, 95% CI 0.50 to 0.83; I^2^=61%; τ^2^=0.06; prediction interval 0.34 to 1.21; low certainty of evidence; figure 2 and table 1). Median follow-up time was 240 days (interquartile range 120-365). Online supplemental figures 24-27 show forest plots for all analyses of provider education combined with patient education.

#### Patient education with provider education and decision support

Pooled results (three trials^79^,^81^) suggested that provider education combined with decision support and patient education (*v* no intervention) may reduce the use of targeted low value care (odds ratio 0.61, 95% CI 0.36 to 1.04; I^2^=0%; τ^2^=0.00; prediction interval 0.007 to 5.36; low certainty of evidence; figure 2, table 1, and online supplemental figures 28). Median follow-up time was 150 days.

#### Patient education with provider education and audit and feedback

Pooled analysis (five trials^4882^,^85^) suggested that provider education combined with audit and feedback and patient education (*v* no intervention) may reduce the use of targeted low value care (odds ratio 0.57, 95% CI 0.33 to 1.00; I^2^=77%; τ^2^=0.10; prediction interval 0.18 to 1.00; low evidence of evidence; figure 2 and table 1). Median follow-up time was 180 days (interquartile range 180-365). Online supplemental figures 29 and 30 show forest plots for all analyses on provider education combined with audit and feedback and patient education.

The heterogeneity of results was moderate or high in all intervention categories. Prediction intervals were wide, including the null effect in all intervention categories, which suggests that the impact likely varies across different settings and specific interventions. Online supplemental table 14 provides absolute effect estimates for each intervention category (provider education, audit and feedback, patient education, and their combinations).

### Appropriate care and health outcomes

Of all trials, 17 measured appropriate care (online supplemental table 15). Three or more trials were not available for any of the intervention categories and we therefore did not conduct any meta-analyses for appropriate care or health outcomes. Of the 17 trials, 16 (94%) found either no effect or an increase in the use of appropriate care after a de-implementation intervention (online supplemental table 15). Of all 14 trials reporting health outcomes (online supplemental table 16), seven measured repeat consultations or visits to the emergency department after the de-implementation intervention, suggesting either a small decrease or no effect. Other health outcomes were not measured in more than one trial (online supplemental table 16).

### Subgroup analysis

#### Cluster versus individually randomised trials

Cluster randomised trials suggested a moderate impact of provider education combined with audit and feedback (odds ratio 0.65, 95% CI 0.57 to 0.73; τ^2^=0.01) whereas individually randomised trials suggested no effect (odds ratio 1.00, 0.87 to 1.15; τ^2^=0.00; P value for interaction <0.001; low credibility for subgroup effect; online supplemental figure 21).

#### Intensity (lower *v* higher)

Trials assessing the effectiveness of provider education combined with audit and feedback showed a greater impact with higher intensity interventions (odds ratio 0.59, 95% CI 0.47 to 0.74; τ^2^=0.02) than with lower intensity interventions (odds ratio 0.82, 0.71 to 0.95; τ^2^=0.06; P value for interaction 0.006; moderate credibility for subgroup effect; online supplemental figure 16). In trials assessing the effectiveness of provider education, we found no credible subgroup effects for educational meetings (odds ratio 0.78, 95% CI 0.38 to 1.60; τ^2^=0.09) versus educational materials (odds ratio 0.85, 0.69 to 1.06; τ^2^=0.01; P value for interaction 0.70; online supplemental figure 3).

#### Tailoring

For provider education, and provider education combined with audit and feedback intervention categories, tailored interventions did not have credible subgroup effects (online supplemental figures 4 and 17). Tailored interventions of provider education combined with patient education (*v* interventions without tailoring), however, were associated with larger reductions in the use of targeted low value care (odds ratio 0.55, 95% CI 0.42 to 0.73, τ^2^=0.05 *v* odds ratio 0.83, 0.47 to 1.49, τ^2^=0.07, P value for interaction 0.05; very low credibility for subgroup effect; online supplemental figure 26).

#### Theoretical background

For provider education, provider education combined with patient education, and provider education combined with audit and feedback intervention categories, theory based interventions did not have credible subgroup effects on targeted low value care (online supplemental figures 5, 18, and 27).

### Sensitivity analysis

In sensitivity analyses, trials with continuous variables (*v* all trials, including binary and continuous outcomes) suggested a smaller effect for provider education combined with audit and feedback. For other intervention categories (provider education, audit and feedback, and both combined with patient education), we found no differences, or any potential differences could be explained by chance (online supplemental figure 31A). Both sensitivity analyses, one of trials with binary variables and another of trials without baseline imbalances, suggested a similar effect (online supplemental figure 31B and 31C).

### Trials excluded from the meta-analysis

Fourty three trials were not eligible for the meta-analysis because of missing data. Online supplemental table 12 provides details of these trials.

## Discussion

### Principal findings

We conducted a comprehensive systematic review and meta-analysis of randomised controlled trials of de-implementation interventions in primary care, identifying 140 randomised controlled trials. Overall, we found that de-implementation strategies reduced the relative risk of targeted low value care by 10-35% (median length of follow-up 287 days). With multi-strategy interventions, including patient education, we found relative risk reductions of 30-35% in the use of low value care. The certainty of the evidence was moderate for provider education combined with audit and feedback but low or very low for other interventions (provider education, audit and feedback, patient education, decision support, and combinations of these strategies).

### Comparison with other studies

To our knowledge, no comprehensive systematic review and meta-analysis exists of randomised controlled trials of de-implementation interventions in primary care. A previous systematic review on the effects of social norm feedback on antibiotic prescribing included seven randomised controlled trials and three non-randomised studies in their meta-analysis.^86^ The results suggested a 4% absolute risk reduction in antibiotic prescribing. In contrast, we studied individual and multi-strategic interventions separately. In our study, audit and feedback decreased the absolute risk of targeted low value care by 4% (six randomised controlled trials; low certainty of evidence), provider education combined with audit and feedback by 7% (20 randomised controlled trials; moderate certainty of evidence), and patient education combined with audit and feedback and provider education by 11% (five randomised controlled trials; low certainty of evidence).

Another systematic review and meta-analysis measured the effectiveness of engaging patients in decision making to reduce low value care.^87^ This meta-analysis included eight randomised controlled trials (mostly testing some form of patient education) and six non-randomised studies. The results suggested a 31% relative decrease in the use of targeted low value care. In our study, we applied a more specific categorisation of interventions, analysing individual and multi-strategic approaches separately. In our analysis (19 randomised controlled trials), with patient education alone, we found a 16% relative reduction in the use of targeted low value care (four randomised controlled trials; low certainty of evidence). With patient education combined with provider education, however, we saw a 30% reduction (10 randomised controlled trials; low certainty of evidence) in the use of targeted low value care, and with patient education combined with both provider education and audit and feedback, a 34% relative decrease (five randomised controlled trials; low certainty of evidence) in targeted low value care. Also, in agreement with our results, some systematic reviews have suggested that multicomponent intervention had a greater impact than individual strategy interventions.^6^,^8^ These reviews, however, did not include 85 of the trials in our review, did not provide pooled estimates, and did not assess the certainty of the evidence for specific intervention types.

### Study implications

De-implementation begins with recognising prevalent low value practices and identifying strategies with the greatest potential to reduce these practices.^88 89^ Our results showed that achieving a meaningful reduction in the use of low value care may require more than one strategic approach. We found moderate certainty of evidence for interventions combining provider education with audit and feedback. The estimated 23% relative risk reduction in the use of targeted low value care with these interventions may be further enhanced by increasing the intensity of the interventions.

Provider education, including seminars, meetings, and educational materials, is the most common quality improvement strategy in healthcare. We found low certainty of evidence that provider education resulted in a 10% relative risk reduction in the use of targeted low value care (table 1). Given the magnitude and uncertainty of its impact, along with the resources required for educational activities, this strategy may have limited effectiveness in reducing the use of low value care.

We found a 16% relative risk reduction for patient education only and a 30-34% relative risk reduction for patient education combined with provider education and other strategies in the use of targeted low value care. Despite the low certainty of the evidence, implementing patient education more often should be considered. At a minimum, future research should help in finding applicable evidence on patient education strategies. Current evidence does not allow strong conclusions to be drawn on the effect of different types of patient education strategies. Trials that successfully reduced low value care typically provided information on the benefits and harms of the management options as well as information on the natural course of the disease.^65 66 70 71 75 85^

Although potentially having a greater effect, multifaceted interventions (ie, interventions including multiple strategies) typically had a low certainty of evidence. Firstly, the heterogeneity of the study results was high, likely explained, at least in part, by the variety of interventions and study contexts. Secondly, concerns were raised about the directness of the evidence of the multifaceted interventions.^5^ For example, a decrease in the use of low value care after educational sessions could also be dependent on the characteristics of the person delivering the intervention. The intervention might therefore not work if given by other people with different personal characteristics. Researchers and other decision makers, however, have at least two approaches to navigate these concerns: choosing effective and reproducible interventions (from the randomised controlled trials) with the highest applicability and measuring the local impact of de-implementation interventions on low value care. Online supplemental tables 1–10 provide detailed lists for choosing specific scalable interventions with the greatest potential impact. Our analysis suggested very uncertain benefits from tailoring. Therefore, tailored interventions might serve as a secondary choice, but if implemented, measuring their local impact becomes particularly important.

### Strengths and limitations of this study

Our review had several strengths. Firstly, we conducted a scoping review of randomised controlled trials of de-implementation interventions to plan the systematic review and meta-analysis.^5^ Secondly, we applied a comprehensive search strategy and found 140 randomised controlled trials conducted in primary care, about twice as many as in the most comprehensive systematic review published in 2023 that included 76 trials conducted in primary care but meta-analyses were not performed.^6^ Thirdly, we conducted a duplicate assessment of study eligibility and data extraction, risk of bias, as well as categorisation of interventions according to the modified TIDieR checklist (online supplemental methods 3). Finally, we conducted a meta-analysis with appropriate statistical methods^14^ and evaluated the certainty of the evidence according to the GRADE approach, unlike previous systematic reviews.

Limitations of this study were mostly related to the available literature. Firstly, despite contacting the study authors, 43 (31%) of the 140 randomised controlled trials included in our study did not report enough data to be included in the meta-analysis. Secondly, because the randomised controlled trials included used both continuous and binary outcomes, we converted continuous outcomes to odds ratios. This method may have resulted in larger effect sizes and smaller weight in the meta-analyses because effect sizes are dependent on both standard deviation and magnitude of effect. We therefore conducted sensitivity analyses separately for binary and continuous outcomes, which showed no large differences. Thirdly, we could not generate precise estimates for specific time points, which may not represent a major limitation. Seven trials provided both short term and long term follow-up data.^30 31 36 62 70 74 84^ In four of these trials, point estimates were similar, in two trials, point estimates were smaller (indicating a higher impact) for short term follow-up, and in one trial, point estimates were smaller for long term follow-up. Based on this limited evidence, the effect of the length of follow-up is uncertain but is probably not substantial. Fourthly, most meta-analyses provided only low to very low certainty of evidence. Fifthly, we had a limited number of direct comparisons between different de-implementation strategies, which could be explored further in future research (eg, by conducting a network meta-analysis to compare the effectiveness of various strategies). Finally, of the 140 randomised controlled trials, only 14 (10%) reported an effect on patient health outcomes and 17 (12%) on appropriate care. None of these 14 randomised controlled trials found a negative effect on patient health outcomes, and only one of 17 randomised controlled trials indicated a potential decrease in appropriate care. Future randomised controlled trials should focus on replicable and pragmatic approaches to de-implementation strategies and include several different contexts in the evaluation of the effectiveness of the interventions.

### Conclusions

Our comprehensive systematic review and meta-analysis of randomised controlled trials on de-implementation interventions found a moderate certainty of evidence that with provider education combined with audit and feedback the use of targeted low value care was reduced. Also, we found low certainty of evidence suggesting that patient education combined with provider education and other strategies reduced the use of targeted low value care. The evidence suggested limited effectiveness of provider education in reducing targeted low value care. These results, together with understanding of the local patterns of low value care, could be helpful when deciding on de-implementation strategies.

## Supplementary material

10.1136/bmjmed-2025-001343 online supplemental file 1

## Data Availability

Data are available in a public, open access repository.
