# Supplementary material for: Effectiveness of different de-implementation strategies in primary care: systematic review and meta-analysis
Source: BMJ Med. 2025 Sep 9;4(1):e001343. doi: 10.1136/bmjmed-2025-001343 (PMC12421606; doi:10.1136/bmjmed-2025-001343)
Supplement: online supplemental file 1 [file bmjmed-4-1-s001.pdf]

Supplementary material

Table of Contents

Methods 1. Search strategy ... 3

Methods 2. Eligibility criteria – specifications ... 6

Methods 3. Intervention categories – modified TIDieR checklist ... 7

Methods 4. Risk of Bias ... 9

Methods 5. Analysis specifications ... 14

Figure 1. Risk of Bias ... 16

Figure 2. Provider education – Forest plot ... 17

Figure 3. Subgroup analysis – Educational material vs educational meetings ... 17

Figure 4. Subgroup analysis – tailoring vs no tailoring of the intervention ... 18

Figure 5. Subgroup analysis – with vs without theoretical background ... 18

Figure 6. Sensitivity analysis – Provider education vs no intervention/educational materials 19

Figure 7. Sensitivity analysis – Trials with binary outcome ... 19

Figure 8. Sensitivity analysis – Trials with continuous outcome ... 20

Figure 9. Audit and feedback – forest plot ... 21

Figure 10. Sensitivity analysis – Audit and feedback with or without educational material vs no intervention or educational materials ... 21

Figure 11. Sensitivity analysis – Trials with continuous outcome ... 22

Figure 12. Provider education combined with decision support – forest plot ... 22

Figure 13. Sensitivity analysis – Provider education combined with decision support versus no intervention or educational materials ... 22

Figure 14. Audit and feedback combined with provider education – forest plot ... 23

Figure 15. Sensitivity analysis – Audit and feedback combined with provider education vs no intervention or educational materials ... 24

Figure 16. Subgroup analysis – low vs high intensity ... 25

Figure 17. Subgroup analysis – tailoring vs no tailoring of the intervention ... 26

Figure 18. Subgroup analysis – with vs without theoretical background ... 27

Figure 19. Sensitivity analysis – trials with binary outcome ... 28

Figure 20. Sensitivity analysis – trials with continuous outcome ... 28

Figure 21. Sensitivity analysis – randomization unit ... 29

Figure 22. Patient education – forest plot ... 30

Figure 23. Sensitivity analysis – without one trial including control of education on viral infections ... 30

Figure 24. Patient education and provider education combined – forest plot ... 31

Figure 25. Sensitivity analysis – trials with binary outcome 31
Figure 26. Subgroup analysis – tailoring versus no tailoring 32
Figure 27. Subgroup analysis – theoretical background versus no theoretical background 32
Figure 28. Patient education combined with provider education and decision support 33
Figure 29. Patient education combined with audit and feedback, and provider education 33
Figure 30. Sensitivity analysis – trials with continuous outcome 33
Figure 31. Sensitivity analyses on continuous and binary outcomes as well as trials with no imbalance 34
Table 1. Study characteristics – Other interventions 35
Table 2. Study characteristics - trials with other control groups 39
Table 3. Study characteristics - Provider education 43
Table 4. Study characteristics – Audit and feedback 45
Table 5. Study characteristics – Provider education combined with decision support 46
Table 6. Study characteristics – Provider education combined with audit and feedback 47
Table 7. Study characteristics – Patient education 51
Table 8. Study characteristics – Patient education combined with provider education 52
Table 9. Study characteristics – Patient education combined with provider education and decision support 55
Table 10. Study characteristics – Patient education combined with audit and feedback, and provider education 56
Table 11. Study characteristics – studies with educational material as control 58
Table 12. Study characteristics – Trials ineligible for the meta-analysis 61
Table 13. Evidence certainty assessment 73
Table 14. Estimated absolute effects (risk difference and 95% confidence interval) for different baseline risks 75
Table 15. Studies with appropriate care outcomes 76
Table 16. Studies with health and healthcare utilization outcomes 79

Methods 1. Search strategy

Scopus

(((TITLE-ABS-KEY ("randomized controlled trial")) OR (TITLE-ABS-KEY ("controlled clinical trial")) OR (ABS (randomized OR placebo OR randomly)) OR (TITLE (trial))) AND (((TITLE-ABS-KEY (abandon* OR de-list* OR dis-invest* OR discontinu* OR dis-continu* OR decommiss* OR de-commiss* OR deadopt* OR de-adopt* OR de-implement* OR deimplement* OR reduc* OR remov* OR stop* OR relinquish* OR disadopt* OR disinvest* OR de-prescri* OR deprescri*)) OR (TITLE-ABS-KEY ((decreas* W/3 "use") OR (declin* W/3 "use") OR (drop* W/3 "use") OR (decreas* W/3 rate*))) OR (TITLE-ABS-KEY ("change* in use" OR "change* in practice*")) OR (TITLE-ABS-KEY (improv* W/4 "use")) OR (TITLE-ABS-KEY (reduc* W/4 "use")) OR (TITLE-ABS-KEY (change* W/4 "use" OR change* W/3 practice*))) AND ((TITLE-ABS-KEY (inappropriate W/3 prescri*)) OR (TITLE-ABS-KEY ("Health Service*" W/1 misus*)) OR (TITLE-ABS-KEY (low-value)) OR (TITLE-ABS-KEY ((overutili* OR overus* OR overdiagnos* OR overtreat* OR overmedicat* OR overprescrib*))) OR (TITLE-ABS-KEY ((unnecessary OR ineffective OR useless OR inefficient OR valueless) W/1 (care OR usage OR utilisation OR utilization OR treatment* OR intervention* OR practice* OR procedure OR drug* OR therap* OR technolog* OR device* OR surg* OR test* OR lab* OR imaging))) OR (TITLE-ABS-KEY ("unnecessary use")) OR (TITLE-ABS-KEY ("INAPPROPRIATE use")) OR (TITLE-ABS-KEY (obsolete))))) OR (((TITLE-ABS ("randomized controlled trial")) OR (TITLE-ABS ("controlled clinical trial")) OR (TITLE-ABS (randomized)) OR (TITLE-ABS (placebo)) OR (TITLE-ABS (randomly)) OR (TITLE (trial))) AND ((TITLE-ABS-KEY ("Unnecessary Procedure*")) OR (TITLE-ABS-KEY ("prescription rate*")) OR (TITLE-ABS-KEY ("Guideline adherence")) OR (TITLE-ABS-KEY ("too much medicine")) OR (TITLE-ABS-KEY ("choosing wisely")) OR (TITLE-ABS-KEY ("do not do")) OR (TITLE-ABS-KEY (deprescription*)) OR (TITLE-ABS-KEY (reduc* W/4 prescri*)) OR (TITLE-ABS-KEY (unnecessary W/4 "use")) OR (TITLE-ABS-KEY (unnecessary W/4 prescri*)) OR (TITLE-ABS-KEY ("Inappropriate prescri*")) OR (TITLE-ABS-KEY (decreas* W/3 referrals)))))

Medline

Search Strategy:

1. Unnecessary Procedures.mp.
2. prescription rate*.tw.
3. Guideline adherence.tw.
4. too much medicine.tw.
5. choosing wisely.tw.
6. "do not do".tw.
7. deprescriptions.mp.
8. (guideline* adj3 implementation*).tw.
9. Practice Patterns, Physicians'/sn, st
10. Physicians, Family/st, sn [Standards, Statistics & Numerical Data]
11. exp Drug Utilization/sn [Statistics & Numerical Data]
12. randomized controlled trial.pt.
13. controlled clinical trial.pt.
14. randomized.ab.
15. placebo.ab.
16. clinical trials as topic.sh.
17. randomly.ab.
18. trial.ti.
19. 12 or 13 or 14 or 15 or 16 or 17 or 18
20. exp animals/ not humans.sh.
21. 19 not 20
22. 1 or 2 or 3 or 4 or 5 or 6 or 7 or 9 or 10 or 11
23. 21 and 22
24. (reduc* adj4 prescri*).tw.
25. (unnecessary adj4 "use").mp.
26. (unnecessary adj4 prescri*).tw.
27. Inappropriate Prescribing/
28. (decreas* adj3 referrals).tw.
29. inappropriate prescri*.tw.
30. 24 or 25 or 26 or 27 or 28 or 29
31. 21 and 30
32. 23 or 31
33. exp Health Services Misuse/
34. Inappropriate Prescribing/
35. low-value.tw.
36. (overutili* or overus* or overdiagnos* or overtreat* or overmedicat* or overprescrib*).tw.
37. ((unnecessary or ineffective or useless or inefficient or valueless) adj (care or usage or utilisation or utilization or treatment* or intervention* or practice* or procedure or drug* or therap* or technolog* or device* or surg* or test* or lab* or imaging)).tw.
38. "unnecessary use".tw.
39. Inappropriate Prescri*.tw.
40. "INAPPROPRIATE use".tw.
41. obsolete.tw.
42. (contradict* or refute* or reassess* or re-assess* or re-apprais* or reapprais* or revers).tw.
43. exp Guideline Adherence/
44. 33 or 34 or 35 or 36 or 37 or 38 or 39 or 40 or 41
45. (abandon* or de-list* or dis-invest* or discontinu* or dis-continu* or decommiss* or de-commiss* or deadopt* or de-adopt* or de-implement* or deimplement* or reduc* or remov* or stop* or relinquish* or disadopt* or DISINVEST* or de-prescri* or Deprescri*).tw.
46. ((decreas* adj3 "use") or (declin* adj3 "use") or (drop* adj3 "use") or (decreas* adj3 rate*)).mp.
47. (withdraw* or replac* or reallocat* or re-allocat*).tw.
48. ("change* in use" or "change* in practice*").tw.
49. (improv* adj4 "use").mp.
50. ((change adj4 "use") or "change* in practice").mp.
51. (reduc* adj4 "use").tw.
52. 45 or 46 or 47 or 48 or 49 or 50 or 51
53. 44 and 52
54. 21 and 53

55. 32 or 54

Methods 2. Eligibility criteria – specifications

We excluded trials reporting only change scores or relative risks, or rate ratios from the meta-analysis (if no further data was provided after asking from the study authors), as they could not be pooled in the meta-analysis (1).

We excluded de-prescribing trials and trials using medical interventions (such as laboratory tests) to decrease use of another intervention. In a de-prescribing trial, researchers aim to help clinicians to stop medication already in use. Therefore, the aim is to cause new actions of de-prescription. We think the mechanism of change is fundamentally different to de-implementation, in which the aim is to prevent future action of prescribing (or using other medical practice). (2, 3)

1. Deeks JJ, Higgins JPT, Altman DG (editors). Chapter 10: Analysing data and undertaking meta-analyses. In: Higgins JPT, Thomas J, Chandler J, Cumpston M, Li T, Page MJ, Welch VA (editors). *Cochrane Handbook for Systematic Reviews of Interventions* version 6.4 (updated August 2023). Cochrane, 2023. Available from www.training.cochrane.org/handbook.
2. Steinman MA, Boyd CM, Spar MJ, Norton JD, Tannenbaum C. Deprescribing and deimplementation: Time for transformative change [published online ahead of print, 2021 Sep 9]. *J Am Geriatr Soc*. 2021;10.1111/jgs.17441.
3. Raudasoja AJ, Falkenbach P, Vernooij RWM, Mustonen JMJ, Agarwal A, Aoki Y, Blanker MH, Cartwright R, Garcia-Perdomo HA, Kilpeläinen TP, Lainiala O, Lamberg T, Nevalainen OPO, Raittio E, Richard PO, Violette PD, Komulainen J, Sipilä R, Tikkinen KAO. Randomized controlled trials in de-implementation research: a systematic scoping review. Implement Sci. 2022 Oct 1;17(1):65. doi: 10.1186/s13012-022-01238-z. PMID: 36183140; PMCID: PMC9526943.

Methods 3. Intervention categories – modified TIDieR checklist

**Intervention components**<br>Describe all used intervention components in detail.
**Providers**<br>Characteristics of the intervention providers.
**Number of providers**<br>if not applicable, answer "not applicable"
**Intensity**<br>Describe the timeline. How many times and in what timeline the intervention was delivered. Some interventions are continuous, which means that the intervention in use all the time. e.g. reminders in electronic records for specific patients
**Where**<br>Where the intervention was delivered?
**Comparison group**<br>Describe all comparison groups. e.g. placebo intervention, no intervention/care as usual, another intervention.<br>Specify the intervention, if there is placebo or another intervention in the comparison group.

Modified from: Hoffmann T C, Glasziou P P, Boutron I, Milne R, Perera R, Moher D et al. Better reporting of interventions: template for intervention description and replication (TIDieR) checklist and guide BMJ 2014; 348 :g1687 doi:10.1136/bmj.g1687

Intervention categories

Provider education

Educational materials or meetings for providers including physicians, nurses and other health care staff providing care. Could include educational reminders.

Patient education

Educational materials or face-to-face education for patients. Also includes providing educational content for communities or parents.

Audit and feedback

Including interventions auditing clinician performance and providing feedback in written or face-to-face formats. If a trial included separate educational meetings or educational material for providers, such as guideline distribution, the intervention was categorized as provider education combined with provider education.

Decision support

Interventions presenting pathway/algorithm to the clinician to help with treatment/diagnostic decisions. Could be in electronic or paper form.

Others

Strategies that did not align with the predefined categories were grouped into an 'others' category and summarized narratively.

Methods 4. Risk of Bias

1. **Randomization/imbalance of prognostic factors**

   a) **Was the allocation sequence adequately generated?**

Definitely yes (low risk of bias)	Probably yes	Probably no	Definitely no (high risk of bias)

The use of a random component should be sufficient for adequate sequence generation. This could be achieved by allocating interventions using methods such as repeated coin-tossing, throwing dice or dealing previously shuffled cards. If the allocation was by telephone or Internet, the randomization was done through a computer system.

<u>Examples of low risk of bias ("definitely yes"):</u> Referring to a random number table; Using a computer random number generator; Coin tossing; Shuffling cards or envelopes; Throwing dice; Drawing of lots; Minimization with or without a random element.

<u>Examples of high risk of bias ("definitely no"):</u> Sequence generated by odd or even date of birth; Sequence generated by some rule based on date (or day) of admission; Sequence generated by some rule based on hospital or clinic record number; Allocation by judgement of the clinician; Allocation by preference of the participant; Allocation based on the results of a laboratory test or a series of tests; Allocation by availability of the intervention.

If they say "randomized" and give no more information regarding sequence generation, the process was probably low risk of bias, so, answer "Probably yes".

   b) **Was the allocation adequately concealed?**

Definitely yes (low risk of bias)	Probably yes	Probably no	Definitely no (high risk of bias)

In randomized trials, allocation concealment strategies hide the method of sorting trial participants into treatment groups so that this knowledge cannot be exploited. Adequate allocation concealment serves to prevent trial investigators/recruiters from choosing treatment allocations for individuals/patients. Studies with poor allocation concealment (or none at all) are prone to selection bias. Trials where participants are recruited before randomization are low risk of bias. Trials where participants are recruited between randomization and the beginning of the intervention are usually high risk of bias.

If there are multiple levels of recruitment, consider allocation concealment on the lowest level. For instance, educational intervention targeted to physicians: clusters are health care centers, physicians are participants to whom the educational intervention was targeted and patients were participants to whom the medical intervention was targeted. If patients are recruited after the randomisation we will consider the study as high risk of bias. An exception is, however, if all patients from the physician were analysed or otherwise it was

impossible for the physician to decide which patients were included. In this case we will consider study as low risk of bias.

<u>Examples of low risk of bias ("definitely yes"):</u>

1) i) the unit of allocation was by patient or episode of care AND ii) there was some form of centralized randomization scheme and on-site computer system OR sealed opaque envelopes were used.
2) i) the unit of allocation was by institution, team or professional AND ii) less than 5% were recruited (at the lowest level) after the randomization
3) i) the unit of allocation was by institution, team or professional AND ii) Lowest level of recruitment was conducted after the randomization AND iii) recruiter(s) were blinded to study groups OR all participants were recruited

<u>Examples of low risk of bias ("probably yes"):</u>

1) i) the unit of allocation was by institution, team or professional AND ii) less than 10% were recruited (at the lowest level) after the randomization

<u>Examples of high risk of bias ("probably no"):</u>

1) the unit of allocation was by patient or episode of care and there was some form of centralized randomization scheme and allocation concealment was not reported.
2) the unit of allocation was by institution, team or professional and recruitment of study participants and It's unclear how many participants were recruited after the randomization.

<u>Examples of high risk of bias ("definitely no"):</u>

1) the unit of allocation was by patient or episode of care AND Using an open random allocation schedule (e.g. a list of random numbers) OR Assignment envelopes were used without appropriate safeguards (e.g. if envelopes were unsealed or non-opaque or not sequentially numbered) OR Alternation or rotation OR Date of birth OR Case record number OR Any other explicitly unconcealed procedure.
2) i) the unit of allocation was by institution, team or professional AND ii) allocation was not performed for all at the start of the study (over 10% recruitment of study participants after the randomization)

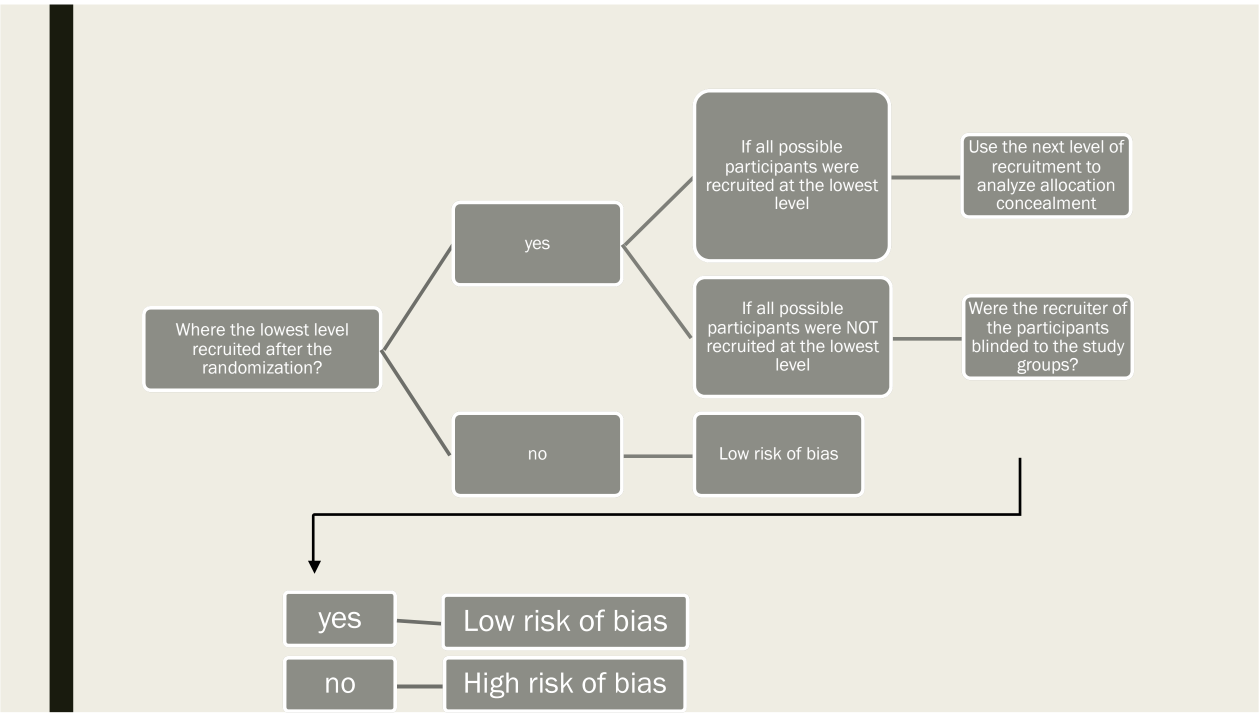

2. **Blinding. Was knowledge of the allocated interventions adequately prevented?**

Were data collectors/outcome assessors blinded?

Definitely yes (low risk of bias)	Probably yes	Probably no	Definitely no (high risk of bias)

<u>Low risk of bias ("definitely yes"):</u>
Data were collected from medical records or other database and blinding of data collectors was reported. In other situations, if data collectors did not know to which group study participants belong.
<u>Low risk of bias ("probably yes"):</u>
Data were collected from medical records or other database and blinding of data collectors was not reported.
<u>High risk of bias ("probably no"):</u>
In trials where data were not collected from medical records or other database, if blinding was not reported.
<u>High risk of bias ("definitely no"):</u>
Data were collected from medical records or other database and it was somehow stated that data collectors were not blinded or non-blinding is obvious other way.
In other situations, if non-blinding was reported.

If there were several ways of collecting the data, assess the way that primary outcome(s) was collected. If there was no reported primary outcome(s), primarily assess regarding the outcome of prevalence of low-value care/total volume of care was collected and secondarily assess regarding the outcome of intention/perception to reduce low-value care.

3. **Missing data (by primary outcome):**

Definitely yes (high risk of bias)	Probably yes	Probably no	Definitely no (low risk of bias)

Thresholds for risk of bias judgements. Respond:

<u>"Definitely no":</u>

Less than 5% of the primary outcome data is missing (low risk of bias)

<u>"Probably no":</u>

At least 5% but less than 10% of the outcome data is missing (low risk of bias)

<u>"Probably yes":</u>

At least 10% but less than 20% of the outcome data is missing (high risk of bias)

<u>"Definitely yes":</u>

At least 20% of the outcome data is missing (high risk of Bias)

Preferably use the primary outcome for assessment of missing data/drop outs. If there is no primary outcome, i) primarily use the outcome(s) measuring total volume of care/prevalence of low-value care; and ii) secondarily outcome(s) measuring intention/perception/willingness to reduce low-value care.

If there is one primary outcomes, use the proportion of missing data for judgment. If there are several primary outcomes (or if there is no primary outcome and other outcome types are used), use median values of missing outcome data for outcome category.

When judging missing data, please, remember to consider all levels of recruitment where missing data/drop outs are possible. If drop outs are highly unlikely in every level, answer "probably no" (in individually randomised trials this means only one level).

Drop outs are considered as highly unlikely when recruitment/allocation, intervention and outcome measurement happens within the same encounter.
For example, **i)** patient may be recruited, allocated, given the intervention and outcome assessed/measured within the same visit to the health care center or **ii)** physician may be recruited, allocated, intervention given and outcome measured within the same encounter (e.g. authors use survey to measure willingness to reduce use of low-value care after educational intervention and everything from allocation to measurement is done within the same encounter). In these situations, record as "probably no".

Drop outs that are included in the analysis (intention to treat principle) are not considered as missing outcome data. Sometimes authors use "intention to treat principle" in the analysis (and include

participants that did drop out but could still be followed up to measure the outcomes), but there is still drop outs with missing outcome data that they can’t include in the analysis.

4. **Other risk of bias?**
    a. **choose at least one**
        i. **Contamination –** Individually randomised trials of behaviour change interventions are usually in risk of contamination. An exception may be trials measuring effect of interventions targeted to patients and randomizing patients, when it is unlikely that intervention participants interact with the control group in a significant way.
        ii. **Selective reporting** – If trial do not report effect sizes (and group-specific data) and only mention “unsignificant results”, the study could bias the meta-analysis results.
        iii. **Other**
        iv. **No**

Methods 5. Analysis specifications

Handling of low-value care and total volume of care outcomes

We formed two categories, low-value care and total volume of care, for outcomes used in de-implementation trials in a scoping review conducted before this study. We judged total volume care (potentially including both low-value care and appropriate care) as indirect evidence and considered rating down the evidence certainty if half or more trials provided only total volume outcomes.

Outcome conversion

For continuous outcomes, if mean difference was available, we first converted them to standardized mean difference (SMD) and then to odds ratios, according to Cochrane handbook (1). If arm-specific estimates were available, we first calculated SMD (hedges' g) before converting to Odds ratios. Below are all formulas used in the conversions:

1) $SMD = MD / SD^{pooled}$
2) $SE = (CI^{upper} - CI^{lower})/2 \times t$
3) $SD = SE / \sqrt{(1/N_1 + 1/N_2)}$
4) $\ln OR = SMD \times \pi/\sqrt{3}$"

If post-intervention means were available, we used them to calculate SMD, and Odds ratios respectively. It was not possible to adjust for baseline estimates, as this would require participant-level data, and therefore only post-intervention estimates were used if effect sizes with appropriate adjustments for baseline use were not available.

We used Cochrane handbook guidance to calculate effect estimates adjusted for clustering if a trial did not report them initially (2). We used reported ICC or if that was not available, median ICC from all trials: for binary variable, practice-level ICC 0.105 and provider-level ICC 0.22; and for continuous variables ICC 0.305.

Analysis

We decided to use Hartung-Knapp adjustment in the meta-analysis as it reduces the risk of false positive findings (3). Although, it could be possible that with few positive studies with not overlapping confidence intervals, they could lead to too conservative estimates.

As large trials with continuous variables may translate to unintuitive estimates of OR, if at least three trials had continuous outcomes, we conducted a meta-analysis with the ratio of means (ROM). Using ROM was not planned in our protocol but was chosen afterwards instead of standardized mean difference (SMD) because of better interpretability. Estimates of even few percentages change in the use of low-value care from large-scale studies may lead to large effect sizes presented with SMD. If a baseline-adjusted mean difference was reported, we estimated ROM by dividing the mean difference by the post-intervention control group mean.

We decided to provide one pooled effect estimate (odds ratio) for each outcome. An alternative approach would have been to provide separate pooled effect estimates for continuous and binary outcomes (we did these as sensitivity analyses). Providing separate effect sizes for each outcome would likely decrease the interpretability and comparability of findings between different intervention types.

Control groups having education on another topic was considered similar as no intervention.

Subgroup analysis
We categorized an intervention as tailored if authors reported assessing characteristics of the study context and taking it into account, e.g. survey or focus groups to assess barriers for de-implementation.

We categorized an intervention as having a theoretical background if authors reported using theoretical information in planning the de-implementation strategy, such as referencing to theoretical literature.

For provider education trials, we categorized intervention to educational materials and educational meetings to test for the intensity of interventions. For trials combining provider education with audit and feedback, we categorized interventions to high intensity if it included either feedback given 2 times and educational meetings lasting one day (eight hours) or over in total or feedback given 3 times or more and educational meeting(s) for under one day (eight hours).

Study protocols and other published reports were additionally assessed to ensure accurate categories.

Estimating relative risk and risk differences
We estimated relative risks for receiving low-value care by using the median risk of receiving low-value care in control groups in the intervention category (4). We estimated absolute effects for receiving low-value care by using the previous estimates for relative risks and the median risk of receiving low-value care in all trials as well as for 10% and 50% baseline risks. Risk differences are reported in supplementary Table 16.

1. Deeks JJ, Higgins JPT, Altman DG (editors). Chapter 10: Analysing data and undertaking meta-analyses. In: Higgins JPT, Thomas J, Chandler J, Cumpston M, Li T, Page MJ, Welch VA (editors). *Cochrane Handbook for Systematic Reviews of Interventions* version 6.4 (updated August 2023). Cochrane, 2023. Available from www.training.cochrane.org/handbook.
2. Higgins JPT, Eldridge S, Li T (editors). Chapter 23: Including variants on randomized trials. In: Higgins JPT, Thomas J, Chandler J, Cumpston M, Li T, Page MJ, Welch VA (editors). *Cochrane Handbook for Systematic Reviews of Interventions* version 6.4 (updated August 2023). Cochrane, 2023. Available from www.training.cochrane.org/handbook.
3. IntHout, J., Ioannidis, J.P. & Borm, G.F. The Hartung-Knapp-Sidik-Jonkman method for random effects meta-analysis is straightforward and considerably outperforms the standard DerSimonian-Laird method. *BMC Med Res Methodol* **14**, 25 (2014). https://doi.org/10.1186/1471-2288-14-25
4. Schünemann HJ, Vist GE, Higgins JPT, Santesso N, Deeks JJ, Glasziou P, Akl EA, Guyatt GH. Chapter 15: Interpreting results and drawing conclusions [last updated August 2023]. In: Higgins JPT, Thomas J, Chandler J, Cumpston M, Li T, Page MJ, Welch VA (editors). *Cochrane Handbook for Systematic Reviews of Interventions* version 6.5. Cochrane, 2024. Available from www.training.cochrane.org/handbook.

Figure 1. Risk of Bias

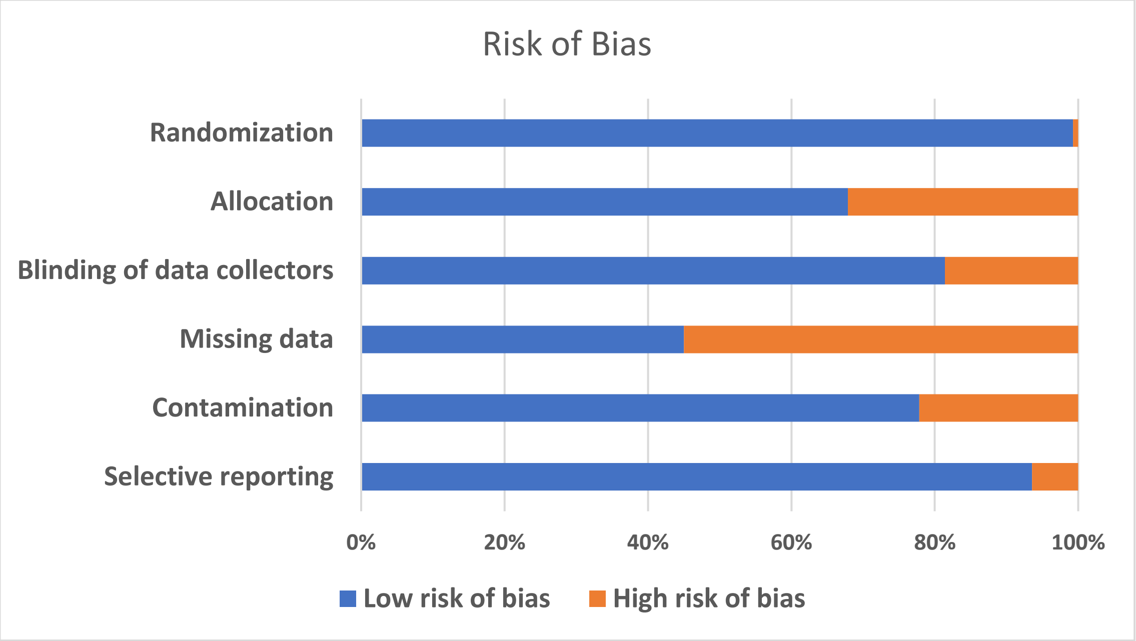

Figure 2. Provider education – Forest plot

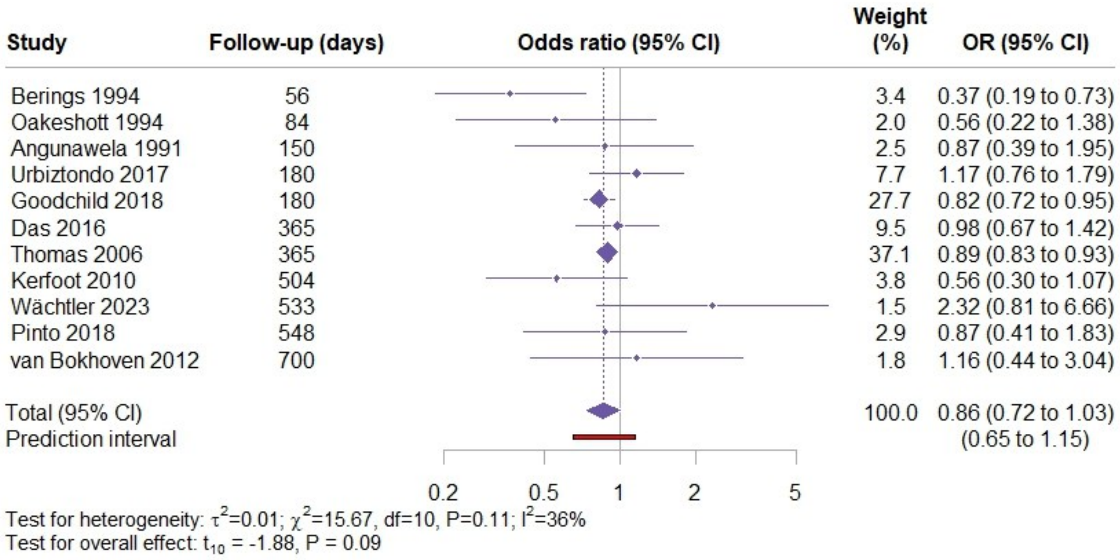

Figure 3. Subgroup analysis – Educational material vs educational meetings

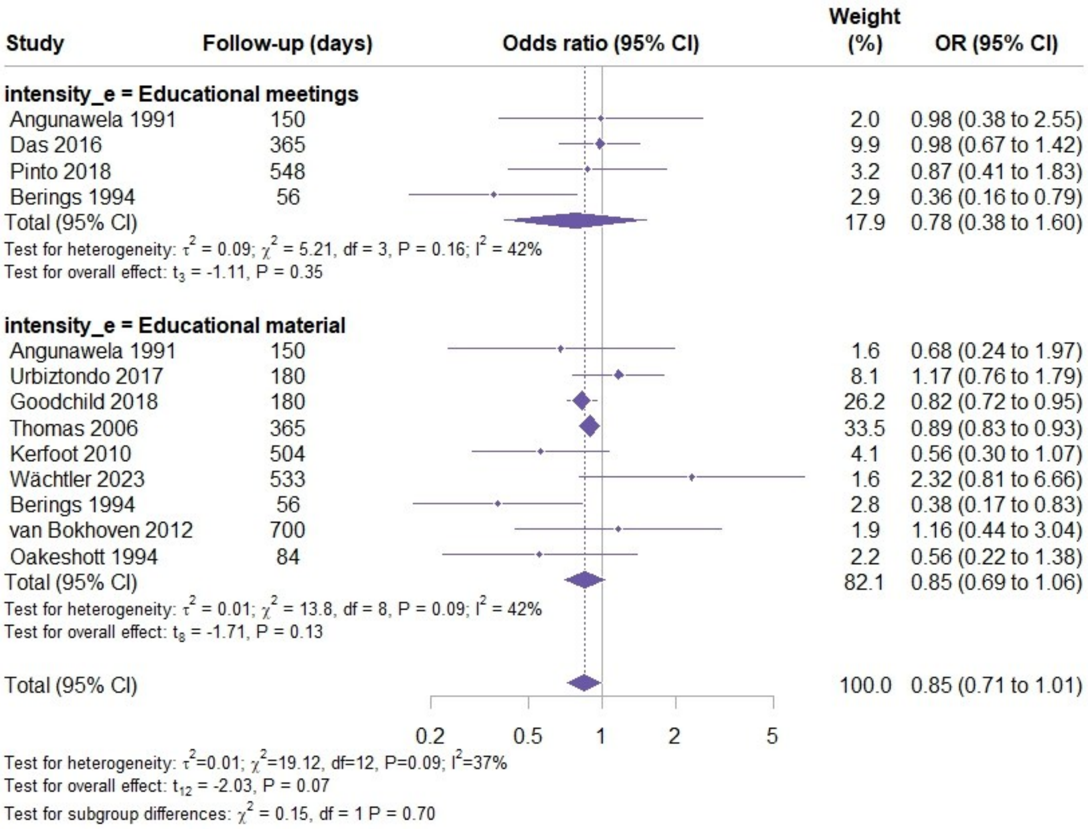

Figure 4. Subgroup analysis – tailoring vs no tailoring of the intervention

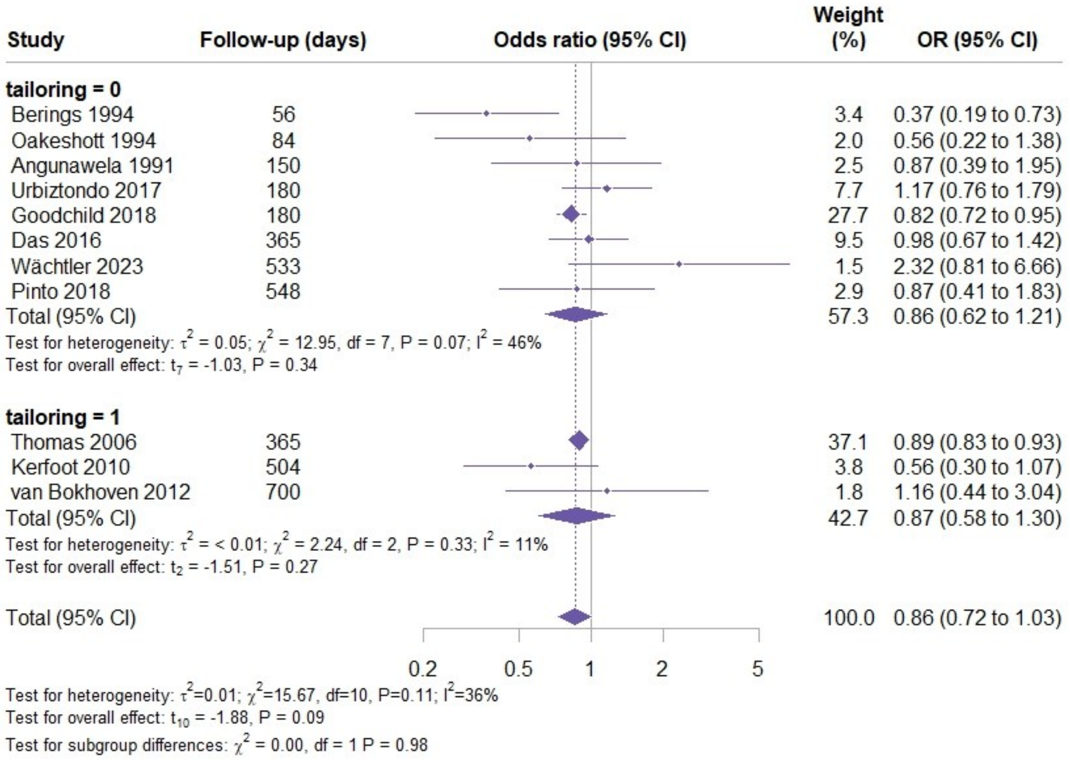

Figure 5. Subgroup analysis – with vs without theoretical background

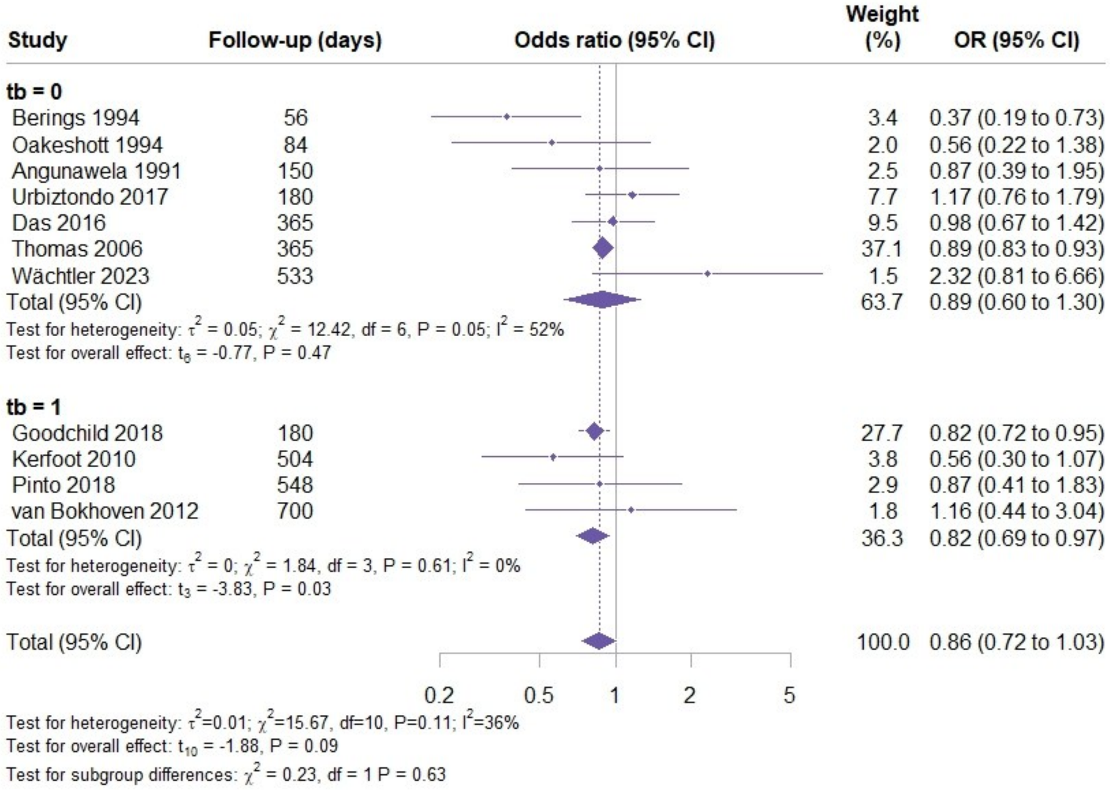

Figure 6. Sensitivity analysis – Provider education vs no intervention/educational materials

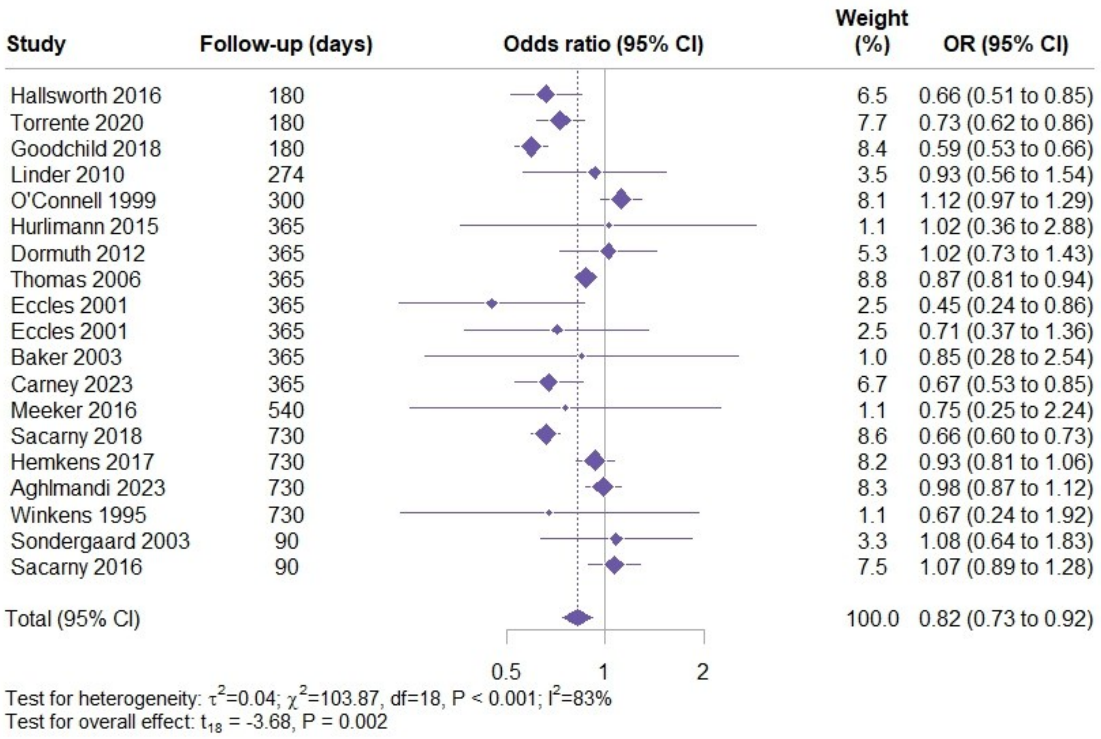

Figure 7. Sensitivity analysis – Trials with binary outcome

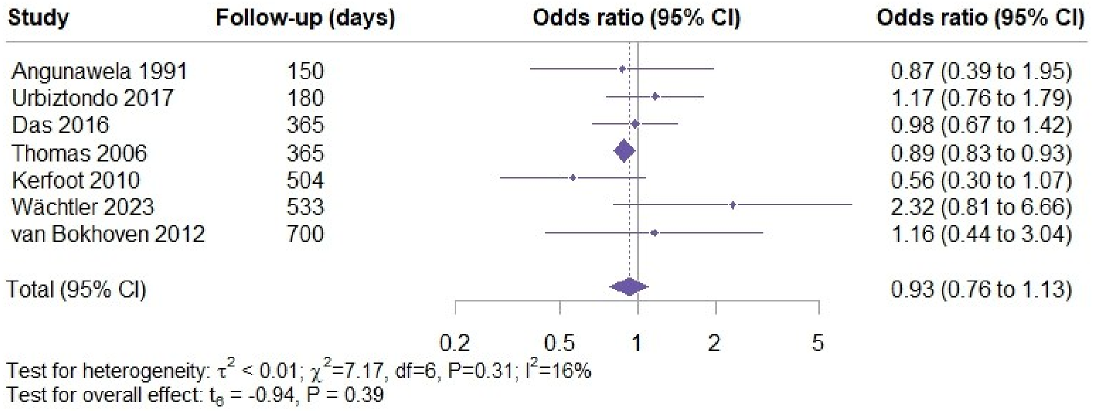

Figure 8. Sensitivity analysis – Trials with continuous outcome

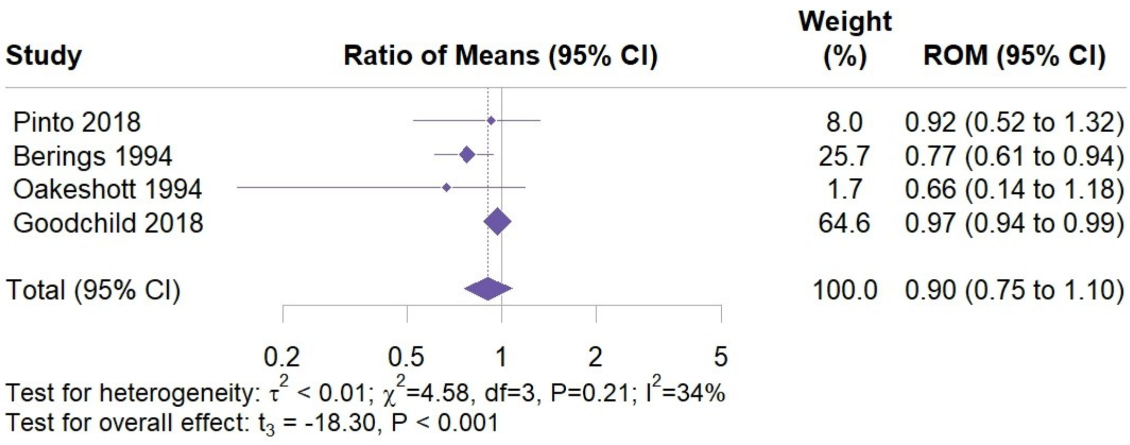

Figure 9. Audit and feedback – forest plot

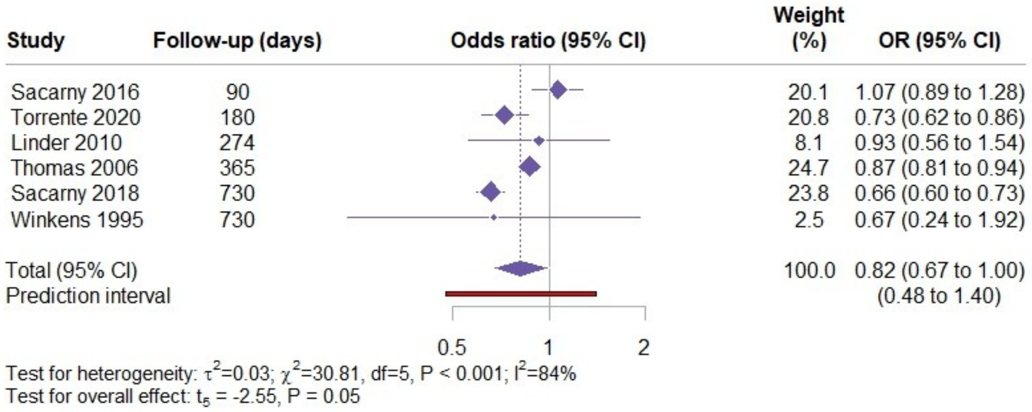

Figure 10. Sensitivity analysis – Audit and feedback with or without educational material vs no intervention or educational materials

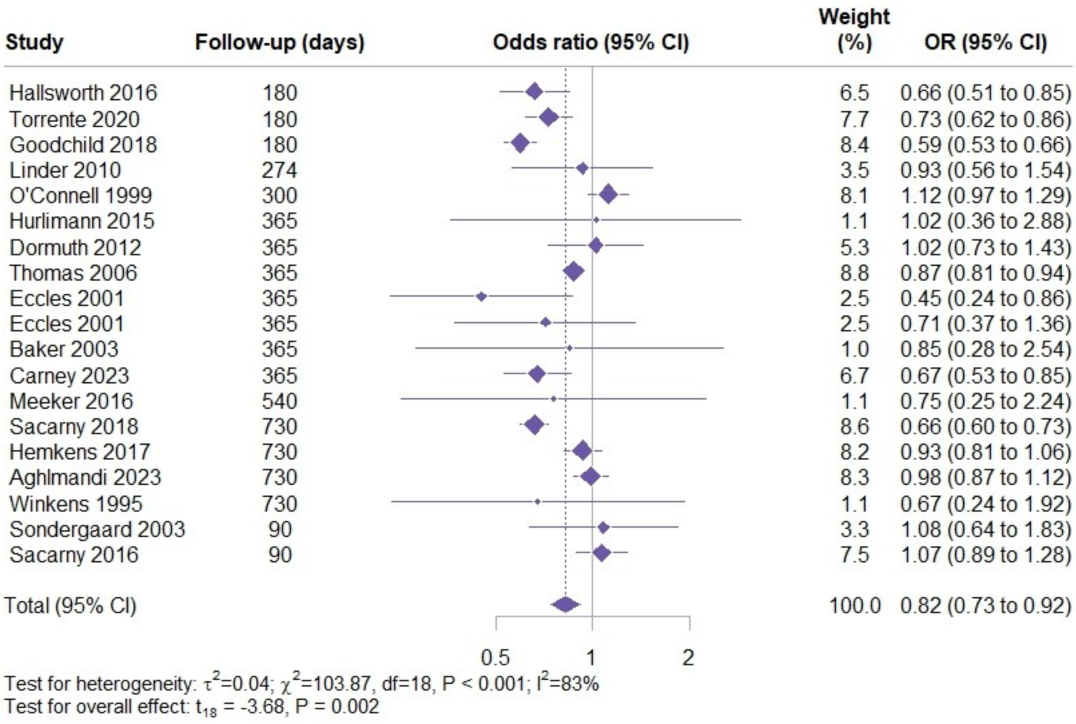

Figure 11. Sensitivity analysis – Trials with continuous outcome

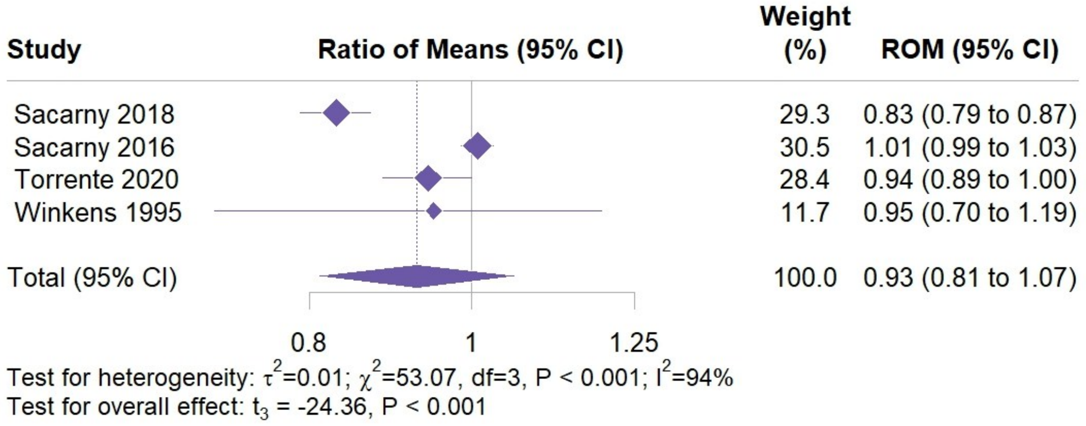

Figure 12. Provider education combined with decision support – forest plot

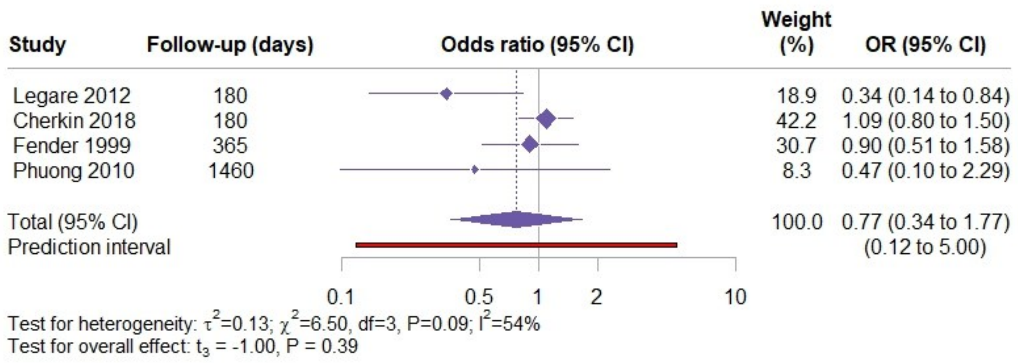

Figure 13. Sensitivity analysis – Provider education combined with decision support versus no intervention or educational materials

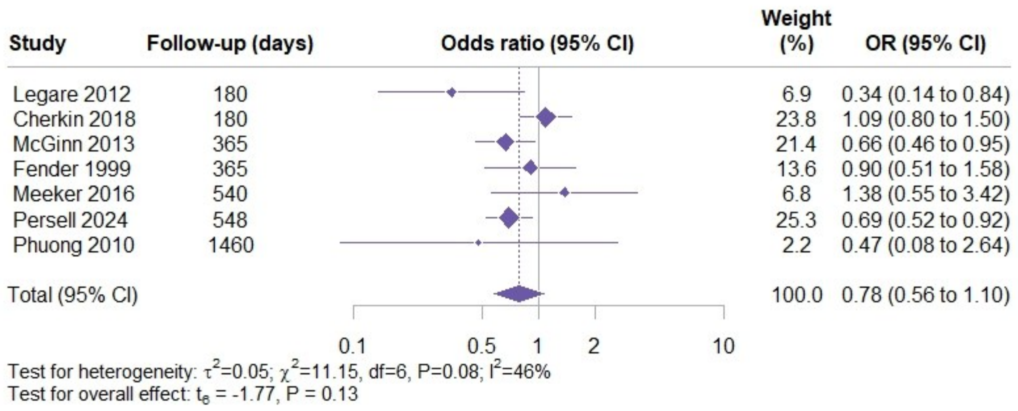

Figure 14. Audit and feedback combined with provider education – forest plot

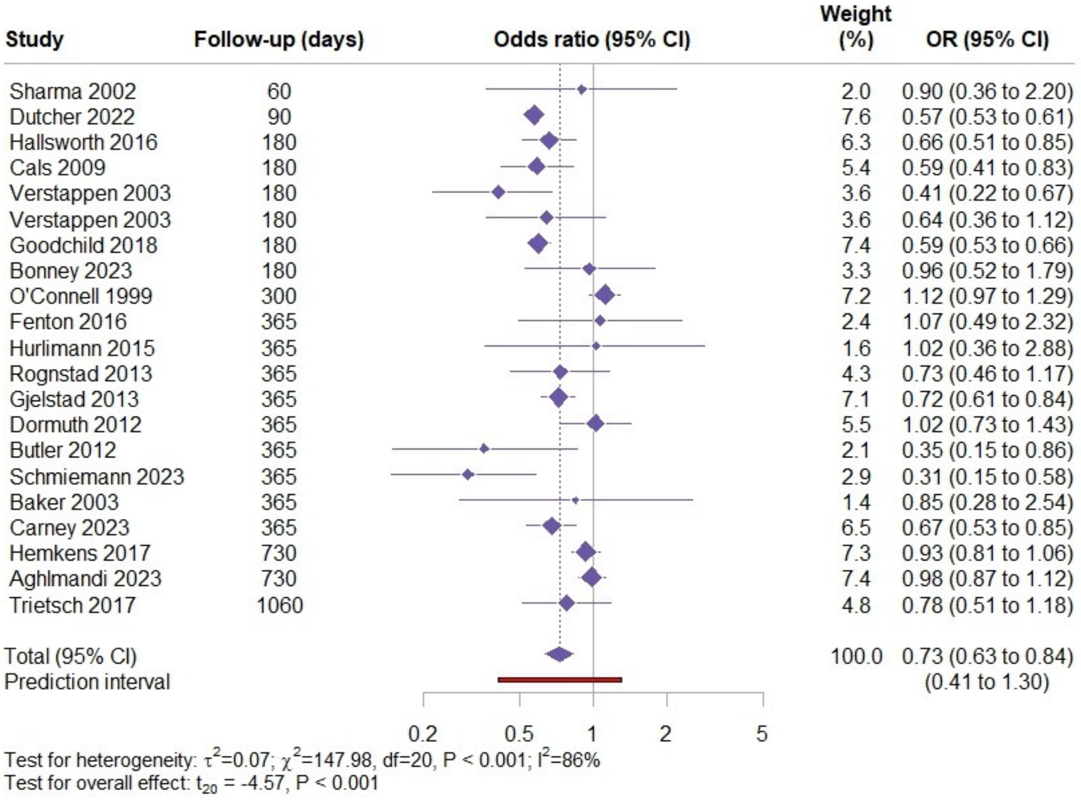

*One trial (Verstappen 2003) had two intervention arms receive education on different low-value care topics and was included in the meta-analysis separately

Figure 15. Sensitivity analysis – Audit and feedback combined with provider education vs no intervention or educational materials

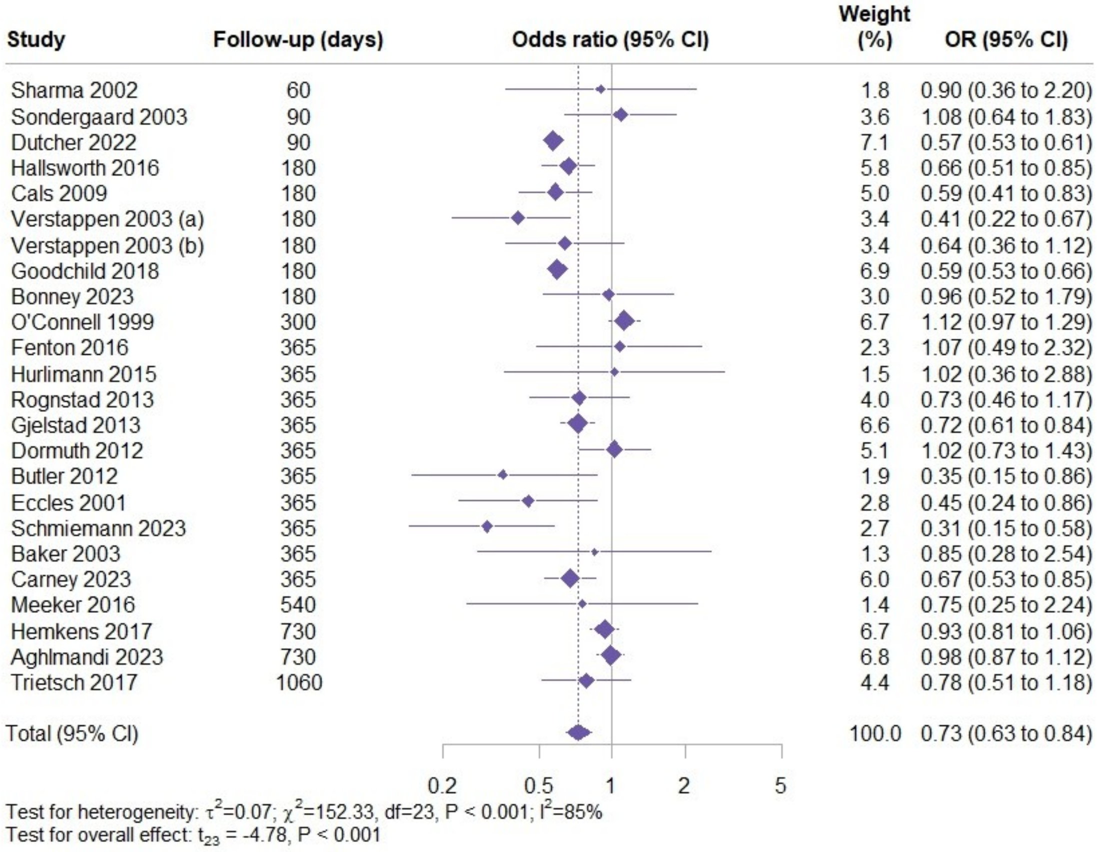

Figure 16. Subgroup analysis – low vs high intensity

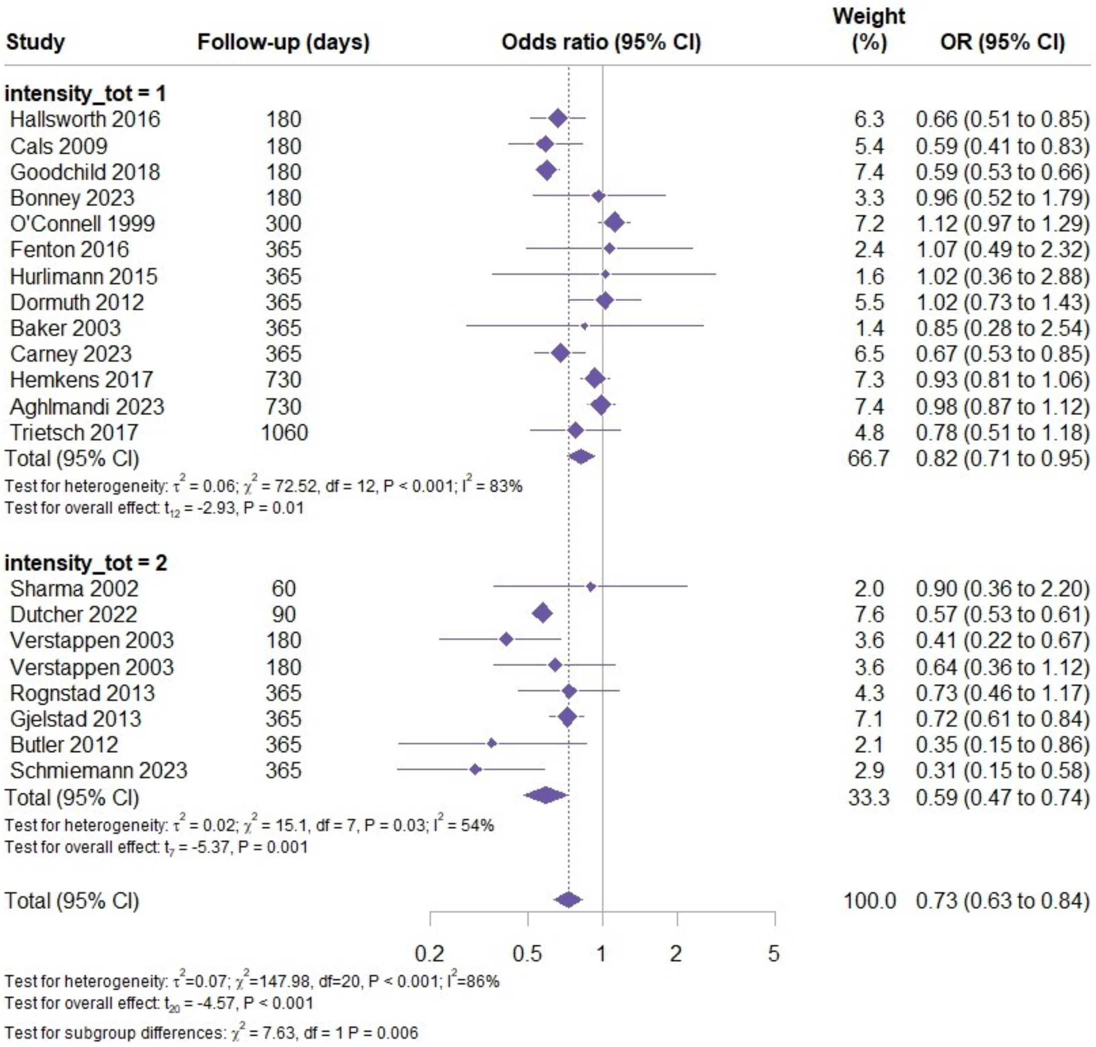

Figure 17. Subgroup analysis – tailoring vs no tailoring of the intervention

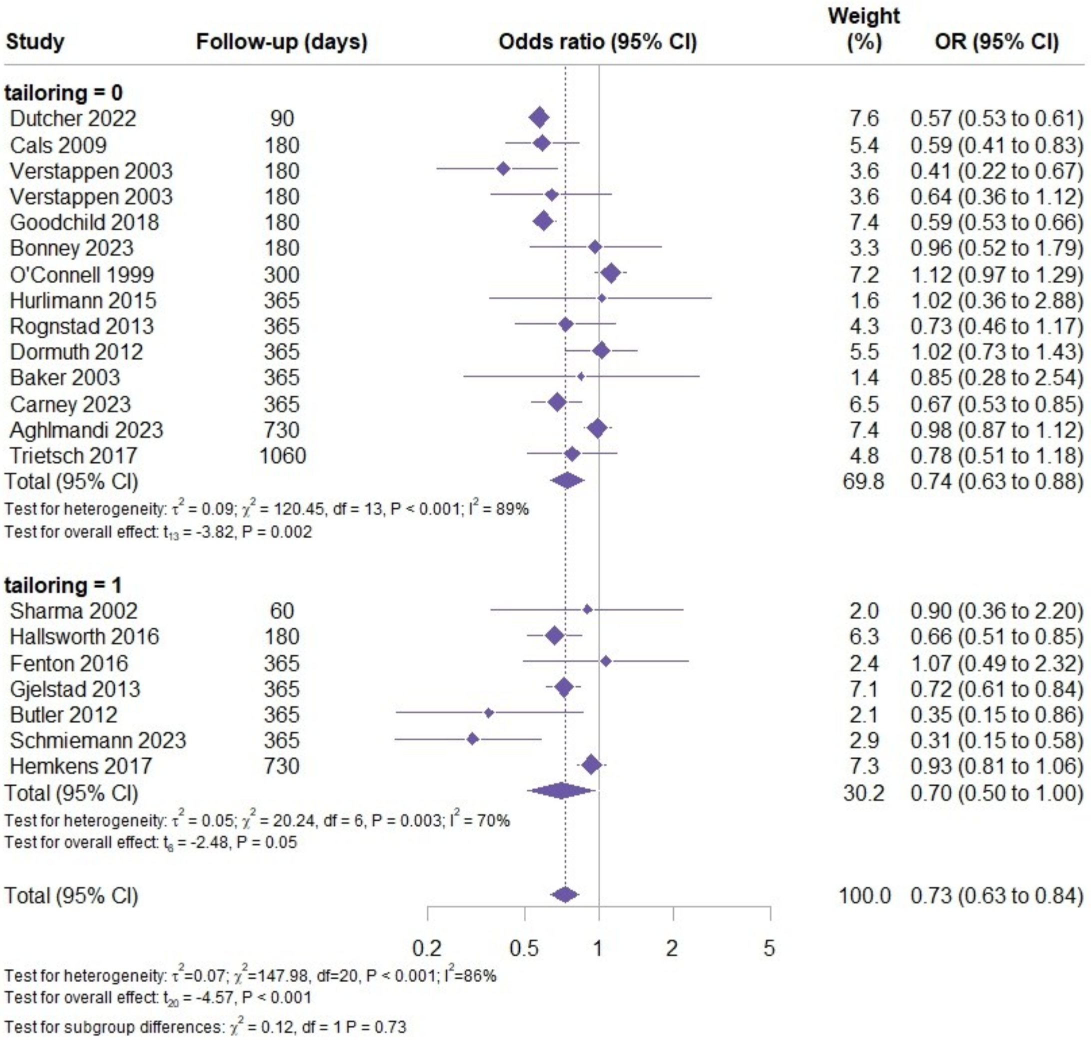

Figure 18. Subgroup analysis – with vs without theoretical background

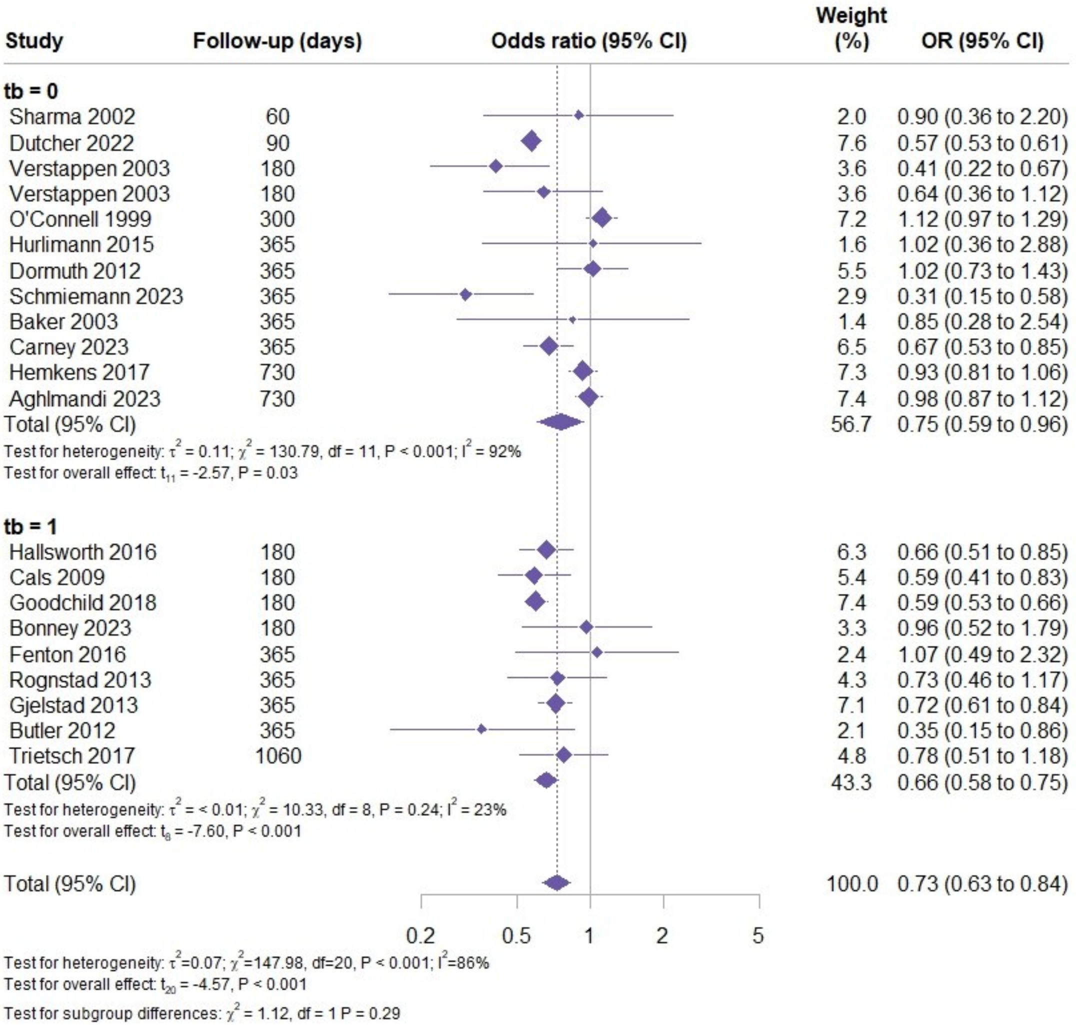

Figure 19. Sensitivity analysis – trials with binary outcome

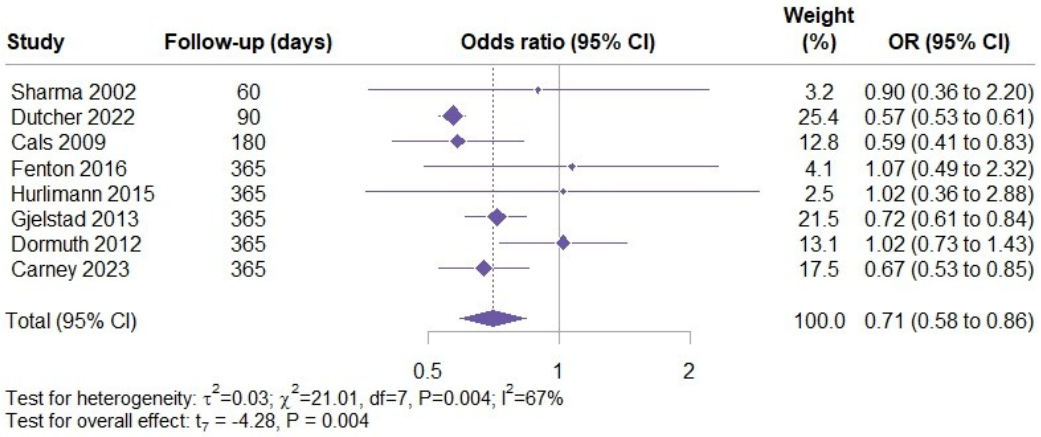

Figure 20. Sensitivity analysis – trials with continuous outcome

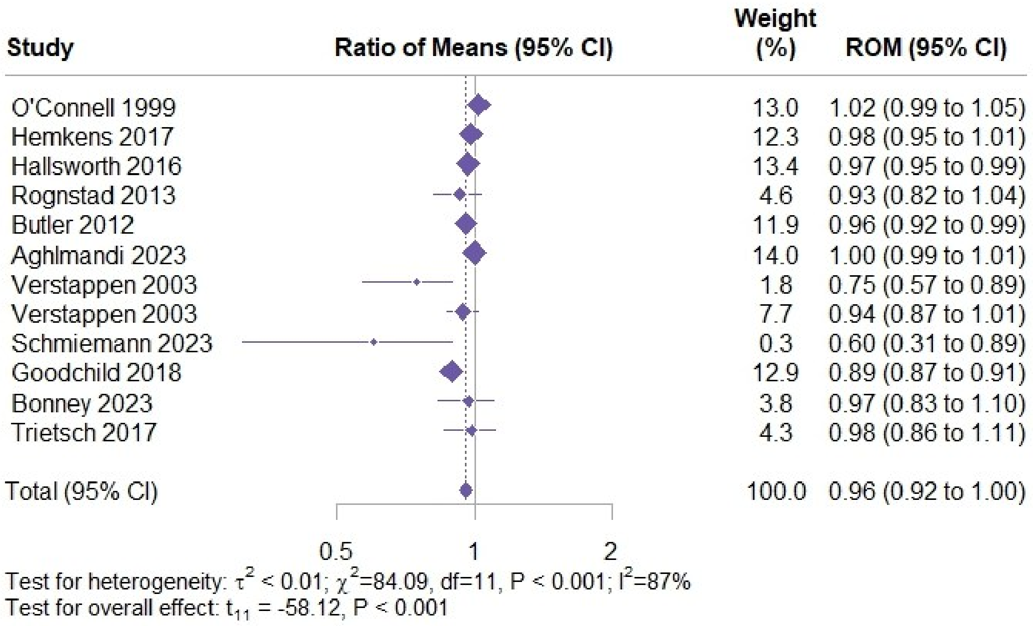

Figure 21. Sensitivity analysis – randomization unit

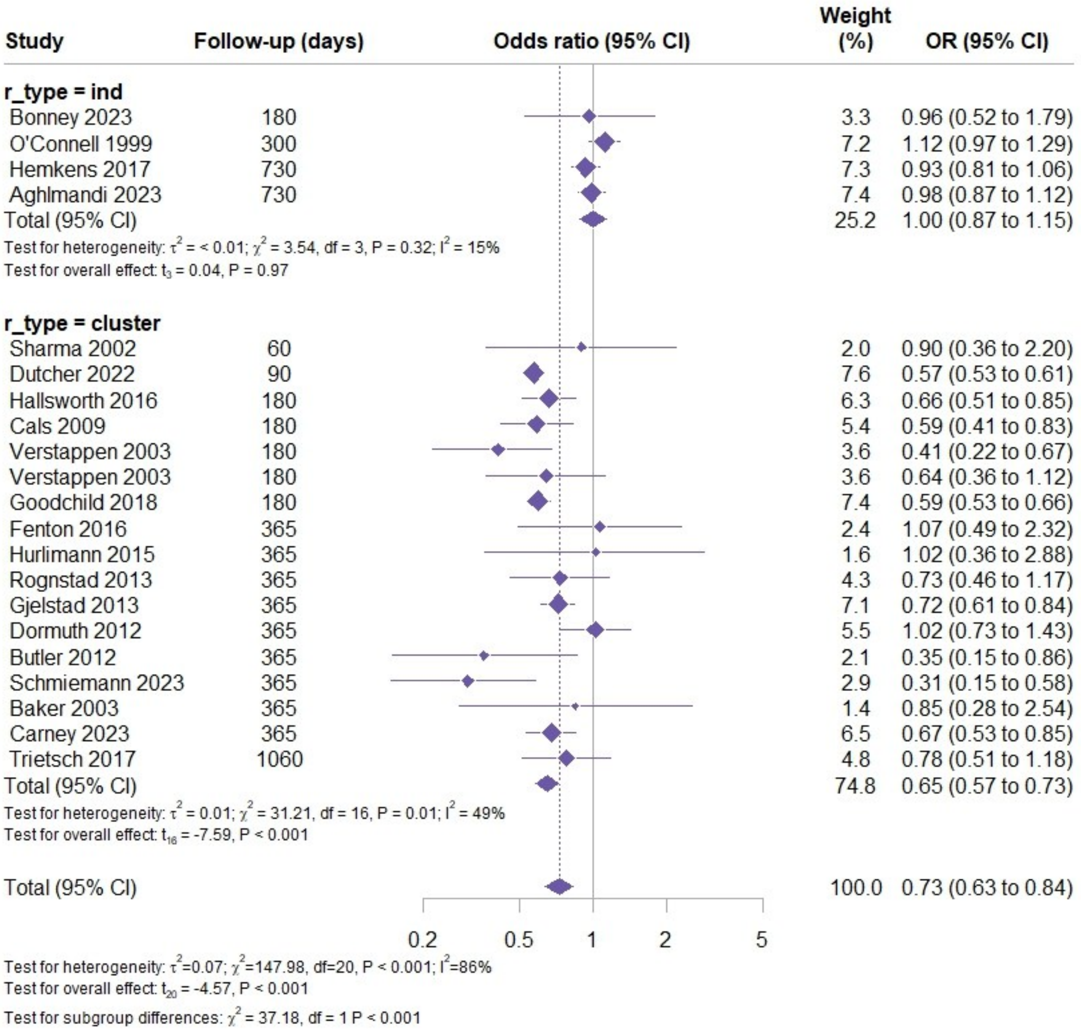

Figure 22. Patient education – forest plot

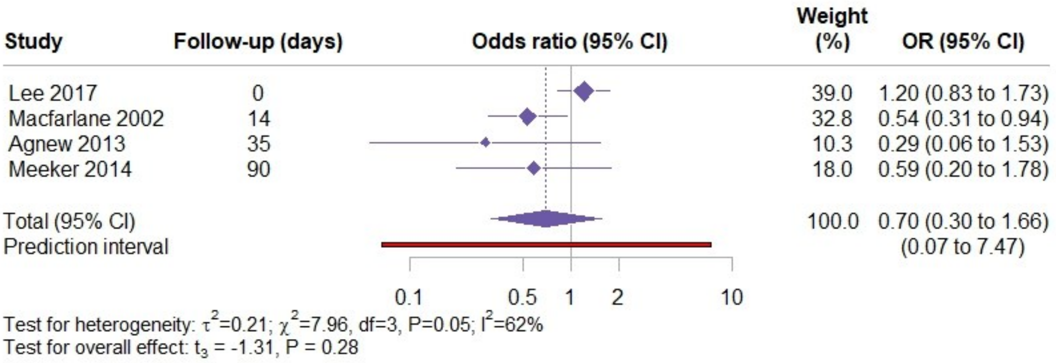

Figure 23. Sensitivity analysis – without one trial including control of education on viral infections

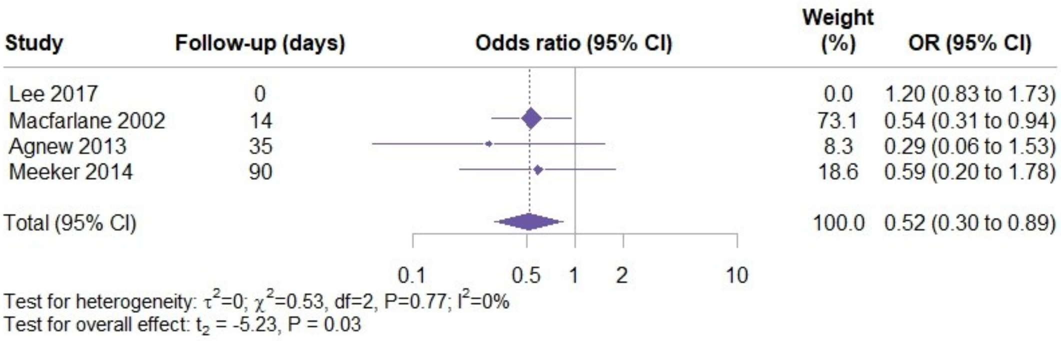

Figure 24. Patient education and provider education combined – forest plot

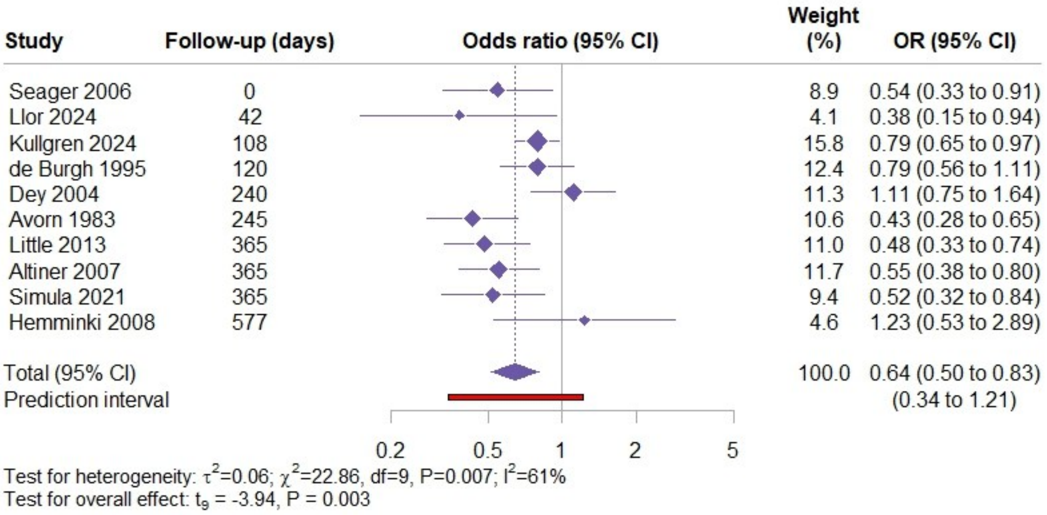

Figure 25. Sensitivity analysis – trials with binary outcome

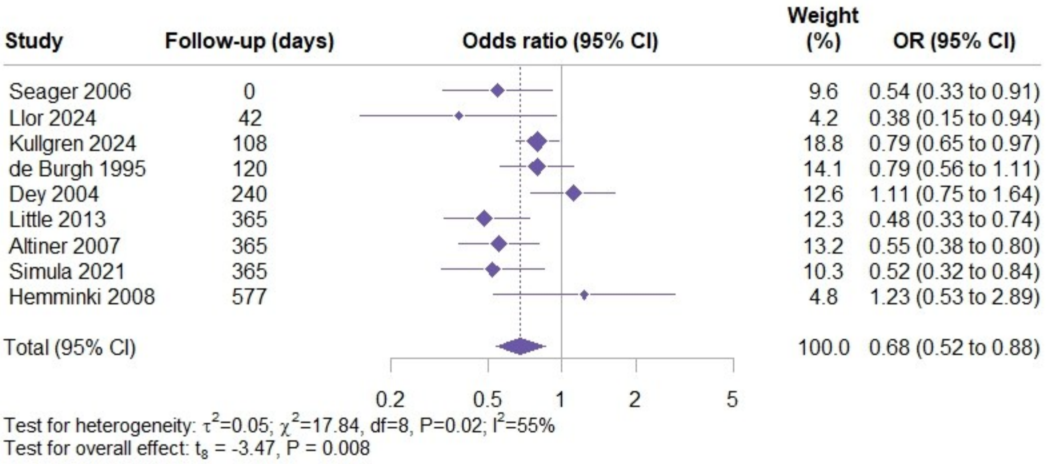

Figure 26. Subgroup analysis – tailoring versus no tailoring

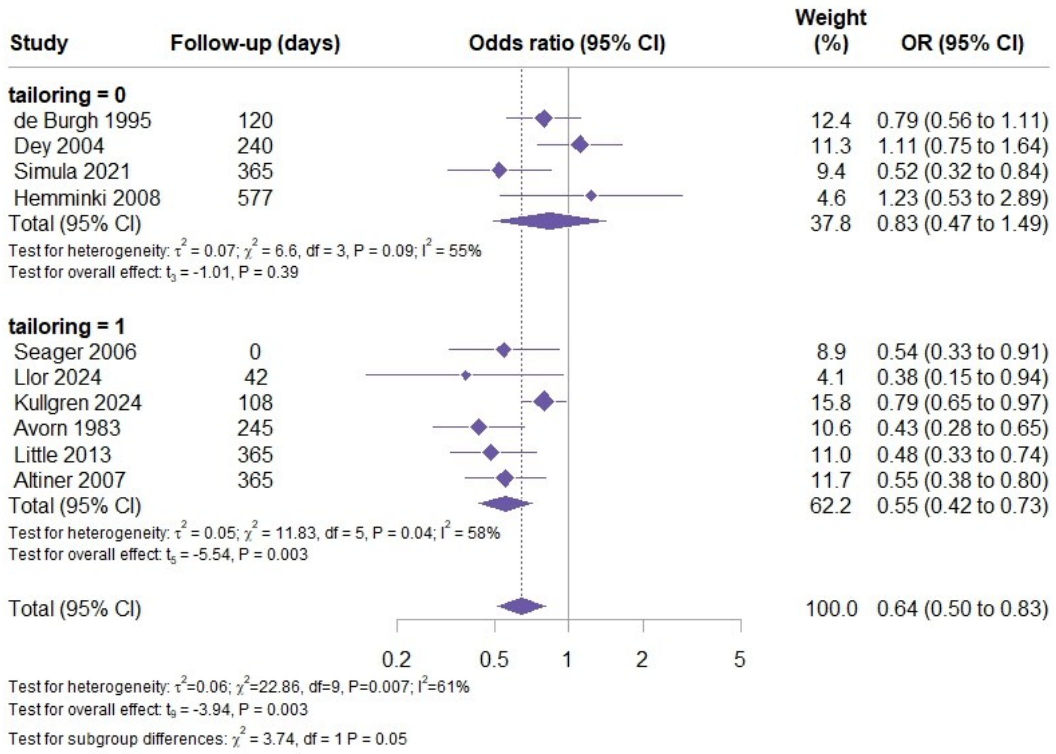

Figure 27. Subgroup analysis – theoretical background versus no theoretical background

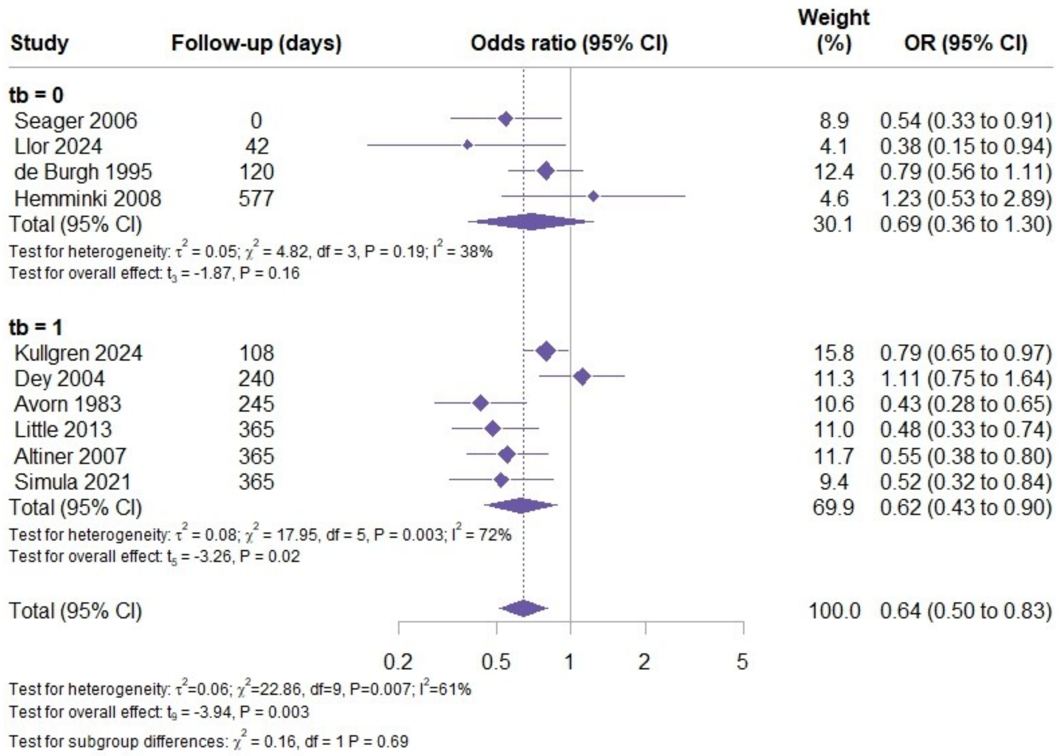

Figure 28. Patient education combined with provider education and decision support

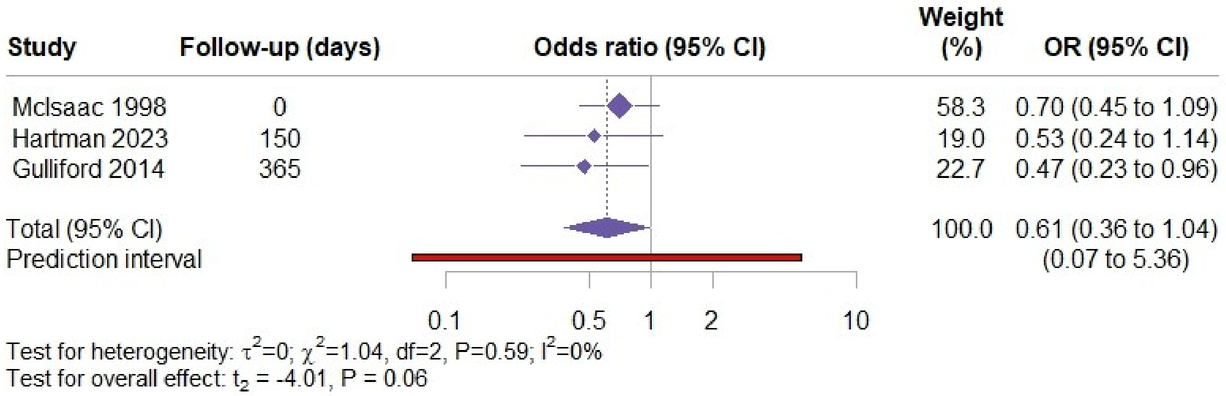

Figure 29. Patient education combined with audit and feedback, and provider education

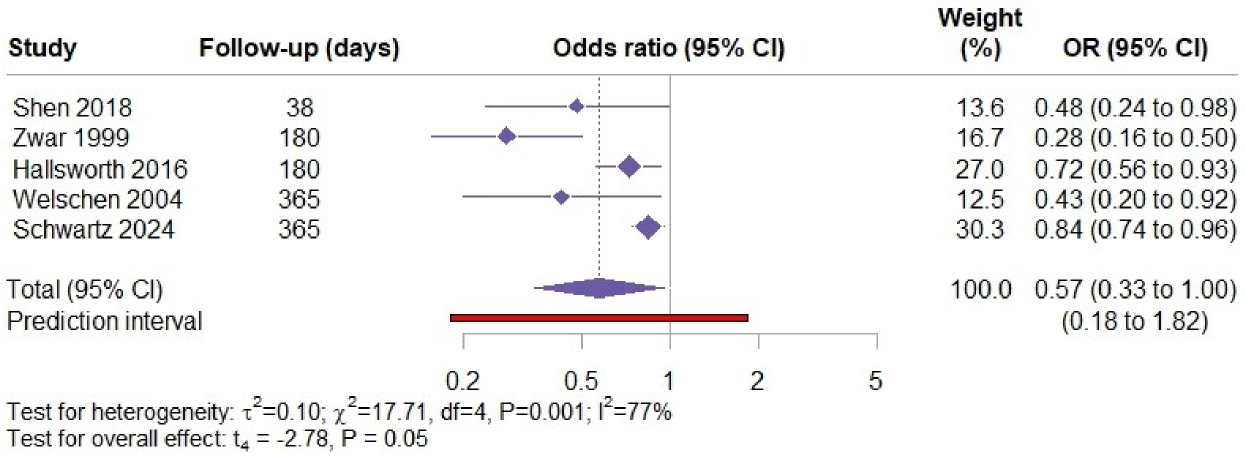

Figure 30. Sensitivity analysis – trials with continuous outcome

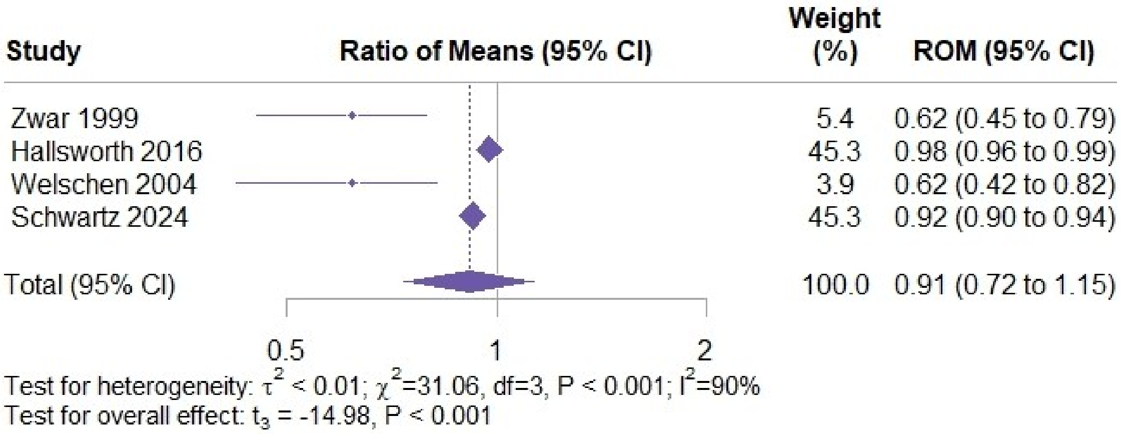

Figure 31. Sensitivity analyses on continuous and binary outcomes as well as trials with no imbalance

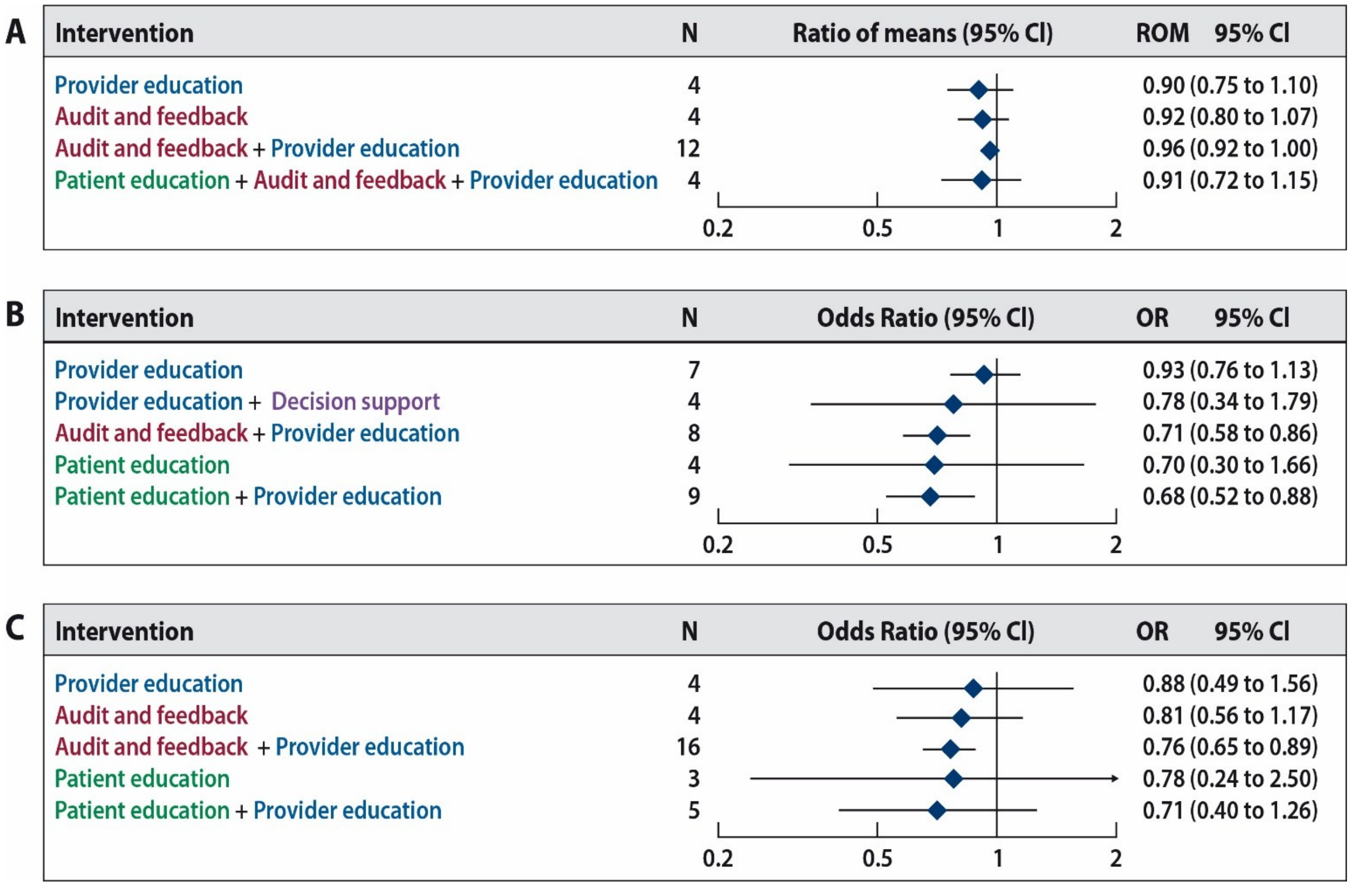

A: Sensitivity analysis on trials continuous outcomes; B: Sensitivity analysis on trials with binary outcomes; C: sensitivity analysis on trials with no baseline imbalance

Table 1. Study characteristics – Other interventions

Decision aid																
Author	Number of participants	follow-up (days)	Follow-up mean/events	OR (95% CI)	Relative reduction (ROM or RR)	Target	Intervention	Tailoring	TB	Risk of bias						BI
Worrall 2007	37 providers, 533 patients	short	I: 94/170<br>B: 82/141	0.89 (0.35-2.24)	0.95 (0.57-1.30)	Antibiotics (TV)	1) Decision support, giving specific criteria for starting antibiotics in sore throat	no	no	+	–	+	+	–	+	yes
Martens 2007	22 practices, 50 physicians	365	two outcomes<br>I: 28.2 (37.7)<br>1.1 (11.0)<br>C: 39.7 (51.4)<br>2.2 (14.9)	0.75 (0.20-1.76)	-	Antibiotics (TV)	1) EHR decision support - automated system triggering with specific criteria - provided info on alternative treatment paths for several clinical condition including respiratory tract infections, asthma and COPD	no	no	+	+	+	–	+	+	yes
**Laboratory system changes**																
Author	Number of participants	follow-up	Follow-up mean/events	OR (95% CI)	Relative reduction (ROM or RR)	Target	Intervention	Tailoring	TB	Risk of bias						BI
Chien 2017	1258 providers	365	I1: 0.3 (0.5)<br>I2: 0.3 (0.8)<br>C: 0.3 (0.6)	-	1.00 (0.76-1.24)	Laboratory tests (LVC)	1) Median prices of lab tests in the test-ordering screen of their electronic health record<br>2) Paper and electronic memos introduced the intent of the price information	no	no	+	+	+	–	+	+	no
Martins 2017	14 practices	240	I: 9.75 (1.21)<br>C: 11.8 (0.88)	-	0.48 (0.35-0.60)	Laboratory tests (LVC)	1) Removing of laboratory tests from basic EHR menu<br>2) Traffic lights on tests in the test ordering menu, based on recommendations + short educational info when test was clicked in the menu	no	no	+	+	+	–	+	+	yes
Tierney 1990	111 physicians	182	I: 1.56 (0.72)<br>C: 1.82 (0.90)	-	0.86 (0.69-1.03)	Laboratory tests (TV)	1) Price information on test ordering screens	no	no	+	+	+	–	–	+	no

Patient education combined decision aids

Author	Number of participants	follow-up	Follow-up mean/events	OR (95% CI)	Relative reduction (ROM or RR)	Target	Intervention	Tailoring	TB	Risk of bias						BI
Linder 2009	27 practices, 14454	210	I: 2601/8218 C: 2108/6236	0.9 (0.6-1.4)	0.93 (0.69-1.23)	Antibiotics (LVC)	1) EHR integrated decision support on respiratory tract infections, launched by physician<br>2) Educational material for patient, possible to print from the decision support	no	no	+	+	+	+	+	+	no
Jenkins 2013	8 practices, 37241 patients	365	I: 2991/7897 C: 1569/4052	0.96 (0.37-2.54)	0.98 (0.49-1.59)	Antibiotics (TV)	1) Paper and web-based decision support for treatment choices on common outpatient infections<br>2) Local opinion leaders to advocate for use of decision supports<br>3) Patient education materials	no	no	+	−	+	−	+	+	yes

Provider and patient education combined decision aids and audit and feedback

Author	Number of participants	follow-up	Follow-up mean/events	OR (95% CI)	Relative reduction (ROM or RR)	Target	Intervention	Tailoring	TB	Risk of bias						BI
Gulliford 2019	79 practices	365	I: 98.7 (NR) C: 107.6 (NR)	-	0.88 (0.78-0.99)	Antibiotics (TV)	1) One time webinar<br>2) Audit and feedback, monthly emailed individual reports with peer comparison + links to decision supports<br>3) Educational leaflets to patients<br>4) Decision support activated when specific diagnostic codes were put into EHR<br>5) Local practice champions promoted the intervention	yes	yes	+	−	+	−	+	+	no
Gonzales 2013	33 practices, 2968 patients	182	0,743157895	0.63 (0.36-1.08)	0.87 (0.69-1.02)	Antibiotics (LVC)	1) Educational meetings<br>2) Audit and feedback (only practice level data)<br>3) Educational brochures to patients<br>4) Clinical algorithm displayed as posters in examination rooms and tool integrated in EHR or on paper	yes	yes	+	−	+	−	+	+	no

Other combinations																
Author	Number of participants	follow-up	Control risk	OR (95% CI)	Relative reduction (ROM or RR)	Target	Intervention	Tailoring	TB	Risk of bias						BI
Flottorp 2002 (a)*	113 practices, 13649 patients	240	two outcomes<br>I: 2202/5031<br>C: 1552/3135<br>I: 2111/5031<br>C: 1246/3135	0.81 (0.62-1.07)	0.89 (0.75-1.04)	Laboratory tests + antibiotics (TV)	1) Educational material for patients<br>2) EHR integrated decision aid + reminders<br>3) Increase in fee of telephone consultations<br>4) Interactive educational meetings for GPs and nurses	yes	no	+	+	+	−	+	+	no
Flottorp 2002 (b)*	113 practices, 13649 patients	240	I: 1256/2522<br>C: 1629/2961	0.81 (0.64-1.03)	0.91 (0.80-1.01)	Laboratory tests (TV)	1) Educational material for patients<br>2) EHR integrated decision aid + reminders<br>3) Increase in fee of telephone consultations<br>4) Interactive educational meetings for GPs and nurses	yes	no	+	+	+	−	+	+	no
Hamilton 2007	1610 patients	42	I: 457/811<br>C: 418/799	1.26 (1.07-1.48)	1.11 (1.03-1.19)	other drugs (TV)	1) A self-completed agenda form (SCAF) by patients asking five questions, including whether the patient considered they should receive a prescription.	yes	no	+	+	+	−	−	+	yes
Raebel 2007	59680 patients	365	I: 327/29840<br>C: 447/29840	0.73 (0.63-0.84)	0.73 (0.63-0.84)	other drugs (TV)	1) Local consensus and guidelines on inappropriate medications were formed.<br>2) When an intervention group patient was newly prescribed a potentially inappropriate medication, the pharmacist was notified via a medication alert generated from PIMS. The medication alert functioned by not allowing the prescription label to print until the pharmacist had actively intervened to determine whether the prescription should be dispensed.	no	no	+	+	+	+	−	+	no

Avent 2024	27 practices, 110 physicians	182	I: 52.5 (22.4)<br>C: 55.5 (23,7)	0.79 (0.39-1.57)	0.95 (0.79-1.10)	Antibiotics (TV)	Participants could choose preferred interventions from following options:<br>1) Commitment posters and endorsement to include GPs' picture along<br>2) Patient information leaflet<br>3) Online communication training package<br>4) Delayed prescription possibility with stickers to put on prescriptions<br>5) Decision aids to promote shared decision making<br>6) Access to CRP tests and 50 tests per months free of charge + training on using	yes	no	+	+	+	+	+	+	no
Bhatia 2017	196 physicians	502	I: 8.8% (9.5)<br>C: 10.1% (8.1)	0.75 (0.57-0.99)	-	Imaging	1) Video-lecture, describes the AUC for echocardiography and clinical scenarios for which outpatient TTEs are ordered<br>2) Access to the American Society of Echocardiography mobile application<br>3) Individualized monthly feedback reports sent through secure electronic communication summarizing TTE ordering behavior. The feedback reports including total volume and number of inappropriate TTEs ordered	no	no	+	+	+	+	+	+	no

TB = theoretical background, BI = baseline imbalances, TV=total volume outcome, LVC=low-value care outcome
*two study arms acted as control for each others – first aimed to reduce antibiotic and lab use in sore throat, second aimed to decrease use of urinary tract infection lab tests

Table 2. Study characteristics - trials with other control groups

Author	Number of participants	Follow-up (days)	Follow-up mean/events	OR (95% CI)	Relative reduction (ROM or RR)	Target	Intervention	Control	Tailor	TB	Risk of bias						BI
Nejad 2016	485 providers	180	I: 101.90 (179.5)<br>C: 104.38 (234.8)	-	0.98 (0.62-1.34)	other drugs (TV)	1) Traditional paper letters with intensified provision and modified content	Short text message feedback	no	no	+	+	+	−	−	+	no
Cundill 2015	36 practices, 29223 patients	395	I1: 250/10118<br>I2: 184/10163<br>C: 749/8942	0.28 (0.07-1.05)<br>0.20 (0.05-0.90)	0.29 (0.08-1.04)<br>0.22 (0.05-0.90)	other drugs (LVC)	2 intervention arms, first got first two components and second got all:<br>1) Small group workshops<br>2) Feedback on rapid diagnostic test use and motivational educational mobile-phone text messages (SMS)<br>3) Patient leaflets and clinic posters	2-days educational seminar (also given to the intervention arms)	yes	yes	+	+	−	−	+	+	yes
Briel 2006	30 providers, 552 patients	150	I: 35/259<br>C: 46/293	0.86 (0.40-1.93)	0.88 (0.44-1.68)	Antibiotics (TV)	1) Training on patient centered communication (6 hours),<br>2) Guideline distribution<br>3) Educational seminar (2 hours) on guidelines	Got guidelines and seminar on guidelines (same as intervention group)	no	yes	+	−	+	−	+	+	no
Samore 2005	12 communities	730	I: 75.3 (27.9)<br>C: 85.2 (27.9)	-	0.88 (0.49-1.28)	Antibiotics (TV)	1) Community intervention - introductory meetings with community leaders, news releases, distribution of educational materials at pharmacies and physician offices, and a mailing to parents of children.<br>2) CDSS intervention - decision support tools on paper or in handheld digital assistant. The decision support tools were introduced to primary care clinicians through educational lectures, small group meetings, and one-on-one interactions.	Community intervention alone	no	yes	+	+	+	−	+	+	no

McIsaac 2002	97 providers, 453 patients	short	-	0.76 (0.42-1.40)	-	Antibiotics (LVC)	1) Decision support - patient chart stickers in EHR that prompted to use a form to calculate a score based on clinical findings and provided management recommendations linked to score totals<br>2) Educational materials on the clinical score, pocket card summary and clinical assessment forms	no stickers in the EHR, otherwise received the same material	no	no	+	−	−	−	+	+	yes
López 2005	27 practices, 154 providers	274	I: 1.34 (0.61)<br>C: 1.62 (0.78)	-	0.84 (0.77-0.92)	other drugs (TV)	1) Educational training sessions on a critical reading of the studies available on recently marketed drugs, four 45min sessions<br>2) Audit and feedback four times<br>3) Educational materials on therapeutic novelties	feedback and educational materials	yes	no	+	+	+	+	+	+	yes
Verstappen 2004	26 practices, 174 providers	180	I: 422 (235)<br>C: 535 (309)		0.90 (0.84-0.97)	laboratory tests (TV)	1) personalized graphic feedback, including a comparison of each physician’s own data with those of colleagues<br>2) dissemination of and education on national, evidence-based guidelines<br>3) continuous quality improvement meetings in small groups, including attempt to form consensus on test ordering	personal graphic feedback on prescribing	no	no	+	+	+	−	+	+	yes
Curtis 2021	1401 practices	365	-	-	0.97 (0.94-1.01)	Antibiotics (LVC)	1) Short written feedback three times highlighting the practice’s high antibiotic prescribing compared with other practices<br>2) Second feedback included a link to prior evidence of feedback prompting change in antibiotic prescribing and an invitation to contact the authors and third wave a tailored chart of potential cost savings* and more information about other data available at OpenPrescribing.net	access to the audit and feedback as usual, no messages	no	no	+	+	+	+	+	+	no

Mann 2020	33 practices, 33314 patients	981	I: 3256/13982<br>C: 5292/19332	-	0.90 (0.7-1.2)	Antibiotics (LVC)	1) Clinical prediction rules tool which triggers in EHR system with the right indicators. Shows risk score for pharyngitis and pneumonia.<br>2) academic detailing, same as control	All participants received 45min academic detailing session and materials distributed (guidelines, clinical prediction rules, online links).	yes	yes	+	+	+	−	+	+	no
Poss-Doering 2021	14 practices, 21949 patients	630	I1: 1272/6730<br>I2: 1198/5076<br>C: 2039/10143	0.86 (0.66-1,13)<br>1.02 (0.79-1.33)	-	Antibiotics (TV)	1) E-learning for physicians<br>2) Quality circles with data-based feedback for physicians<br>3) Information campaigns for the public<br>4) Information campaigns for patients in respective practices<br>5) Performance-based additional reimbursement (P4P)<br>6) E-learning for medical assistants<br>7) Quality circles with data-based feedback for medical assistants, audit and feedback<br>8) Information material available on tablet computers<br>9) Computerised decision support system (CDSS)<br>10) Quality circles in local multidisciplinary groups<br>all groups had components 1-5, group ii had also 6-8, and group iii had 1-5 + 9-10	components 1-5	no	no	+	−	+	+	+	+	no
van Vugt 2021	26 practices, 195394 patients	365	-	two outcomes:<br>0.88 (0.83-0.92)<br>0.96 (0.91-1.02)	-	laboratory tests (TV)	1) training sessions on indications for d vitamin and b12 vitamin testing and communication<br>2) audit and feedback, peer comparison<br>3) educational material for patients - video in waiting rooms and leaflets	components 1-2	no	no	+	+	+	+	+	+	yes

Du Yan 2021	45 providers, 5354 patients	210	I: 757/2357<br>C: 1057/2997	0.87 (0.48-1.57)	-	Antibiotics (LVC)	1) 1-hour Educational meeting<br>2) Online dashboard with antibiotic prescription rates	Educational meeting and material	no	yes	+	−	+	+	+	+	yes
Feldmeier 2023	114 practices, 61390 patients	548	I: 2133/13658<br>C: 2058/15781	0.956 (0.91-1.00)	0.96 (0.92-1.00)	Antibiotics	1) Public awarness campaign including written info for GPs, posters in the waiting rooms, magazines, and push toys for kids<br>2) Audit and feedback - two times practice specific feedback on antibiotic prescribing<br>3) Educational outreach visits including discussions about the prescribing feedback<br>4) Patient information leaflets + information on tablets	The first component (public awarness campaign)	no	no	+	−	+	+	+	+	yes

TB = theoretical background, BI = baseline imbalances, TV=total volume outcome, LVC=low-value care outcome

Table 3. Study characteristics - Provider education

Study	Number of participants	Follow-up (days)	Follow-up mean/events	OR (95% CI)	Relative reduction (ROM or RR)	Target (TV/LVC))	Intervention	Tailoring	TB	Risk of bias						BI
Angunawela 1991*	15 practices, 2229 patients	150	Two intervention groups:<br>I1: 116/480<br>I2: 274/870<br>C: 280/879	0.87 (0.39-1.95)	0.90 (0.48-1.50)	Antibiotics (TV)	1) Educational material including guidelines for rational prescribing, mailed once.<br>2) One 3-hour educational seminar including educational material distribution, delivered by 4 educators.<br>I1 got materials and I2 got both components	no	no	+	+	+	+	+	+	Yes
Urbiztondo 2017	73 practices, 705 patients	180	I: 183/327<br>C: 197/378	1.17 (0.83-1.65)	1.07 (0.88-1.26)	Antibiotics (LVC)	1) Online clinical cases with multiple choice answers, feedback and links to relevant literature, emailed once.	no	no	+	+	−	−	+	+	Yes
Das 2016	304 practices	365	I: 0.701 (0.47)<br>C: 0.707 (0.45)	0.98 (0.67-1.42)	0.99 (0.88-1.10)	Antibiotics + other drugs (LVC)	1) 150-hour training program to improve the qulity of curative care in rural setting.	no	no	+	+	+	+	−	+	No
van Bokhoven 2012	35 practices, 269 patients	700	I: 27/174<br>C: 13/95	1.16 (0.46-2.90)	1.13 (0.49-2.32)	lab tests (TV)	1) Two 2.5 hours small group educational meetings<br>2) One individual practice visits to discuss barriers<br>3) Homework prior to each meeting to prepare for the meetings	yes	yes	+	−	+	−	+	−	Yes
Thomas 2006	42 practices, for patients not reported	365	Not reported	0.89 (0.83-0.93)	0.90 (0.85-0.94)	lab tests (LVC)	1) The brief educational messages were added as reminders to all test result reports sent to the requesting practice	yes	no	+	+	+	+	+	+	Yes

Pinto 2018	38 practices, 239 physicians	548	I: 12.07 (13.02)<br>C: 13.08 (12.97)	0.87 (0.41-1.83)	0.92 (0.52-1.32)	other drugs (TV)	1) Three 15- 20-minute educational visits by one of 15 academic detailers. 1-3 physicians per meeting.<br>2) The detailer will also distribute a point of care summary highlighting the main messages.	no	yes	+	+	+	+	+	+	Yes
Kerfoot 2010	95 providers, 27577 patients	504	I: 1028/13089<br>C: 1906/14488	0.56 (0.30-1.07)	0.60 (0.33-1.06)	lab tests (LVC)	1) Online educational materials in series of emails. Each containing a clinical case and immediate feedback to the response. 36 emails in total.<br>2) Participants received a $75 gift certificate to an online bookstore on submission of three tests during the intervention period.	yes	yes	+	+	+	+	−	+	No
Berings 1994	85 physicians	56	Two intervention groups:<br>I1: 11.2 (5.4)<br>I2: 10.8 (6.3)<br>C: 14.2 (5.6)	0.37 (0.19-0.73)	0.77 (0.61-0.94)	other drugs (TV)	1) Three times educational material on rational prescribing by mail<br>2) One educational visit by another physician	no	no	+	−	−	−	−	+	Yes
Wächtler 2023	42 practices, 344 patients	533	I: 97/187<br>C: 68/157	2.32 (0.81-6.66)	1.48 (0.88-1.93)	Antibiotics (TV)	1) One time guideline distribution + advice to follow it	no	no	+	−	−	−	+	+	No
Oakeshott 1994	62 physicians	84	I: 8.1 (8.3)<br>C: 12.2 (15.4)	0.56 (0.22-1.38)	0.66 (0.14-1.18)	Imaging (TV)	1) Radiological guideline distribution by mail for one time	no	no	+	+	+	−	+	+	No
Goodchild 2018	2657 physicians	180	I: 105.5 (37.7)<br>C: 109.7 (40.8)	0.82 (0.72-0.95)	0.97 (0.94-0.99)	Antibiotics (TV)	1) Educational letter on antibiotic prescribing to high prescribers	no	yes	+	+	+	+	−	+	No

TB = theoretical background, BI = baseline imbalances, TV=total volume outcome, LVC=low-value care outcome

*First intervention arm had substantially larger baseline use of low-value care compared to the control group. This may influence the effect sizes as they could not be adjusted for the baseline.

Table 4. Study characteristics – Audit and feedback

Author	Number of participants	Follow-up (days)	Follow-up mean/events	OR (95% CI)	Relative reduction (ROM or RR)	Target (TV/LVC)	Intervention	Tailoring	TB	Risk of Bias						BI
Sacarny 2018	5055 physicians	730	I: 2456 (NR)<br>C: 2864 (NR)	0.66 (0.60-0.73)	0.83 (0.79-0.87)	Other drugs (LVC)	1) Three letters in six months describing that responder did prescribe more quetiapine compared to peers and encouraged to review prescribing patterns, sent to high prescribers	no	no	+	+	+	+	−	+	no
Sacarny 2016	1518 physicians	90	NR	1.07 (0.89-1.28)	1.01 (0.99-1.03)	Other drugs (LVC)	1) One letter stating higher prescribing rates of controlled substances (opioids, stimulants, methylphenidate) compared to peers, sent to high prescribers	no	no	+	+	+	−	−	+	no
Linder 2010	27 practices, 18488 patients	274	I: 3912/8406<br>C: 4761/10082	0.93 (0.56-1.54)	0.96 (0.68-1.27)	Antibiotics (LVC)	1) Individual quality dashboards about prescribing rates were implemented in EHR system, monthly reminders about its availability	no	no	+	+	+	−	+	+	no
Thomas 2006	42 practices, for patients not reported	365	NR	0.87 (0.81-0.94)	0.88 (0.83-0.95)	Lab tests (LVC)	1) Four times feedback, including a six-sided booklet presenting graphs of practice level data for each of the nine targeted tests and for each laboratory discipline, feedback included some educational content	yes	no	+	+	+	+	+	+	yes
Torrente 2020	1811 physicians	180	I: 6.60 (3.91)<br>C: 7.00 (3.99)	0.73 (0.62-0.86)	0.94 (0.89-1.00)	Other drugs (LVC)	1) Two feedback emails. The first contained evidence-based information about adequate prescribing of nimodipine and comparison to peers. The second email about information on changes in the physician's prescribing + acknowledgement or social norm message	no	no	+	+	+	+	+	+	no
Winkens 1995	79 physicians	730	Two outcomes:<br>I1: 102 (IQR 42-159)<br>C1:141 (IQR 79-190)<br>I2:101 (IQR 54-137)<br>C2: 100 (62-134)	0.68 (0.33-1.40)	0.95 (0.70-1.19)	Imaging and lab tests	1) Individual in-person feedback on diagnostic testing and imaging, including prescribing volumes and recommendations for change	no	no	+	+	+	−	−	+	yes

TB = theoretical background, BI = baseline imbalances

Table 5. Study characteristics – Provider education combined with decision support

Author	Number of participants	Follow-up (days)	Follow-up mean/events	OR (95% CI)	Relative reduction (ROM or RR)		Intervention	Tailoring	TB	Risk of bias						BI
Legare 2012	9 practices, 359 patients	180	I: 49/181<br>C: 93/178	0.34 (0.14-0.84)	0.52 (0.25-0.91)	Antibiotics (TV)	1) 2-hours online tutorial on clinical decision making in respiratory infections<br>2) 2-hours on-site interactive workshops on same concepts than tutorial, included videos, exercises decision aids<br>3) Decision aid available in walk in concultation rooms	yes	yes	+	−	−	−	+	+	yes
Cherkin 2018	6 practices, 2636 patients	180	I: 256/1163<br>C: 265/1473	1.09 (0.80-1.50)	1.07 (0.83-1.38)	Imaging (TV)	1) EHR integrated decision support tool for low-back pain<br>2) 6 x 1 hour training sessions focused on the STarT Back tool and matched treatment options for primary care teams<br>3) 30-minuts of individual coaching on how to locate and correctly use the STarT Back tool<br>4) Links in EHR to STarT Back Tool, health system's own back pain guidelines, educational resources, and self-management groups<br>5) Five days of intensive training for physical therapists	yes	yes	+	+	+	+	+	+	yes
Phuong 2010	6 practices, 2019 patients	1460	I: 907/1120<br>C: 809/899	0.47 (0.10-2.29)	0.90 (0.52-1.06)	Antibiotics (TV)	1) 2-days training on clinical signs and symptoms of infectious diseases<br>2) Diagnostic algorithm	no	no	+	−	−	−	+	+	yes
Fender 1999	100 practices, 563 patients	365	I: 82/375<br>C: 44/186	0.90 (0.51-1.58)*	0.92 (0.58-1.39)	Other drugs (LVC)	1) Two educational visits, including presentation of current evidence base<br>2) Educational material distributed in the meetings<br>3) Flow chart introducing the appropriate treatment pathways	no	yes	+	−	−	−	+	+	no

TB = theoretical background, BI = baseline imbalance, TV=total volume outcome, LVC=low-value care outcome

*Authors reported OR of 0.69 (95% CI 0.43-1.10) adjusted for practice characteristics, but it was unclear if the estimate was adjusted for clustering, so we used event rates instead to calculate odds ratio

Table 6. Study characteristics – Provider education combined with audit and feedback

Author	Number of participants	Follow-up (days)	Follow-up mean/events	OR (95% CI)	Relative reduction (ROM or RR)	Target	Intervention	Tailoring	TB	Risk of bias						BI
O'Connell 1999	2440 providers	300	I: 79.2 (26.7)<br>C: 77.6 (25.2)	1.03 (0.89-1.19)	1.02 (0.99-1.05)	Other drugs (TV)	1) Two times feedback, graphical display on prescription rates for antibiotics, NSAIDs, lipid-lowering drugs, ACE-inhibitors and H_2-reseptor antagonists<br>2) Educational newsletter alongside prescription rate feedback	no	no	+	+	+	−	+	+	no
Hemkens 2017	2814 providers	730	I: 91. 0 (38.1)<br>C: 92.7 (42.1)	0.93 (0.81-1.06)	0.98 (0.95-1.01)	Antibiotics (TV)	1) Guideline developement with feedback from local experts<br>2) Quarterly mailed feedback on precribing rates for two years<br>3) Educational material (guidelines)	yes	no	+	+	+	+	−	+	no
Hallsworth 2016	803 practices	180	I: 132.9 (20.8)<br>C: 136.2 (15.9)	0.66 (0.51-0.85)	0.97 (0.95-0.99)	Antibiotics (TV)	1) One letter stating higher prescribing rates compared to peers<br>2) Letter included additionally educational content on alternative treatment paths	yes	yes	+	+	+	+	+	+	no
Fenton 2016	61 providers	365	Not reported	1.07 (0.49-2.32)	1.05 (0.56-1.73)	Imaging (LVC)	1) simulated patients who gave feedback after the visit + education on core elements of patient-centered care	yes	yes	+	+	+	+	−	+	no
Hurlimann 2015	133 providers	365	I: 25/522<br>C: 21/450	1.02 (0.36-2.88)	1.02 (0.37-2.65)	Antibiotics (LVC)	1) guideline development and distribution<br>2) Once a week mailed(?) reminders, including individual feedback on antibiotic prescription pattern, compared with the other members of the intervention group.	no	no	+	+	+	−	−	+	no
Rognstad 2013	80 practices, 449 providers	365	I: 22.4 (10.5)<br>C: 24.2 (10.1)	0.73 (0.46-1.17)	0.93 (0.82-1.03)	Other drugs (TV)	1) Two educational visits on safer prescribing for older patients<br>2) One-day workshop<br>3) Three times feedback, prescribing rates compared to peers, including suggestions for alternative and safer treatment options	no	yes	+	+	+	+	+	+	yes

Gjelstad 2013	79 practices, 133258 patients	365	I: 21246/66757 C: 23307/66501	0.72 (0.61-0.84)	0.80 (0.71-0.89)	Antibiotics (TV)	1) Two educational visit on guidelines, and encouragement on delayed prescribing<br>2) Two times feedback, individual reports, were also discussed in group meeting<br>3) Pop-ups in EHR that prompted to record if prescription was delayed and number of days delayed<br>4) One-day educational seminar	yes	yes	+	+	+	−	+	+	no
Dormuth 2012	2725 providers, 1706787 patients	365	I: 9252/820525 C: 9797/886262	1.02 (0.73-1.43)	1.02 (0.73-1.42)	Other drugs (TV)	1) One time educational material on statin prescribing including 3 patient cases and educational messages<br>2) Feedback provided in the same letter mail with prescribing rate comparison to total prescribing in the province	no	no	+	+	+	−	−	+	no
Butler 2012	68 practices	365	I: 664 (NR) C: 681 (NR)	0.35 (0.15-0.86)	0.96 (0.92-0.99)	Antibiotics (TV)	1) Online materials including patient cases, evidence summaries, guidelines and videos, web forum to interact with others and which provided updates on new evidence, continuous access<br>2) One educational meeting, including prescribing feedback and prescribing rate comparison to peers	yes	yes	+	+	+	+	+	+	no
Cals 2009	10 practices	180	I: 56/201 C: 123/230	0.59 (0.41-0.83)	0.75 (0.60-0.91)	Antibiotics (TV)	1) Two-hour educational seminar on communication skills<br>1) Two simulated patient visits the GPs and record the audio which were self-evaluated and peer evaluated	no	yes	+	−	+	+	+	+	no
Verstappen 2003 (a)*	26 practices, 163 providers	180	I: 45 (41) C: 63 (56)	0.41 (0.22-0.67)	0.75 (0.57-0.89)	Lab tests (LVC)	1) Three times mailed personalised graphical feedback on prescribing including comparison to peers<br>2) guideline dissemination<br>3) Three small group quality improvement meetings including local consensus plans for improvement	no	no	+	+	+	+	+	+	no
Verstappen 2003 (b)	26 practices, 163 providers	180	I: 138 (74) C: 126 (74)	0.64 (0.36-1.12)	0.94 (0.87-1.01)	Lab tests (LVC)	1) Three times mailed personalised graphical feedback on prescribing including comparison to peers<br>2) guideline dissemination<br>3) Three small group quality improvement meetings including local consensus plans for improvement	no	no	+	+	+	+	+	+	no

Sharma 2002	20 practices, 1488 patients	60	two TV outcomes: I: 252/390 C:218/252 I: 322/392 C: 278/346	0.92 (0.58-1.61)	0.97 (0.79-1.16)	Antibiotics (TV)	1) Two one-day workshops including interactive discussions.including forming consensus on the treatment. 2) Audit results and feedback provided in workshops 3) Educational material. Written information material, such as WHO guidelines, and copies of the national programmes and the relevant publications.	yes	no	+	+	−	−	+	+	yes
Trietsch 2017	21 practices, 88 providers	1060	Several TV outcomes	0.83 (0.45-1.53)	0.94 (0.70-1.08)	Multiple drugs and labs (TV)	1) Two 90-minutes small group discussions including feedback on test ordering and prescribing guidelines 2) Local consensus were made about preferred prescriptions during the meetings 3) At the start of each meeting, each GP received a feedback report on their performance & recommendations from guidelines; comparison to peers.	no	yes	+	+	+	+	+	+	no
Aghlmandi 2023	3170 providers	730	Median (IQR) I: 8.2 (6.1-11.4) C: 8.4 (6.0-11.8)	0.98 (0.87-1.12)	1.00 (0.99-1.01)	Antibiotics (TV)	1) Quarterly mailed prescribing feedback including peer-comparison for two years 2) A call to action information in a blue box, which varied with each mailing, 3) Evidence-based guidelines on antibiotic prescribing for respiratory tract and urinary tract infections	no	no	+	+	+	+	−	+	no
Schmiemann 2023	110 practices, 203 physicians	365	mean proportion: I: 0.19 (0.20) C: 0.35 (0.25)	0.31 (0.15-0.58)	0.60 (0.31-0.89)	Antibiotics	1) One Educational visit including information on guideline recommendations and on regional resistance data and communication strategies 2) Quarterly feedback of prescribing behaviour for FPs. including comparison to peer practices via telephone call quarterly + also in print or electronic form	yes	no	+	+	−	−	+	+	yes
Dutcher 2022	31 practices, 185 755 visits	90	Stepped-wedge trial: Baseline: 17410/90677 follow-up: 5011/44345	0.57 (0.53-0.61)	0.62 (0.58-0.66)	Antibiotics	1) One time educational session on appropriate prescribing and communication strategies - participant could also choose watching prerecorded video instead 2) Monthly feedback report by email, including comparison to peers	no	no	+	−	+	+	+	+	yes

Goodchild 2018**	5301 physicians	180	C: 109.7 (40.8)<br>I1: 98.7 (50.1)<br>I2: 97.9 (38.9)<br>I3: 96.2 (37.4)	0.59 (0.53-0.66)	I1: 0.91 (0.86-0.94)<br>I2: 0.90 (0.87-0.92)<br>I3: 0.88 (0.85-0.90)	Antibiotics	Including 3 different intervention arms:<br>1) Educational letter on antibiotic prescribing to high prescribers<br>+ 2) Feedback on individual prescribing rates and comparison to peers<br>OR 3) Previous components + extra graphical display for the prescribing<br>OR 4) Third + educational material on delayed prescribing	no	yes	+	+	+	+	−	+	no
Baker 2003	33 practices, 96 physicians	365	multiple outcomes	0.84 (0.28-2.54)	***	Lab tests	1) Lead physician in each practice received feedback report and were asked to distribute copies of it and discuss with others<br>2) The first feedback post included guidelines on test ordering	no	no	+	+	−	+	+	+	yes
Carney 2023	4833 physicians	365	multiple outcomes	0.67 (0.53-0.85)	0.70 (0.56-0.86)	Antibiotics	1) Educational portrait including data on antibiotic resistance,recommendation to use nitrofurantoin, and a patient case, the portrait was sent twice by mail<br>2) The portrait included also feedback on the individual prescribing rates	no	no	+	+	+	−	+	+	no
Bonney 2023	101 physicians	180	multiple outcomes	0.96 (0.52-1.79)	0.97 (0.83-1.10)	Lab tests, imaging, several drugs	1) Educational webinar for one hour<br>2) Participants audited study related patients - data used only by the GP<br>3) Case preparation for a webinar<br>4) 1-hour webinar with the patient cases and discussion about the management<br>webinars were recorded and were available online	no	yes	+	+	+	+	−	+	no

TB = theoretical background, BI = baseline imbalances, TV=total volume outcome, LVC=low-value care outcome
*Study had two intervention arms with same intervention but different laboratory test targets
**Study had multiple intervention groups, which were combined before the meta-analysis
*** ROM not possible to estimate Due to large variance (and small sample)

Table 7. Study characteristics – Patient education

Author	Number of participants	Follow-up (days)	Follow-up mean/events	OR (95% CI)	Relative reduction (ROM or RR)	Target	Intervention	Tailoring	TB	Risk of bias						BI
Macfarlane 2002	205 patients	14	I: 49/104<br>C: 63/101	0.54 (0.31-0.93)	0.76 (0.54-0.98)	Antibiotics (LVC)	1) One educational leaflet for patients about antibiotic use and delayed prescribing	no	no	+	+	−	+	+	+	no
Meeker 2014	14 physicians, 235 patients	90	I: 41/114<br>C: 59/121	0.59 (0.20-1.78)	0.74 (0.32-1.29)	Antibiotics (LVC)	1) Displaying poster-sized commitment letters in examination rooms	no	yes	+	−	+	+	+	+	no
Lee 2017*	914 patients	0	I: 94/457<br>C: 81/457	1.20 (0.83-1.73)	1.16 (0.86-1.53)	Antibiotics (TV)	1) Three minutes patient educational counceling on antibiotic use in respiratory infections<br>2) Educational pamphlets to patients given in the counceling session	yes	no	+	+	+	+	+	+	no
Agnew 2013	4 practices, 115 patients	35	I: 20/46<br>C: 50/69	0.29 (0.06-1.53)	0.60 (0.40-0.82)	Antibiotics (TV)	1) One patient information leaflet alongsde delayed prescription	no	no	+	−	+	−	+	+	yes

TB = theoretical background, BI = baseline imbalances, TV=total volume outcome, LVC=low-value care outcome
*control group received education on influenza (initially thought to have no impact on antibiotic use)

Table 8. Study characteristics – Patient education combined with provider education

Author	Number of participants	Follow-up (days)	Follow-up mean/events	OR (95% CI)	Relative reduction (ROM or RR)	Target	Intervention	Tailoring	TB	Risk of bias						BI
de Burgh 1995	186 providers, 530 patients	120	Two outcomes<br>I: 145/311<br>C: 154/286<br>I: 242/277<br>C: 224/253	0.81 (0.60-1.03)	0.94 (0.84-1.01)	Other drugs (TV)	1) 20-minutes (individual?) educational visit + follow-up call on benzodiazepine prescribing<br>2) One-time educational material (guidelines)<br>3) Educational materials for patients about improving sleep	no	no	+	+	−	+	−	+	no
Little 2013	227 practices	365	I: 476/1170<br>C: 508/870	0.48 (0.33-0.74)	0.69 (0.54-0.87)	Antibiotics (TV)	1) Two internet trainings on enhanced communication skills<br>2) Interactive booklet to use during consultations, information on symptoms, use of antibiotics and antibiotic resistance, self-help measures, and when to re-consult<br>3) Meeting on prescribing issues	yes	yes	+	−	−	−	+	+	no
Altiner 2007	87 providers, 1707 patients	365	I: 289/787<br>C: 596/920	0.55 (0.38-0.80)	0.78 (0.64-0.92)	Antibiotics (TV)	1) One educational individual visit by GP peer, the educators used a semi- standardized dialogue script to distribute their message using communicative techniques derived from the elaboration likelihood model.<br>2) Patient leaflets and a poster in the waiting room	yes	yes	+	−	−	−	−	+	yes
Avorn 1983	281 providers	245	I: 4174 (NR)<br>C: 4921 (NR)	0.43 (0.28-0.65)	NA	Antibiotics + other drugs (TV)	1) One personal educational visit on communication and prescribing<br>2) Educational materials sent several times<br>3) Patient brochures, given by physicians	yes	yes	+	+	+	+	−	+	no

Hemminki 2008	20 practices, 1563 patients	577	I: 161/845<br>C: 116/723	1.23 (0.56-2.7)	1.19 (0.60-2.12)	Surgery (TV)	1) One 1,5-hour educational meeting: instructional conversation in small groups<br>2) A leaflet on childbirth and preparation for it, which PHNs were asked to give to the pregnant women and to discuss its contents both during childbirth classes and other visits<br>3) A file of evidence-based research material given once to the study centers<br>4) One time questionnaire on opinions and knowledge of childbirth	no	no	+	+	+	+	+	+	no
Dey 2004	23 practices, 2187 patients	240	I: 158/1049<br>C: 157/1138	1.11 (0.75-1.64)	1.09 (0.78-1.51)	Imaging (TV)	1) Two individual educational visits<br>2) GPs were given a poster reinforcing guideline recommendations and a copy of a text recommended by the RCGP for patients<br>3) Referral forms for access to fast-track physiotherapy were given to GPs<br>4) Practices were revisited when their experiences of implementing the guidelines were discussed.	no	yes	+	−	+	−	+	+	no
Simula 2021	8 practices, 364 patients	365	I: 19/178<br>C: 31/186	0.52 (0.32-0.84)	0.60 (0.40-0.88)	Imaging (TV)	1) Educational booklet for the patient to be used in the consultation<br>2) 30-minutes educational meeting for professionals on how to use the booklet	no	yes	+	−	+	−	+	+	yes
Kullgren 2024	8 practices, 81 physicians, 83497 patients	108	Stepped-wedge trial,<br>C: 7627/37116<br>I:7416/46381	0.79 (0.65-0.97)	0.82 (0.70-0.98)	Lab tests and other drugs (LVC)	1) Written precommitment to 3 choosing wisely recommendations<br>2) Photographs of committed clinicians appeared on posters in<br>public waiting areas and examination rooms<br>3) Choosing Wisely educational handouts were mailed to applicable patients before scheduled visits and available at the point of care.<br>4) Clinicians received a weekly email with strategies to avoid use of low-value services during patient encounters	yes	yes	+	+	+	+	+	+	yes

Llor 2024*	10 practices, 96 patients	42	I: 19/56<br>C: 17/30	0.38 (0.15-0.94)	0.58 (0.29-0.97)	Antibiotics (TV)	1) 2-hours workshop on communication skills<br>2) Monthly internet-based educational training capsules including clinical cases and reminders<br>3) Educational booklets for patients, used during the consultation and given to patients. Including information on symptoms, use of antibiotics and antibiotic resistance, self-help measures, and when to re-consult.			+	−	+	−	+	+	yes
Seager 2006	70 physicians, 1497 patients	not reported (likely short)	Two intervention groups:<br>I1: 39/556<br>I2: 68/451<br>C: 88/490	OR1: 0.33 (0.21-0.54)<br>OR2: 0.82 (0.53-1.29)	RR1 0.38 (0.24-0.59)<br>RR2 0.85 (0.58-1.23)	Antibiotics (LVC)	2 intervention groups, first got component 1 and second got both:<br>1) Educational material including guidelines, summary of recommendations, and patient education leaflets<br>2) One academic detailing visit	yes	no	+	−	−	−	+	+	yes

TB = theoretical background, BI = baseline imbalances

Table 9. Study characteristics – Patient education combined with provider education and decision support

Author	Number of participants	Follow-up (days)	Follow-up mean/events	OR (95% CI)	Relative reduction (ROM or RR)	Target	Intervention	Tailoring	TB	Risk of bias						BI
Gulliford 2014	104 practices	365	I: 52 (range 45-58)<br>C: 52 (range 45-59)	0.47 (0.23-0.96)	0.96 (0.93-1.00)	Antibiotics (TV)	1) Decision support tool in EHR, which activated with medical code for respiratory track infection<br>2) Educational material (within the EHR tool)<br>3) Training video on EHR tool<br>4) Decision support included educational material for patients	no	yes	+	+	+	−	+	+	no
McIsaac 1998	377 patients	0	I: 48/173<br>C: 72/204	0.70 (0.56-1.06)	0.79 (0.56-1.06)	Antibiotics (TV)	1) Clinical decision support including scoring system and recommendations on paper<br>2) Educational material about the decision support<br>3) Self-help educational material for patients	no	no	+	+	−	−	−	+	no
Hartman 2023	39 practices, 1041 patients	150	I: 23/502<br>C: 41/539	0.53 (0.24-1.14)	0.44 (0.18-1.04)*	Antibiotics (LVC)	1) A decision tool to guide appropriate antibiotic use<br>2) A toolbox of educational materials, such as pocket cards, posters, and information leaflets. These materials targeted healthcare professionals as well as patients and informal caregivers<br>3) One or more educational sessions were held with general practitioners and nursing staff, with a median duration of 60 minutes<br>4) One evaluation session took place with a median duration of 30 minutes	yes	yes	+	−	+	+	+	+	yes

*Adjusted for baseline by study authors

Table 10. Study characteristics – Patient education combined with audit and feedback, and provider education

Author	Number of participants	follow-up (days)	Follow-up mean/events	OR (95% CI)	Relative reduction (ROM or RR)	Target	Intervention	Tailoring	TB	Risk of bias						BI
Shen 2018	24 practices, 516 patients	38	I: 157/262<br>C: 192/254	0.48 (0.25-0.94)	0.79 (0.57-0.98)	Antibiotics (TV)	1) Commitment letters presented in the clinic and backside of patient information leaflet, available continuously<br>2) Audit and feedback, expert panel checked patient records and gave scores + prescribing percentages<br>3) Web-based system providing the feedback also had educational material on diagnostics, shared decision making and patient education	no	no	+	−	−	+	+	+	no
Zwar 1999	157 physicians	180	I: 19.7 (14.4)<br>C: 31.7 (19.2)	0.28 (0.16-0.50)	0.62 (0.45-0.79)	Antibiotics (TV)	1) Participants audited 110 consecutive patients, investigators gave mailed feedback twice<br>2) Twice educational material (guidelines)<br>3) Patient educational material, handouts given by the clinicians<br>4) 20-minutes educational visit or phone call to others but best performers	no	yes	+	+	−	−	−	+	no
Hallsworth 2016	790 practices	180	I: 132.9 (20.8)<br>C: 136.2 (15.9)	0.72 (0.56-0.93)	0.98 (0.96-0.99)	Antibiotics (TV)	1) One letter stating higher prescribing rates compared to peers<br>2) Letter included additionally educational content on alternative treatment paths<br>3) posters and leaflets for patients<br>4) An educational letter addressed to the practice manager - stating higher prescribing peers	yes	yes	+	+	+	+	+	+	no
Welschen 2004	12 practices, 89 physicians	365	I: 23 (15.6)<br>C: 37 (18.1)	0.43 (0.20-0.92)	0.62 (0.42-0.82)	Antibiotics (TV)	1) One educational meeting including consensus process and training on communication skills<br>2) Two times feedback, including peer comparison<br>3) Educational material for patients - brochures and posters available continuously in waiting rooms<br>4) Consensus process leading to local guidelines	yes	no	−	+	+	−	+	+	yes

Schwartz 2024	5046 physicians	365	I: 10.3 (40.9)<br>C: 11.4 (12.0)	0.84 (0.74-0.96)	0.92 (0.91-0.94)	Antibiotics (LVC)	1) Feedback letters sent twice to GP clinics - including a graph, education on appropriate antibiotic prescribing, communication strategies, and tools from Choosing Wisely Canada<br>2) The letters also included a prescription pad for patients describing the natural course of symptoms and harms from antibiotics<br>3) Half of the intervention group were randomized to feedback including adjusted prescribing comparison graph and half to harms of antibiotics messaging (2x2 trial)	yes	yes	+	+	+	+	−	+	no

TB = theoretical background, BI = baseline imbalance

Table 11. Study characteristics – studies with educational material as control

Provider education																	
Study	Number of participants	Follow-up (days)	Follow-up mean/events	OR (95% CI)	Relative reduction (ROM or RR)	Target	Intervention	Tailoring	TB	Risk of bias						BI
French 2013	78 practices, 152942 patients	365	I: 1117/77716 C: 1264/75226	0.85 (0.69-1.05)	0.86 (0.69-1.05)	Imaging (TV)	1) Two 3-hour workshops including small group discussions and didactic lectures.<br>For non-attendants: DVD about the workshops and educational material	yes	yes	+	−	+	−	+	+	No
Eccles 2001	122 practices	365	Two outcomes:<br>I: 5.14 (3.7)<br>C: 6.80 (4.3)<br>I: 5.22 (3.6)<br>C: 7.02 (3.6)	0.44 (0.23-0.84)	0.75 (0.55-0.95)	Imaging (TV)	1) Local guidelines by radiologists and distribution once(?)<br>2) Continuous educational messages in radiagraphy reports	yes	no	+	+	−	+	+	+	No
Coenen 2004	71 physicians, 693 patients	120	I: 80/292<br>C: 115/401	0.56 (0.36-0.87)	0.64 (0.44-0.90)	Antibiotics (TV)	1) One time guideline distribution<br>2) One educational outreach visit<br>3) One phone call from one of two facilitators<br>4) One mail reminder	yes	yes	+	−	−	−	−	+	Yes
French 2022	210 practices, 1358 patients	120	I: 23/755<br>C: 16/603	1.4 (0.51-3.87)	1.48 (0.88-1.93)	Imaging (TV)	1) One full day symposium delivered by peer opinio leaders, small group discussions<br>2) DVD of lectures + written material<br>3) One follow up phone call	yes	yes	+	+	+	−	+	+	No
Driel 2007	18 practices, 61 physicians, 208 patients	60	not reported	0.73 (0.39-1.35)	-	Antibiotics (TV)	1) Guideline dissemination by mail<br>2) Moderators of quality circles (local GPs) received edcuational visit from trained academic detailer.<br>3) Moderators held local small group educational meetings including patient education materials, flowcharts, recommendations, scientific evidence.	no	no	+	−	−	−	+	+	Yes

Audit and feedback

Study	Number of participants	Follow-up (days)	Follow-up mean/events	OR (95% CI)	Relative reduction (ROM or RR)	Target	Intervention	Tailoring	TB	Risk of bias						BI
Eccles 2001	122 practices	365	Two outcomes:<br>I: 5.97 (4.2)<br>C: 6.80 (4.3)<br>I: 6.32 (4.0)<br>C: 7.02 (3.6)	0.71 (0.37-1.36)	0.89 (0.70-1.09)	Imaging (TV)	1) Local guidelines by radiologists and distribution once(?)<br>2) practice level radiograph rates compared to other practices - sent twice to the participants	yes	no	+	+	−	+	+	+	No

Audit and feedback combined with Provider education

Study	Number of participants	Follow-up (days)	Follow-up mean/events	OR (95% CI)	Relative reduction (ROM or RR)	Target	Intervention	Tailoring	TB	Risk of bias						BI
Eccles 2001	122 practices	365	Two outcomes:<br>I: 5.23 (3.7)<br>C: 6.80 (4.3)<br>I: 5.21 (3.7)<br>C: 7.02 (3.6)	0.45 (0.24-0.86)	0.75 (0.55-0.96)	Imaging (TV)	1) Local guidelines by radiologists and distribution once(?)<br>2) Continuous educational messages in radiography reports<br>3) practice level radiograph rates compared to other practices - sent twice to the participants	yes	no	+	+	−	+	+	+	Yes
Meeker 2016	10 practices, 3715 patients	540	I: 311/1620<br>C: 502/2095	0.75 (0.28-2.04)	0.80 (0.34-1.63)	Antibiotics (LVC)	1) Brief online education<br>2) Audit and feedback via email, including peer comparison	no	yes	+	−	+	+	+	+	Yes
Sondergaard 2003	181 practices	90	I: 34.6 (15.9)<br>C: 34.0 (12.3)	1.08 (0.64-1.84)	1.01 (0.90-1.14)	Antibiotics (TV)	1) educational material (guidelines)<br>2) audit and feedback, mailed prescription rates with peer comparison<br>3) guideline development	yes	no	+	+	+	−	+	+	No

Provider education combined with Decision support																
Meeker 2016	12 practices, 4483 patients	540	I: 722/2388<br>C: 502/2095	1.38 (0.60-3.17)	1.26 (0.66-2.09)	Antibiotics (LVC)	1) Brief online education<br>2) Pop-up decision support in EHR when diagnosis of respiratory tract infection, suggested alternative treatments	no	yes	+	–	+	+	+	+	Yes
McGinn 2013	168 physicians, 984 patients	365	I: 171/586<br>C: 153/398	0.66 (0.46-0.95)	0.76 (0.58-0.97)	Antibiotics (TV)	1) Training on clinical prediction rules<br>2) Clinical calculator with management recommendations triggered in EHR according to prespecified criteria	yes	no	+	–	+	–	+	+	No
Persell 2024	60 practices, 371 physicians	548	Three outcomes:<br>I:<br>28.5 (8.7)<br>16.7 (8.3)<br>24.2 (9.0)<br>C:<br>32.4 (8.9)<br>15.9 (7.9)<br>24.9 (8.6)	0.69 (0.52-0.92)	0.76 (0.60-0.91)	Lab tests and other drugs (TV)	1) Decision support - pop up in EHR system giving educational guidance on specific situations<br>2) Email link to educational material + introduction to the decision support, one time	yes	yes	+	+	+	–	+	+	Yes

Table 12. Study characteristics – Trials ineligible for the meta-analysis

Study	Number of participants	Follow-up (days)	Follow-up mean/events	Reported effect size	Target	Intervention category	Intervention	Control	Tailor	TB	Risk of bias						BI	Reason for exclusion
Kullgren 2018	6 practices, 45 providers, 18017	135	NR	Difference in proportion: -0.014 (-0.029-0.001)	Low value service use (TV)	Provider education + Patient education	1) Invitation to precommit for CW recommendations<br>2) Precommitted physicians received 1–6months of point-of-care reminders<br>3) A patient education handout<br>4) Weekly emails with links to improve patient communication	Stepped wedge trial	no	yes	+	−	+	+	+	+	yes	Reported only difference in proportion
McNulty 2018	150 practices	365	NR	Rate ratio: 0.97 (0.95-1.00)	Antibiotics (TV)	Provider education + Audit and feedback + Patient education	1) 1-hour workshop on guidelines and clinical cases<br>2) Audit and feedback, feedback during workshops<br>3) Posters and videos for patients, and leaflets to share during consultations<br>4) Educational materials (checklist and guidelines)	No intervention	no	yes	+	+	+	−	+	+	no	Only rate ratio reported
Ray 2001	220 physicians	365	NR	DiD: -21.3 (-32.4 to -10.2), about 7% decrease relative to control	NSAID (TV)	Provider education + Decision aid + Patient education	1) key messages card, flowchart for re-evaluation of NSAID users, and journal articles<br>2) One individual educational visit<br>3) Reminders in the patient charts including decision aid and educational materials for patients	No intervention	yes	yes	+	+	+	−	−	+	no	Reported only difference in difference

Schectman 2003	14 practices, 2020 patients	365	NR	NR	(LVC)	Provider education + Audit and feedback + Patient education	3 intervention groups: first group[3,4,5,7], second[2,6], third[all]<br>1) Guideline developement<br>2) Educational materials including pamphlet and videotape for patients, sent to clinical sites<br>3) Educational session for clinicians<br>4) Audit and feedback, report given after the educational session<br>5) Individual follow up visit<br>6) Reminders to use patient materials<br>7) For non-attenders - guidelines, feedback report and phone call	No intervention	no	no	+	+	+	−	+	+	yes	Not enough data
Kerry 2000	69 practices	365	NR	relative difference: -10% (-21%-1%)	imaging (TV)	Provider education + Audit and feedback	1) Guideline development, possibility to give feedback on them before final version<br>2) One time feedback on individual prescribing rates before and after the first guideline distribution	No intervention	yes	no	+	+	+	−	+	+	yes	Not enough data
Ilett 2000	112 physicians	90	NR	NR	Antibiotics (TV)	Provider education	1) Guideline developement including short recommendations on primary treatment options<br>2) One 10-15 minutes individual educational visits<br>3) Visits included distribution of laminated version of the guideline	No intervention	no	no	+	+	+	+	−	−	yes	not enough data
Bernal-Delgado 2002	24 practices, 158 physicians	180	NR	I: -22.5% (34.42 to −10.76), Placebo: - 9.78% (−17.70 to −1.86) C: +14.44% (5.22 to 23.66)	NSAID (TV)	Provider education	1) Educational material distributed during meetings<br>2) One educational session about effectiveness and safety of NSAIDs	No intervention	no	yes	+	+	+	−	+	−	no	reports only relative reduction

van Eijk 2001	21 Groups, 227 providers	240	NR	Rate ratio: 0.69 (0.50-0.95)	Anticholinergic antidepressants (TV)	Provider education + Audit and feedback	1) Two individual educational visits<br>2) Educational leaflet<br>3) Audit and feedback, Feedback given during the second individual meeting	No intervention	no	yes	+	+	+	−	+	+	yes	reports only rate ratio
Vervloet 2016	8 clusters	365	NR	p-value 0.09	Antibiotics (TV)	Provider education + Audit and feedback + Decision aid + Patient education	1) EHR alert suggesting no prescription when diagnosing respiratory tract infection<br>2) Alert included suggestion on advice to give to patients instead<br>3) Two educational meetings on communication skills and antibiotic treatment indications<br>4) Audit and feedback, including quarterly feedback and discussion in a meeting	education on another topic	no	no	+	−	+	−	+	−	yes	not enough data
Yip 2014	28 clusters	540	NR	absolute -6.6% decrease from 44.2% (control), p < 0.05 in towns. -6.0% from 34.2%, p < 0.05	Antibiotics (TV)	Financial + Provider education	1) Governmental payments to township health centers and village posts from fee- for-service to a capitated budget with pay-for-performance. Each center that scored above the average on performance score, received more than the 30 percent of the budget that had been withheld.<br>2) Training on drug prescriptions	Same training on drug prescriptions as in the intervention group	no	no	+	+	+	−	+	+	no	not enough data
Milos 2013	15 clusters, 109 physicians	180	Follow-up mean antibiotics prescription rate per 1000 inhabitants:<br>I1: 80<br>I2: 78<br>C: 82	NR	Antibiotics (TV)	Provider education	Two intervention groups:<br>1) Graded task intervention, addressing the GP's belief in his/her capabilities to manage URTIs without prescribing an antibiotic<br>2) Persuasive communication intervention, aim of influencing the GP's belief about the positive consequences of managing URTIs without prescribing an antibiotic	Usual care	no	yes	+	+	−	−	+	−	yes	not enough data

Naughton 2009	98 clusters	365	NR	NR, "no significant decreases"	Antibiotics (TV)	Provider education + Audit and feedback	1) Feedback - individual prescribing rates compared with peers<br>2) One 15-30 minutes individual educational visit about prescribing rates and on reducing antibiotic use	Similar paper feedback	no	no	+	+	+	+	+	−	yes	not enough data
Simon 2006	15 clusters, 239 providers	548	NR	p-value 0.52	Potentially inappropriate prescriptions (TV)	Provider education + Decision aid	1) Alerts, occurred at the time of prescribing a targeted potentially inappropriate medication, suggested an alternative medication<br>2) One educational meeting, including interactive components<br>3) Educational material given in the meetings and once as a reminder later	only component 1 (alerts)	yes	yes	+	+	+	−	+	−	no	not enough data
Awad 2006	10 clusters	90	I: 3.9 (0.9)<br>C: 6.9 (1.6)	NR	Antibiotics (LVC)	Audit and feedback	1) Audit and feedback including prescribing rates and individual feedback on how to improve	No intervention	yes	no	+	−	+	−	+	+	no	not enough data
Awad 2006	10 clusters	90	Two intervention groups:<br>I1: 1.6 (0.5)<br>I2: 0.9 (0.3)<br>C: 6.9 (1.6)	NR	Antibiotics (LVC)	Provider education + Audit and feedback	1) Audit and feedback including prescribing rates and individual feedback on how to improve<br>2) Two long individual educational visits including treatment discussion	No intervention	yes	no	+	−	+	−	+	+	no	+
Eltayeb 2005	10 clusters	60	Two intervention groups:<br>I1: 16.0 (2.6)<br>I2: 14.6 (2.5)<br>C: 26.8 (3.9)	mean difference:<br>-11.6 (-16.7 to - 6.6)<br>-14.2 (-19.2 to - 9.2)	other drugs (LVC)	Provider education + Audit and feedback	1) Audit and feedback - prescribing patterns within the region and within each health center, including data explanation<br>2) Routine educational meetings (academic detailing)/two educational seminars + prescribing guideline	No intervention	no	no	+	−	−	−	+	+	yes	not enough data
Pimlott 2003	374 physicians	365	NR	p-value 0.036	benzodiazepines (TV)	Provider education + Audit and feedback +	1) Audit and feedback, graphical presentation, comparison to peers<br>2) Feedback included educational	education and feedback	no	no	+	+	+	−	+	−	no	not enough data

						Patient education	material<br>3) Patient educational handouts	on another topic										
Anderson 1996	54 physicians	180	NR	First group: -25%<br>Second group: -34%<br>Control: +3%	analgesic prescriptions (TV)	Provider education + Audit and feedback	2 intervention groups: with components 1 and both 1 and 2:<br>1) One written letter notification of excessive prescribing<br>2) One-day educational seminar including small group discussions	No intervention	no	yes	+	+	+	+	−	+	yes	not enough data
Hadiyono 1996	33 clusters	90	NR	Difference proportion: -0.18 (-0.31 to -0.06	injections (TV)	Provider education + Patient education	1) One interaction group discussion with patients and prescribers involved, for about 2 hours	No intervention	no	yes	+	+	+	−	+	+	no	not enough data
Ruang-kanchanasetr 1993	36 providers	365	NR	only reported for subgroups	laboratory tests (TV)	Provider education + Audit and feedback	1) Chart audit & feedback on laboratory use<br>2) Education on sensitivity, specificity, predictive value & cost of testing.<br>3) Second year students participated in reviewing first year residents' data.<br>4) Simulated cases evaluated & rated by clinical staff.	Usual care	no	no	+	+	−	+	−	+	yes	not enough data
van der Velden 2016	86 clusters	730	NR	p-value 0.015, first year prescribing decrease of 7.6% intervention vs 0.4% control, second year -4.3% vs 2% increase	Antibiotics (TV)	Provider education + Audit and feedback + Patient education	1) Self registration of all RTI consultations.<br>2) One 1-1.5 hour educational meeting on guidelines, patient cases, antibiotic related problems, communication tools and feedback on individual prescribing patterns<br>3) Local GPs made practice improvement plans.<br>4) GPs gave patient booklets	Similar interventions on proton pump inhibitors	no	no	+	+	+	+	+	+	no	not enough data

Goldberg 2001	10 clusters	931	NR	"estimated decline of 20.9 operations per 100,000, a relative reduction of 8.9% (P = 0.01)."	surgery (TV)	Provider education + audit and feedback	1) Educational activities tailored to each study site including small group meetings and 2 conferences<br>2) Audit and feedback, peer comparison summaries quarterly<br>3) Educational material to implement shared decision making<br>4) Opinion leaders helped with tailoring and distribution	No intervention	yes	no	+	+	+	+	+	+	yes	not enough data
Sallis 2020	130 clusters, 1287 providers	180	NR	Difference in difference: -12.58 per 1000 (-30.73 to 5.58), baseline 463 per 1000	Antibiotics (TV)	Patient education + other	1) Phone educational message for patients when calling for appointment<br>2) Commitment posters in consultation rooms including relevant GP's photograph, signature, commitment letter and pledge statement	Usual care	yes	no	+	+	+	+	+	+	no	Reported only difference in proportion
Vicentini 2019	15 providers, 117 patients	180	NR	DiD:<br>- 0.08 (-0.17 to 0.01), baseline 0.117 average daily dose per participant	NSAID (TV)	Financial + Provider education	1) Free paracetamol medications<br>2) Brief information of the opportunity to prescribe free paracetamol for prescribers	Short course on pain control in osteoarthritis for all	no	no	+	−	+	+	+	+	yes	Reported only difference in proportion
Bexell 1996	16 clusters, 2031 patients	120	Proportion of patients receiving antibiotics:<br>I: 34.2%<br>C: 42.1%	p-value 0.004	Antibiotics (TV)	Provider education	1) Educational seminars: standard treatment guidelines for common conditions seen in primary care. Discussions on barriers for rational drug prescribing, Diagnosis and treatment.	Usual care	no	no	+	+	+	−	+	+	yes	not enough data
Midlöv 2005	15 clusters, 54 providers	240	NR	relative difference: -25.8% (-44.2% to -1.32%)	benzodiazepines (TV)	Provider education	1) One educational meeting on different causes of confusion in the elderly and the effects of BDZ and psychotropic drugs in the elderly	No intervention	no	no	+	−	+	−	+	+	yes	not enough data

Jarvik 2020	98 clusters	548	NR	relative difference: -0.7% (-2.9% to 1.7%)	health care utilization (TV)	Provider education	1) radiology texts contained info on age specific common findings on people without low-back pain	Stepped wedge trial	yes	no	+	+	+	−	+	+	yes	reports only relative reduction
Pagaiya 2005	18 clusters	180	Diazepam - 7.1% vs -2.6 Ab for respiratory infection: - 14.6% vs 2.8%, ab for diarrhea - 1.8% vs - 2.1%	p-value for diazepam 0.029, and for antibiotics in children with acute respiratory infection 0.022 and in diarrhea 0.308	diazepam , antibiotic s (TV)	Provider education + audit and feedback	1) 3-days training seminar<br>2) 2-hours educational meeting,<br>3) One-time feedback, supervisors randomly checked patient chart to give feedback if the care was adequate	Usual care	no	no	+	+	+	+	+	+	yes	not enough data
Foxman 1987	5765 patients	2920	NR	rate ratio: 1.80 (1.75-1.86)	Antibiotic s (TV)	Financial	1) Fee-for-service insurance plans. One group got free care, for other different proportions of costs were paid.	free care and other study groups paid 20-95% of the care expenses	no	no	+	−	−	−	+	+	no	report only relative risk
Ashwort h 2021	296 providers	365	NR	non-significant differences vs control; -44% in control, -50% intervention 1, - 43% intervention 2, and -54% intervention 3	benzodiaz epines (TV)	Provider education + audit and feedback	4 groups<br>1) audit and feedback including peer comparison<br>2) audit and feedback, + one educational letter ("warning letter") prompting to reduce benzodiazepines prescribing<br>3) 2 + call from pharmacist<br>4) 2 + call from physician	group 1 was control	no	no	+	+	+	+	+	−	no	reports only change from baseline

Gold 2022	920 clusters	210	NR	"β < −0.01, z = −0.50, p = 0.565"	Antibiotics (TV)	Provider education + Audit and feedback + Patient education	1) audit and feedback - letter stating, "your practice is in the minority that have increased their prescribing by more than 4%."<br>2) educational material on reducing unnecessary prescribing<br>3) self-help leaflet for patients - participants could distribute	no intervention	no	yes	+	+	+	+	+	+	no	not enough data
Chazan 2007	8 clusters	485	Mean DDD per 1000 patients/day:<br>I: 27.8<br>C:28.7	NR	Antibiotics	Provider education + Patient education	1) continuous interactive educational sessions on recommendations and diagnostic tools<br>2) educational leaflets for patients	One time 2 hours educational meeting including patient leaflets to be distributed	no	no	+	+	+	−	+	+	no	not enough data
Andrade 2022	2552 providers	365	Gapapentinoid prescriptions DDD mean reduction:<br>I: -0.058 (0.38)<br>C: -0.058 (0.37)	p-value 0.98	Gapapentinoids	Audit and feedback + patient education	1) Audit and feedback, postal or email<br>2) Educational material for patients, postal	postal version of audit and feedback, both groups got the educational materials for patients	no	yes	+	+	+	+	−	+	no	not enough data
Lundborg 1999	36 practices, 204 physicians	365	2 outcomes, median:<br>I1: 31 (range 13-93)<br>I2: 17 (8-57)<br>C1: 34 (8-117)<br>C2: 15.5 (3-67)	not reported, control got same intervention on asthma prescribing so, both outcomes were in favor for intervention	Antibiotics and asthma medications (TV)	Provider education + Audit and feedback	1) Two 1,4 hours long educational sessions including educational tasks on written patient cases and feedback on those tasks<br>2) Second session included feedback and discussion on the individual prescribing	Control got on asthma prescribing (outcome 2).	no	no	+	+	+	+	+	+	no	Not enought data

Lagerlov 2000	32 practices, 198 physicians	365	not reported	2 outcomes, relative decrease in unacceptable treatments: O1: -1.4%, p-value 0.48 02: -9.6%, p-value 0.0004 both in favor of intervention	Antibiotics and asthma medications (TV)	Provider education + Audit and feedback	1) Two 2 hours 45min educational meetings<br>2) Second meeting included feedback on prescribing according to quality criteria defined by the participant groups	Same intervention on different topic	no	yes	+	+	+	−	+	no	Not enough data	365
Bocquier 2024	500 physicians	365	Mean:<br>I: 611<br>C: 673	Difference in difference: -2.4 (-27.0 to 22.2)	Antibiotics (TV)	Patient education	1) GPs signed commitment posters displayed in the waiting room<br>2) 2 patient information leaflets given by GPs, one was given if antibiotic prescribed and another if not	No intervention	no	no	+	−	+	+	+	+	no	Not enough data	
Liu 2024	69 practices, 347 physicians	90	Mean proportion(?):<br>I: 52.9%<br>C: 72.5%	p-value under 0.001	other drugs (LVC)	Audit and feedback + Decision aid	1) A pop-up in the EHR when GP prescribes inappropriate glucocorticoids - gives guidance on alternative treatment paths<br>2) Audit and feedback - once GPs access EHR, pop up shows their prescription rates comparison to peers and gives some guidance	Cross-over study	yes	no	+	−	+	+	+	+	no	Not enough data	
McCracken 2023	5157 physicians	180	Mean:<br>I: 1.94<br>C: 1.95	difference in difference: -2.1% (-4.4 to 0.3)	Opioids (TV)	Provider education + Audit and feedback	1) A prescription profile letter of opioids mailed to GP offices once, comparison to peers, educational feedback<br>2) The letter included a link to educational materials	no intervention	yes	no	+	+	+	−	−	+	no	Unclear outcome and no further data from the authors	

Yang 2023	158 clusters, 670 providers	90	Change from baseline:<br>I -13.7%<br>C 1.4%	Rate decrease (transition model):<br>-4% (-7% to 0%)	Antibiotics (TV)	Procider education + Audit and feedback + DA	1) EHR integrated decision aid giving reminders when physicians is prescribing potentially inappropriate antibiotic<br>2) Audit and Feedback, every 10 days, feedback on prescribing rates(?)<br>3) Educational materials	no intervention	no	no	+	−	+	+	+	+	no	Not enough data
Cánovas 2009	8 clusters, 346 patients	180	Mean proportion without antibiotic prescription<br>C: 74.4%<br>I1: 90.2%<br>I2: 45.2%	not reported	Antibiotics (TV)	Audit and feedback	first intervention group (only one center patricipated):<br>1) training on quality assurance methods, not very well described<br>2) Patient case evaluation<br>second group:<br>1) Received results of external quality assessment, including peer centers comparison - likely center-specific information	No intervention	no	no	+	−	+	−	+	+	yes	Only 1 cluster and 2 cluster randomised to 2 groups, not enough data
Nilsson 2001	3 clusters, 40 physicians	365	multiple outcomes	not reported	several drugs	Provider education + Audit and feedback	1) Feedback on individual prescribing rates, discussed on the second educational visit, including peer comparison<br>2) Educational visit for 3 times, 1-1,5 hours each<br>3) Educational material including evidence summaries and recommendations	No intervention	yes	no	+	+	+	−	+	+	yes	Not enough data, Only one cluster per arm
Braybrook 2000	69 clusters	270	Change from baseline:<br>I -1.62 per 1000 patients<br>C -6.27 per 1000 patients	not reported	NSAID	Provider education + Audit and feedback	1) Educational visit to discuss rational NSAID use<br>2) Visit included feedback on NSAID prescribing on practice-specific form and cost information	3) Workbook including prescribing feedback + discussion in their practice	no	no	+	−	+	+	+	+	no	Not enough data

Palen 2019	23 practices	396	Stepped wedge trial: Baseline 10.85 (1.19), Follow-up 11.14 (0.72)	not reported	Imaging	Decision support	1) Imaging orders system asked for indication and suggested alternatives if inappropriate	No interventio n	no	no	+	+	+	−	+	+	yes	Not enough data

Table 13. Evidence certainty assessment

Quality assessment						Summary of findings			
						Effect		Evidence certainty	Plain language summary
No of trials	Risk of bias	Inconsistency	Indirectness	Imprecision	Publication bias	Odds ratio (95% CI)	Absolute effect*		
**Provider education**									
11	Serious limitations	No serious limitations	Serious limitations	Serious limitation	No serious limitations	0.86 (0.72-1.03)	32 (67 fewer to 6 more)	**low**	Provider education may decrease the use of low-value care slightly
**Audit and feedback**									
6	No serious limitations	Serious limitations	No serious limitations	Serious limitations	Not judged due to low number of studies	0.82 (0.67-1.00)	41 (80 to 0) fewer	**Low**	Audit and feedback may decrease low-value care use slightly
**Provider education + Decision support**									
4	Serious limitations	Serious limitations	No serious limitations	Very serious limitations	Not judged due to low number of studies	0.77 (0.34-1.77)	50 (174 fewer to 118 more)	**Very low**	Evidence on Provider education combined with decision aids is highly uncertain
**Audit and feedback + Provider education**									
20	No serious limitations	No serious limitations	Serious limitations	No serious limitations	No serious limitations	0.73 (0.63-0.84)	73 (103 to 42) fewer	**Moderate**	Provider education combined with audit and feedback likely decreases low-value care use slightly
**Patient education**									
4	Serious limitations	No serious limitations	No serious limitations	Serious limitations	Not judged due to low number of studies	0.70 (0.30-1.66)	51 (162 fewer to 68 more)	**Low**	Patient education may decrease low-value care use
**Patient education + Provider education**									
10	Serious limitations	Serious limitations	No serious limitations	No serious limitations	No serious limitations	0.64 (0.50-0.83)	95 (137 to 43) fewer	**Low**	Provider education combined with patient education may decrease low-value care use
**Patient education + Provider education + Decision aid**									
3	Serious limitations	No serious limitations	No serious limitations	Serious limitations	Not judged due to low number of studies	0.61 (0.36-1.04)	100 (179 fewer to 9 more)	**low**	Provider education combined with decision aids and patient education possibly decrease the use of low-value care moderately
**Patient education + Audit and feedback + Provider education**									
5	Serious limitations	No serious limitations	Serious limitations	Serious limitations	Not judged due to low number of studies	0.57 (0.33-1.00)	108 (189 to 0) fewer	**Low**	Provider education combined with patient education and audit and feedback may result in large reduction in low-value care use

*Absolute reduction was estimated by using the median control group risk of getting low-value care

Comments

Provider education: We rated down once due to imprecision and once due to indirectness and risk of bias combined. Six of 11 studies had baseline imbalances and seven of 11 used total volume outcomes. Five of 11 studies had high risk of bias. Low risk and high risk studies had similar results.

Audit and feedback: We rated down for inconsistency and imprecision. Study results were heterogeneous, and confidence intervals included clinically non-meaningful impact.

Provider education + Decision support: We rated down for risk of bias, inconsistency, and imprecision. Three of four trials had high risk of bias. The study with low risk of bias were the only trial suggesting no effect. The confidence intervals included also meaningful harm.

Audit and feedback + Provider education: We rated down due to indirectness. Fifteen of the 20 studies had total volume of care outcome. Furthermore, 10 of 20 trials included educational sessions in their intervention. It is unclear how well the deliverers of educational sessions represent those used outside of the trials which raised concerns about indirectness. There was some inconsistency, but likely mostly explained by the intensity of interventions and randomization unit, individually randomized trials being at risk for contamination. Additionally, in the sensitivity analysis trials with continuous outcomes suggested smaller impact than the primary analysis with all trials (both binary and continuous outcomes). This is also likely explained by the intensity of the interventions, trials using continuous outcomes using lower intensity interventions than trials with binary outcomes. This could be interpreted as a dose-response relationship. We therefore did not rate down for inconsistency.

Patient education: We rated down due to risk of bias and imprecision. Three out of four trials were at high risk of bias. The largest was only a low risk of bias study. We rated down due to imprecision as the pooled estimate included no effect and 95 confidence interval was wide. The largest study control arm had an educational intervention on viral infections (initially considered as being close to the same as no intervention). Excluding the largest study, would have led to a larger pooled effect and more consistent results, OR 0.52 (0.29-0.91). As we did not, however, exclude the study, we did not rate down for inconsistency. Furthermore, we decided not to rate down two levels for imprecision.

Patient education + Provider education: We rated down for inconsistency and risk of bias. Eight from ten used only the total volume of care outcomes. Seven of the ten studies had high risk of bias, although the results were similar between low and high risk of bias studies.

Patient education + Provider education + Decision support: We rated down once due to risk of bias and once for imprecision and indirectness combined. Two of the three trials used total volume outcomes, and confidence intervals included clinically non-meaningful impact. All trials were in high risk of bias.

Patient education + Audit and feedback + Provider education: We rated down once due to imprecision and once due to indirectness and risk of bias combined. All trials used total volume outcomes and Confidence intervals included not clinically meaningful effect. Three of five studies had high risk of bias.

Table 14. Estimated absolute effects (risk difference and 95% confidence interval) for different baseline risks

Intervention and number of studies	With median risk (319 per 1000)	100 per 1000 patients	500 per 1000 patients
Provider education<br>11 trials	32 fewer (67 fewer to 6 more)	10 fewer (21 fewer to 2 more)	50 fewer (105 fewer to 10 more)
Audit and feedback<br>6 trials	41 (80 to 0) fewer	13 (35 to 0) fewer	65 (126 to 0) fewer
Provider education combined with decision support<br>4 trials	50 fewer (174 fewer to 118 more)	16 fewer (55 fewer to 37 more)	78 (273 more to 185 more)
Provider education combined with audit and feedback<br>20 trials	73 (103 to 42) fewer	23 (32 to 13) fewer	115 (161 to 67) fewer
Patient education<br>4 trials	51 fewer (162 fewer to 68 more)	16 fewer (51 fewer to 21 more)	80 fewer (255 fewer to 107 more)
Patient education combined with provider education<br>10 trials	95 (137 to 43) fewer	30 (43 to 13) fewer	149 (215 to 67) fewer
Patient education combined with provider education and decision support<br>3 trials	100 fewer (179 fewer to 9 more)	31 fewer (56 fewer to 3 more)	157 fewer (280 fewer to 14 more)
Patient education combined with audit and feedback and provider education<br>5 trials	108 (185 to 0) fewer	34 (58 to 0) fewer	170 (290 to 0) fewer

Table 15. Studies with appropriate care outcomes

Study	Intervention	Control	Outcome	Reported effect
Sacarny 2018	Audit and feedback	education on another topic	Appropriate care: guideline-concordant quetiapine days per prescriber.	Mean difference -264 (-327 to -201)
Ray 2000	Provider education + Decision aid + patient education	no intervention/usual care	Prescribed acetaminophen days	Difference in difference 10.4 (5.5 to 15.3)
Chien 2017	Other (laboratory system intervention)	no intervention/usual care	Appropriate order rate of Pap tests and colonoscopy per 100 face-to-face encounters	not reported, Means(sd) at follow-up<br>I1: 1.8 (3.6)<br>I2: 2.0 (4.1)<br>C: 1.9 (4.4)
Das 2016	Provider education	No intervention/usual care	proportion of Correct case management. (proportion of patients that got only recommended treatments for diarrhea, asthma, and angina)	OR 1.12 (0.703 to 1.80)
Hurlimann 2015	Provider education + audit and feedback	No intervention/usual care	proportion of Correct case management. (proportion of patients that got only recommended treatments for diarrhea, asthma, and angina)	OR 2.16 (1.19 to 3.91)
Dormuth 2012	Provider education + audit and feedback	no intervention/usual care	proportion of patients that got first prescription of statins for secondary prevention	RR 0.96 (0.91 to 1.01)
Linder 2010	Audit and feedback	no intervention/usual care	Proportion of visit for antibiotic appropriate diagnosis of respiratory track infection that got antibiotic prescription	not reported,<br>I 1718/2624,<br>C 2008/3145

Briel 2006	Provider education	moderate education	Proportion of trimethoprim/sulfamethoxazole prescriptions for uncomplicated lower UTIs over all uncomplicated lower UTIs in adults (≥17 years) treated with antibiotics	1.03 (0.30 to 3.09)
Bhatia 2017	Provider education + audit and feedback + decision aid	no intervention/usual care	The proportion of appropriate transthoracic echocardiogram orders	not reported, Means(sd) at follow-up<br>I: 0.86 (0.13)<br>C: 0.85 (0.10)
Bexell 1996	Provider education	no intervention/usual care	Proportion of patients with correct drug choice	not reported
Coenen 2004	Provider education	provider education	Antibiotics prescribed according to guidelines	1.90 (0.96 to 3.75)
Wächtler 2023	Provider education	no intervention/usual care	Proportion of patients with antibiotic prescription for sore throat that got penisillin V	not reported,<br>I 56/97<br>C 31/68
Dutcher 2022	Provider education + audit and feedback	Stepped wedge trial	Proportion of visits for which antibiotics are almost always indicated that had antibiotic prescription	OR 0.98 (0.83-1.16)
Lagerlov 2000	Provider education + Audit and feedback	Same intervention on different topic	Proportion of acceptably treated patients with urinary tract infection<br><br>Proportion of acceptably treated asthma patients	Relative difference: +13% (variance 2.3)<br><br>+5.9% (variance 2.5)
Carney 2023	Provider education + Audit and feedback	no intervention/usual care	Nitrofurantoin prescriptions for uncomplicated acute cystitis	OR 1.57 (95% CI 1.43 to 1.71)

Fender 2024	Provider education + Decision support	no intervention/usual care	Women with menorrhagia who received tranexamic acid prescription	OR 2.38 (95% CI 1.61 to 3.49)
Martens 2007	Decision support	no intervention/usual care	Appropriate antibiotic prescriptions<br><br>Appropriate asthma/COPD prescriptions<br><br>Appropriate statin prescriptions	rate per 1000 patients:<br>Intervention: 20.7 (95% CI 17.1-26.1)<br>5.9 (3.8-7.9)<br>1.2 (0.7-1.8)<br>Control: 20.5 (14.2-27.4)<br>7.7 (5.6-11.8)<br>1.0 (0.5-2.2)

Table 16. Studies with health and healthcare utilization outcomes

Study	intervention	Control	Outcome	Effect size*
Gulliford 2019	Provider education + Decision aid + audit and feedback + patient education	no intervention/usual care	composite outcome including Pneumonia, Pyelonephritis, Scarlet fever, Peritonsillar abscess, Septic arthritis, Osteomyelitis, Mastoiditis, Meningitis, Empyema, Intracranial abscess, Septicaemia.	Rate ratio 0.92 (0.74 to 1.13)
Jenkins 2013	Patient education + Decision aid	No intervention/usual care	2 outcomes:<br>Emergency department visits and hospitalizations within 30 days	not reported, I 29/2269, C 42/2951 for emergency department visits;<br>I 0/2269 and control group 2/2951 hospitalizations
Butler 2012	Provider education + audit and feedback	no intervention/usual care	2 outcomes:<br>hospital admissions and re-consultation rates for respiratory tract infections	Relative difference 1.9% (-8.2% to 13.2%),<br>median difference -2.32 (-1.95 to 4.76)
Cals 2009	Provider education + audit and feedback	No intervention/usual care	reconsultation within 28 days	not reported,<br>I 55/201,<br>C 85/230
Briel 2006	Provider education	moderate education	re-consultations within 14 days	OR 0.97 (0.78 to 1.21)
Welschen 2004	Provider education + audit and feedback + patient education	no intervention/usual care	Patient satisfaction (scale 1-5)	Mean difference 0.0 (-0.2 to 0.15)
Legare 2012	Provider education + Decision aid	no intervention/usual care	Physical quality of life 2 weeks after the consultation	Mean difference 0.4 (-2.6 to 3.3)
French 2022	Provider education	guideline dissemination	Clinical outcome - low-back pain specific disability (lower numbers better) (RMDQ)	Mean difference 0.37 (-0.48 to 1.21)

Hartman 2023	Provider education + Patient education + Decision aid	no intervention/usual care	All cause mortality - rate per person year	OR 1.08 (0.51 to 2.31)
Cherkin 2018	Provider education + Decision aid	no intervention/usual care	Proportion of patients having additional primary care visits in 6 months follow up	OR 1.31 (0.95 to 1.79)
Jarvik 2020	Provider education	SW	Emergency department visit rate for 6 months after the imaging	OR 0.98 (0.94 to 1.01)
Andrade 2022	Provider education + audit and feedback + patient education	Provider education + audit and feedback + patient education	General practitioner visits during 90 days	p-value 0.04, favoring postal distribution
Simula 2021	Provider education + patient education	no intervention/usual care	Large number of health outcomes: Primary health outcome physical functioning (PROMIS PF-20)	Mean difference 1.0 (-1.5 to 3.5)
Peterson 2023	Provider education + decision support	Provider education	Emergency department or hospital care for women 65 y and over<br><br>emergency department or hospital care for hyperglycemia among previously tightly controlled<br><br>Patients with poor diabetes control among individuals with previously tightly controlled HbA1c	adjusted difference in difference:<br>-0.05 (-0.17 to 0.07)<br><br>-0.45 (-1.38 to 0.11)<br><br>0.47 (0.04 to 1.20)

Reference list – all trials included in the systematic review

1. Aghlmandi S, Halbeisen FS, Saccilotto R, Godet P, Signorell A, Sigrist S, Glinz D, Moffa G, Zeller A, Widmer A F, Kronenberg A, Bielicki J, Bucher HC. Effect of Antibiotic Prescription Audit and Feedback on Antibiotic Prescribing in Primary Care: A Randomized Clinical Trial. JAMA Internal Medicine. 2023. 183:213-220
2. Agnew J, Taaffe M, Darker C, O'Shea B, Clarke J. Delayed prescribing of antibiotics for respiratory tract infections: use of information leaflets. Ir Med J. 2013 Sep;106(8):243-4. PMID: 24282895.
3. Altiner A, Brockmann S, Sielk M, Wilm S, Wegscheider K, Abholz HH. Reducing antibiotic prescriptions for acute cough by motivating GPs to change their attitudes to communication and empowering patients: a cluster-randomized intervention study. J Antimicrob Chemother. 2007 Sep;60(3):638-44. doi: 10.1093/jac/dkm254. Epub 2007 Jul 10. PMID: 17626023.
4. Anderson JF, McEwan KL, Hrudey WP. Effectiveness of notification and group education in modifying prescribing of regulated analgesics. CMAJ. 1996 Jan 1;154(1):31-9. PMID: 8542565; PMCID: PMC1488091.
5. Andrade AQ, Calabretto JP, Pratt NL, Kalisch-Ellett LM, Kassie GM, LeBlanc VT, Ramsay E, Roughead EE. Implementation and Evaluation of a Digitally Enabled Precision Public Health Intervention to Reduce Inappropriate Gabapentinoid Prescription: Cluster Randomized Controlled Trial. J Med Internet Res. 2022 Jan 10;24(1):e33873. doi: 10.2196/33873. PMID: 35006086; PMCID: PMC8787661.
6. Angunawela II, Diwan VK, Tomson G. Experimental evaluation of the effects of drug information on antibiotic prescribing: a study in outpatient care in an area of Sri lanka. Int J Epidemiol. 1991 Jun;20(2):558-64. doi: 10.1093/ije/20.2.558. PMID: 1917265.
7. Ashworth N, Kain N, Wiebe D, Hernandez-Ceron N, Jess E, Mazurek K. Reducing prescribing of benzodiazepines in older adults: a comparison of four physician-focused interventions by a medical regulatory authority. BMC Fam Pract. 2021 Apr 8;22(1):68. doi: 10.1186/s12875-021-01415-x. PMID: 33832432; PMCID: PMC8034172.
8. Avent ML, Hall L, van Driel M, Dobson A, Deckx L, Galal M, Plejdrup Hansen M, Gilks C. Reducing antibiotic prescribing in general practice in Australia: a cluster randomised controlled trial of a multimodal intervention. Aust J Prim Health. 2024 Feb;30(1):NULL. doi: 10.1071/PY23024. PMID: 37844575.
9. Avorn J, Soumerai SB. Improving drug-therapy decisions through educational outreach. A randomized controlled trial of academically based "detailing". N Engl J Med. 1983 Jun 16;308(24):1457-63. doi: 10.1056/NEJM198306163082406. PMID: 6406886.
10. Awad AI, Eltayeb IB, Baraka OZ. Changing antibiotics prescribing practices in health centers of Khartoum State, Sudan. Eur J Clin Pharmacol. 2006 Feb;62(2):135-42. doi: 10.1007/s00228-005-0089-4. Epub 2006 Jan 3. PMID: 16389536.
11. Baker R, Falconer Smith J, Lambert PC. Randomised controlled trial of the effectiveness of feedback in improving test ordering in general practice. Scand J Prim Health Care. 2003 Dec;21(4):219-23. doi: 10.1080/02813430310002995. PMID: 14695072.
12. Behavioural economics team of the Australian Goverment (BETA). Nudge vs Superbugs: a behavioural economics trial to reduce the overprescribing of antibiotics. Canberra: Department of the Prime Minister and Cabinet.
13. Berings D, Blondeel L, Habraken H. The effect of industry-independent drug information on the prescribing of benzodiazepines in general practice. Eur J Clin Pharmacol. 1994;46(6):501-5. doi: 10.1007/BF00196105. PMID: 7995315.
14. Bernal-Delgado E, Galeote-Mayor M, Pradas-Arnal F, Peiró-Moreno S. Evidence based educational outreach visits: effects on prescriptions of non-steroidal anti-inflammatory drugs. J

Epidemiol Community Health. 2002 Sep;56(9):653-8. doi: 10.1136/jech.56.9.653. PMID: 12177080; PMCID: PMC1732253.
15. Bexell A, Lwando E, von Hofsten B, Tembo S, Eriksson B, Diwan VK. Improving drug use through continuing education: a randomized controlled trial in Zambia. J Clin Epidemiol. 1996 Mar;49(3):355-7. doi: 10.1016/0895-4356(95)00059-3. PMID: 8676185.
16. Bhatia RS, Ivers NM, Yin XC, Myers D, Nesbitt GC, Edwards J, Yared K, Wadhera RK, Wu JC, Kithcart AP, Wong BM, Hansen MS, Weinerman AS, Shadowitz S, Elman D, Farkouh ME, Thavendiranathan P, Udell JA, Johri AM, Chow CM, Hall J, Bouck Z, Cohen A, Thorpe KE, Rakowski H, Picard MH, Weiner RB. Improving the Appropriate Use of Transthoracic Echocardiography: The Echo WISELY Trial. J Am Coll Cardiol. 2017 Aug 29;70(9):1135-1144. doi: 10.1016/j.jacc.2017.06.065. PMID: 28838362.
17. Bocquier A, Essilini A, Pereira O, Welter A, Pulcini C, Thilly N; on the behalf of the AntibioCharte Scientific Committee. Impact of a public commitment charter, a non-prescription pad and an antibiotic information leaflet to improve antibiotic prescription among general practitioners: A randomised controlled study. J Infect Public Health. 2024 Feb;17(2):217-225. doi: 10.1016/j.jiph.2023.11.027. Epub 2023 Dec 2. PMID: 38113819.
18. Bonney A, Kobel C, Mullan J, Metusela C, Rhee JJ, Barnett S, Batterham M. Randomised trial of general practitioner online education for prescribing and test ordering. BMJ Open Qual. 2023 Oct;12(4):e002351. doi: 10.1136/bmjoq-2023-002351. PMID: 37857521; PMCID: PMC10603404.
19. Braybrook S, Walker R. Influencing NSAID prescribing in primary care using different feedback strategies. Pharm World Sci. 2000 Apr;22(2):39-46. doi: 10.1023/a:1008790925035. PMID: 10849921.
20. Briel M, Langewitz W, Tschudi P, Young J, Hugenschmidt C, Bucher HC. Communication training and antibiotic use in acute respiratory tract infections. A cluster randomised controlled trial in general practice. Swiss Med Wkly. 2006 Apr 15;136(15-16):241-7. doi: 10.4414/smw.2006.11342. PMID: 16708309.
21. Butler CC, Simpson SA, Dunstan F, Rollnick S, Cohen D, Gillespie D, Evans MR, Alam MF, Bekkers MJ, Evans J, Moore L, Howe R, Hayes J, Hare M, Hood K. Effectiveness of multifaceted educational programme to reduce antibiotic dispensing in primary care: practice based randomised controlled trial. BMJ. 2012 Feb 2;344:d8173. doi: 10.1136/bmj.d8173. PMID: 22302780; PMCID: PMC3270575.
22. Cals JW, Butler CC, Hopstaken RM, Hood K, Dinant GJ. Effect of point of care testing for C reactive protein and training in communication skills on antibiotic use in lower respiratory tract infections: cluster randomised trial. BMJ. 2009 May 5;338:b1374. doi: 10.1136/bmj.b1374. PMID: 19416992; PMCID: PMC2677640.
23. Cánovas JJ, Hernández PJ, Botella JJ. Effectiveness of internal quality assurance programmes in improving clinical practice and reducing costs. J Eval Clin Pract. 2009 Oct;15(5):813-9. doi: 10.1111/j.1365-2753.2008.01100.x. PMID: 19811594.
24. Carney G, Maclure M, Patrick DM, Fisher A, Stanley D, Bassett K, Dormuth CR. A cluster randomized trial assessing the impact of personalized prescribing feedback on antibiotic prescribing for uncomplicated acute cystitis to family physicians. PLoS One. 2023 Jul 31;18(7):e0280096. doi: 10.1371/journal.pone.0280096. PMID: 37523381; PMCID: PMC10389722.
25. Chazan B, Turjeman RB, Frost Y, Besharat B, Tabenkin H, Stainberg A, Sakran W, Raz R. Antibiotic consumption successfully reduced by a community intervention program. Isr Med Assoc J. 2007 Jan;9(1):16-20. Erratum in: Isr Med Assoc J. 2007 Mar;9(3):188. PMID: 17274349.

26. Chien AT, Lehmann LS, Hatfield LA, Koplan KE, Petty CR, Sinaiko AD, Rosenthal MB, Sequist TD. A Randomized Trial of Displaying Paid Price Information on Imaging Study and Procedure Ordering Rates. J Gen Intern Med. 2017 Apr;32(4):434-448. doi: 10.1007/s11606-016-3917-6. Epub 2016 Dec 2. PMID: 27913910; PMCID: PMC5377881.
27. Coenen S, Van Royen P, Michiels B, Denekens J. Optimizing antibiotic prescribing for acute cough in general practice: a cluster-randomized controlled trial. J Antimicrob Chemother. 2004 Sep;54(3):661-72. doi: 10.1093/jac/dkh374. Epub 2004 Jul 28. PMID: 15282232.
28. Cundill B, Mbakilwa H, Chandler CI, Mtove G, Mtei F, Willetts A, Foster E, Muro F, Mwinyishehe R, Mandike R, Olomi R, Whitty CJ, Reyburn H. Prescriber and patient-oriented behavioural interventions to improve use of malaria rapid diagnostic tests in Tanzania: facility-based cluster randomised trial. BMC Med. 2015 May 15;13:118. doi: 10.1186/s12916-015-0346-z. PMID: 25980737; PMCID: PMC4445498.
29. Curtis HJ, Bacon S, Croker R, Walker AJ, Perera R, Hallsworth M, Harper H, Mahtani KR, Heneghan C, Goldacre B. Evaluating the impact of a very low-cost intervention to increase practices' engagement with data and change prescribing behaviour: a randomized trial in English primary care. Fam Pract. 2021 Jul 28;38(4):373-380. doi: 10.1093/fampra/cmaa128. PMID: 33783497.
30. Cherkin D, Balderson B, Wellman R, Hsu C, Sherman KJ, Evers SC, Hawkes R, Cook A, Levine MD, Piekara D, Rock P, Estlin KT, Brewer G, Jensen M, LaPorte AM, Yeoman J, Sowden G, Hill JC, Foster NE. Effect of Low Back Pain Risk-Stratification Strategy on Patient Outcomes and Care Processes: the MATCH Randomized Trial in Primary Care. J Gen Intern Med. 2018 Aug;33(8):1324-1336. doi: 10.1007/s11606-018-4468-9. Epub 2018 May 22. PMID: 29790073; PMCID: PMC6082187.
31. Das J, Chowdhury A, Hussam R, Banerjee AV. The impact of training informal health care providers in India: A randomized controlled trial. Science. 2016 Oct 7;354(6308):aaf7384. doi: 10.1126/science.aaf7384. PMID: 27846471.
32. de Burgh S, Mant A, Mattick RP, Donnelly N, Hall W, Bridges-Webb C. A controlled trial of educational visiting to improve benzodiazepine prescribing in general practice. Aust J Public Health. 1995 Apr;19(2):142-8. doi: 10.1111/j.1753-6405.1995.tb00364.x. PMID: 7786939.
33. Dey P, Simpson CW, Collins SI, Hodgson G, Dowrick CF, Simison AJ, Rose MJ. Implementation of RCGP guidelines for acute low back pain: a cluster randomised controlled trial. Br J Gen Pract. 2004 Jan;54(498):33-7. PMID: 14965404; PMCID: PMC1314775.
34. Dormuth CR, Carney G, Taylor S, Bassett K, Maclure M. A randomized trial assessing the impact of a personal printed feedback portrait on statin prescribing in primary care. J Contin Educ Health Prof. 2012 Summer;32(3):153-62. doi: 10.1002/chp.21140. PMID: 23008077.
35. Du Yan L, Dean K, Park D, Thompson J, Tong I, Liu C, Hamdy RF. Education vs Clinician Feedback on Antibiotic Prescriptions for Acute Respiratory Infections in Telemedicine: a Randomized Controlled Trial. J Gen Intern Med. 2021 Feb;36(2):305-312. doi: 10.1007/s11606-020-06134-0. Epub 2020 Aug 26. PMID: 32845446; PMCID: PMC7878643.
36. Dutcher L, Degnan K, Adu-Gyamfi AB, Lautenbach E, Cressman L, David MZ, Cluzet V, Szymczak JE, Pegues DA, Bilker W, Tolomeo P, Hamilton KW. Improving Outpatient Antibiotic Prescribing for Respiratory Tract Infections in Primary Care: A Stepped-Wedge Cluster Randomized Trial. Clin Infect Dis. 2022 Mar 23;74(6):947-956. doi: 10.1093/cid/ciab602. PMID: 34212177; PMCID: PMC9630878.
37. Eccles M, Steen N, Grimshaw J, Thomas L, McNamee P, Soutter J, Wilsdon J, Matowe L, Needham G, Gilbert F, Bond S. Effect of audit and feedback, and reminder messages on primary-care radiology referrals: a randomised trial. Lancet. 2001 May 5;357(9266):1406-9. doi: 10.1016/S0140-6736(00)04564-5. PMID: 11356439.

38. Eltayeb IB, Awad AI, Mohamed-Salih MS, Daffa-Alla MA, Ahmed MB, Ogail MA, Matowe L. Changing the prescribing patterns of sexually transmitted infections in the White Nile Region of Sudan. Sex Transm Infect. 2005 Oct;81(5):426-7. doi: 10.1136/sti.2004.014001. PMID: 16199745; PMCID: PMC1745049.
39. Feldmeier G, Löffler C, Altiner A, Wollny A, Garbe K, Kronsteiner D, Köppen M, Szecsenyi J, Leyh M, Voss A, Kamradt M, Poß-Doering R, Wensing M, Kaufmann-Kolle P. Optimizing Antibiotic Prescribing for Acute Respiratory Tract Infections in German Primary Care: Results of the Regional Intervention Study CHANGE-3 and the Nested cRCT. Antibiotics (Basel). 2023 May 4;12(5):850. doi: 10.3390/antibiotics12050850. PMID: 37237753; PMCID: PMC10215067.
40. Fenton JJ, Kravitz RL, Jerant A, Paterniti DA, Bang H, Williams D, Epstein RM, Franks P. Promoting Patient-Centered Counseling to Reduce Use of Low-Value Diagnostic Tests: A Randomized Clinical Trial. JAMA Intern Med. 2016 Feb;176(2):191-7. doi: 10.1001/jamainternmed.2015.6840. PMID: 26640973.
41. Flottorp S, Oxman AD, Håvelsrud K, Treweek S, Herrin J. Cluster randomised controlled trial of tailored interventions to improve the management of urinary tract infections in women and sore throat. BMJ. 2002 Aug 17;325(7360):367. doi: 10.1136/bmj.325.7360.367. PMID: 12183309; PMCID: PMC117890.
42. Foxman B, Valdez RB, Lohr KN, Goldberg GA, Newhouse JP, Brook RH. The effect of cost sharing on the use of antibiotics in ambulatory care: results from a population-based randomized controlled trial. J Chronic Dis. 1987;40(5):429-37. doi: 10.1016/0021-9681(87)90176-7. PMID: 3104386.
43. French SD, O'Connor DA, Green SE, Page MJ, Mortimer DS, Turner SL, Walker BF, Keating JL, Grimshaw JM, Michie S, Francis JJ, McKenzie JE. Improving adherence to acute low back pain guideline recommendations with chiropractors and physiotherapists: the ALIGN cluster randomised controlled trial. Trials. 2022 Feb 14;23(1):142. doi: 10.1186/s13063-022-06053-x. PMID: 35164841; PMCID: PMC8842895.
44. French SD, McKenzie JE, O'Connor DA, Grimshaw JM, Mortimer D, Francis JJ, Michie S, Spike N, Schattner P, Kent P, Buchbinder R, Page MJ, Green SE. Evaluation of a theory-informed implementation intervention for the management of acute low back pain in general medical practice: the IMPLEMENT cluster randomised trial. PLoS One. 2013 Jun 13;8(6):e65471. doi: 10.1371/journal.pone.0065471. PMID: 23785427; PMCID: PMC3681882.
45. Gjelstad S, Høye S, Straand J, Brekke M, Dalen I, Lindbæk M. Improving antibiotic prescribing in acute respiratory tract infections: cluster randomised trial from Norwegian general practice (prescription peer academic detailing (Rx-PAD) study). BMJ. 2013 Jul 26;347:f4403. doi: 10.1136/bmj.f4403. PMID: 23894178; PMCID: PMC3724398.
46. Gold, N., Ratajczak, M., Sallis, A. *et al.* Provision of social-norms feedback to general practices whose antibiotic prescribing is increasing: a national randomized controlled trial. *J Public Health (Berl.)* **30**, 2351–2358 (2022). https://doi.org/10.1007/s10389-021-01645-4
47. Goldberg HI, Deyo RA, Taylor VM, Cheadle AD, Conrad DA, Loeser JD, Heagerty PJ, Diehr P. Can evidence change the rate of back surgery? A randomized trial of community-based education. Eff Clin Pract. 2001 May-Jun;4(3):95-104. PMID: 11434080.
48. Gonzales R, Anderer T, McCulloch CE, Maselli JH, Bloom FJ Jr, Graf TR, Stahl M, Yefko M, Molecavage J, Metlay JP. A cluster randomized trial of decision support strategies for reducing antibiotic use in acute bronchitis. JAMA Intern Med. 2013 Feb 25;173(4):267-73. doi: 10.1001/jamainternmed.2013.1589. PMID: 23319069; PMCID: PMC3582762.
49. Fender GR, Prentice A, Gorst T, Nixon RM, Duffy SW, Day NE, Smith SK. Randomised controlled trial of educational package on management of menorrhagia in primary care: the Anglia

menorrhagia education study. BMJ. 1999 May 8;318(7193):1246-50. doi: 10.1136/bmj.318.7193.1246. PMID: 10231255; PMCID: PMC27863.
50. Gulliford MC, Prevost AT, Charlton J, Juszczyk D, Soames J, McDermott L, Sultana K, Wright M, Fox R, Hay AD, Little P, Moore MV, Yardley L, Ashworth M. Effectiveness and safety of electronically delivered prescribing feedback and decision support on antibiotic use for respiratory illness in primary care: REDUCE cluster randomised trial. BMJ. 2019 Feb 12;364:l236. doi: 10.1136/bmj.l236. PMID: 30755451; PMCID: PMC6371944.
51. Gulliford MC, van Staa T, Dregan A, McDermott L, McCann G, Ashworth M, Charlton J, Little P, Moore MV, Yardley L. Electronic health records for intervention research: a cluster randomized trial to reduce antibiotic prescribing in primary care (eCRT study). Ann Fam Med. 2014 Jul;12(4):344-51. doi: 10.1370/afm.1659. PMID: 25024243; PMCID: PMC4096472.
52. Hadiyono JE, Suryawati S, Danu SS, Sunartono, Santoso B. Interactional group discussion: results of a controlled trial using a behavioral intervention to reduce the use of injections in public health facilities. Soc Sci Med. 1996 Apr;42(8):1177-83. doi: 10.1016/0277-9536(95)00391-6. PMID: 8737436.
53. Hallsworth M, Chadborn T, Sallis A, Sanders M, Berry D, Greaves F, Clements L, Davies SC. Provision of social norm feedback to high prescribers of antibiotics in general practice: a pragmatic national randomised controlled trial. Lancet. 2016 Apr 23;387(10029):1743-52. doi: 10.1016/S0140-6736(16)00215-4. Epub 2016 Feb 18. PMID: 26898856; PMCID: PMC4842844.
54. Hamilton W, Russell D, Stabb C, Seamark D, Campion-Smith C, Britten N. The effect of patient self-completion agenda forms on prescribing and adherence in general practice: a randomized controlled trial. Fam Pract. 2007 Feb;24(1):77-83. doi: 10.1093/fampra/cml057. Epub 2006 Nov 30. PMID: 17142247.
55. Hartman EAR, van de Pol AC, Heltveit-Olsen SR, Lindbæk M, Høye S, Lithén SS, Sundvall PD, Sundvall S, Arnljots ES, Gunnarsson R, Kowalczyk A, Godycki-Cwirko M, Platteel TN, Groen WG, Monnier AA, Zuithoff NP, Verheij TJM, Hertogh CMPM. Effect of a multifaceted antibiotic stewardship intervention to improve antibiotic prescribing for suspected urinary tract infections in frail older adults (ImpresU): pragmatic cluster randomised controlled trial in four European countries. BMJ. 2023 Feb 22;380:e072319. doi: 10.1136/bmj-2022-072319. PMID: 36813284; PMCID: PMC9943914.
56. Hemkens LG, Saccilotto R, Reyes SL, Glinz D, Zumbrunn T, Grolimund O, Gloy V, Raatz H, Widmer A, Zeller A, Bucher HC. Personalized Prescription Feedback Using Routinely Collected Data to Reduce Antibiotic Use in Primary Care: A Randomized Clinical Trial. JAMA Intern Med. 2017 Feb 1;177(2):176-183. doi: 10.1001/jamainternmed.2016.8040. PMID: 28027333.
57. Hemminki E, Heikkilä K, Sevón T, Koponen P. Special features of health services and register based trials - experiences from a randomized trial of childbirth classes. BMC Health Serv Res. 2008 Jun 11;8:126. doi: 10.1186/1472-6963-8-126. PMID: 18547413; PMCID: PMC2442595.
58. Hürlimann D, Limacher A, Schabel M, Zanetti G, Berger C, Mühlemann K, Kronenberg A; Swiss Sentinel Working Group. Improvement of antibiotic prescription in outpatient care: a cluster-randomized intervention study using a sentinel surveillance network of physicians. J Antimicrob Chemother. 2015 Feb;70(2):602-8. doi: 10.1093/jac/dku394. Epub 2014 Oct 17. PMID: 25326088.
59. Ilett KF, Johnson S, Greenhill G, Mullen L, Brockis J, Golledge CL, Reid DB. Modification of general practitioner prescribing of antibiotics by use of a therapeutics adviser (academic detailer). Br J Clin Pharmacol. 2000 Feb;49(2):168-73. doi: 10.1046/j.1365-2125.2000.00123.x. PMID: 10671912; PMCID: PMC2014897.
60. Jarvik JG, Meier EN, James KT, Gold LS, Tan KW, Kessler LG, Suri P, Kallmes DF, Cherkin DC, Deyo RA, Sherman KJ, Halabi SS, Comstock BA, Luetmer PH, Avins AL, Rundell SD, Griffith B, Friedly JL,

Lavallee DC, Stephens KA, Turner JA, Bresnahan BW, Heagerty PJ. The Effect of Including Benchmark Prevalence Data of Common Imaging Findings in Spine Image Reports on Health Care Utilization Among Adults Undergoing Spine Imaging: A Stepped-Wedge Randomized Clinical Trial. JAMA Netw Open. 2020 Sep 1;3(9):e2015713. doi: 10.1001/jamanetworkopen.2020.15713. PMID: 32886121; PMCID: PMC7489827.

61. Jenkins TC, Irwin A, Coombs L, Dealleaume L, Ross SE, Rozwadowski J, Webster B, Dickinson LM, Sabel AL, Mackenzie TD, West DR, Price CS. Effects of clinical pathways for common outpatient infections on antibiotic prescribing. Am J Med. 2013 Apr;126(4):327-335.e12. doi: 10.1016/j.amjmed.2012.10.027. PMID: 23507206; PMCID: PMC3666348.
62. Kerfoot BP, Lawler EV, Sokolovskaya G, Gagnon D, Conlin PR. Durable improvements in prostate cancer screening from online spaced education a randomized controlled trial. Am J Prev Med. 2010 Nov;39(5):472-8. doi: 10.1016/j.amepre.2010.07.016. PMID: 20965387; PMCID: PMC2994103.
63. Kerry S, Oakeshott P, Dundas D, Williams J. Influence of postal distribution of the Royal College of Radiologists' guidelines, together with feedback on radiological referral rates, on X-ray referrals from general practice: a randomized controlled trial. Fam Pract. 2000 Feb;17(1):46-52. doi: 10.1093/fampra/17.1.46. PMID: 10673488.
64. Kullgren JT, Kim HM, Slowey M, Colbert J, Soyster B, Winston SA, Ryan K, Forman JH, Riba M, Krupka E, Kerr EA. Using Behavioral Economics to Reduce Low-Value Care Among Older Adults: A Cluster Randomized Clinical Trial. JAMA Intern Med. 2024 Mar 1;184(3):281-290. doi: 10.1001/jamainternmed.2023.7703. PMID: 38285565; PMCID: PMC10825788.
65. Kullgren JT, Krupka E, Schachter A, Linden A, Miller J, Acharya Y, Alford J, Duffy R, Adler-Milstein J. Precommitting to choose wisely about low-value services: a stepped wedge cluster randomised trial. BMJ Qual Saf. 2018 May;27(5):355-364. doi: 10.1136/bmjqs-2017-006699. Epub 2017 Oct 24. PMID: 29066616.
66. Lagerløv P, Loeb M, Andrew M, Hjortdahl P. Improving doctors' prescribing behaviour through reflection on guidelines and prescription feedback: a randomised controlled study. Qual Health Care. 2000 Sep;9(3):159-65. doi: 10.1136/qhc.9.3.159. PMID: 10980076; PMCID: PMC1743532.
67. Lee MHM, Pan DST, Huang JH, Chen MI, Chong JWC, Goh EH, Jiang L, Leo YS, Lee TH, Wong CS, Loh VWK, Lim FS, Poh AZ, Tham TY, Wong WM, Yu Y. Results from a Patient-Based Health Education Intervention in Reducing Antibiotic Use for Acute Upper Respiratory Tract Infections in the Private Sector Primary Care Setting in Singapore. Antimicrob Agents Chemother. 2017 Apr 24;61(5):e02257-16. doi: 10.1128/AAC.02257-16. PMID: 28193663; PMCID: PMC5404603.
68. Légaré F, Labrecque M, Cauchon M, Castel J, Turcotte S, Grimshaw J. Training family physicians in shared decision-making to reduce the overuse of antibiotics in acute respiratory infections: a cluster randomized trial. CMAJ. 2012 Sep 18;184(13):E726-34. doi: 10.1503/cmaj.120568. Epub 2012 Jul 30. PMID: 22847969; PMCID: PMC3447039.
69. Linder JA, Schnipper JL, Tsurikova R, Yu DT, Volk LA, Melnikas AJ, Palchuk MB, Olsha-Yehiav M, Middleton B. Electronic health record feedback to improve antibiotic prescribing for acute respiratory infections. Am J Manag Care. 2010 Dec;16(12 Suppl HIT):e311-9. PMID: 21322301.
70. Linder JA, Schnipper JL, Tsurikova R, Yu T, Volk LA, Melnikas AJ, Palchuk MB, Olsha-Yehiav M, Middleton B. Documentation-based clinical decision support to improve antibiotic prescribing for acute respiratory infections in primary care: a cluster randomised controlled trial. Inform Prim Care. 2009;17(4):231-40. doi: 10.14236/jhi.v17i4.742. PMID: 20359401.
71. Little P, Stuart B, Francis N, Douglas E, Tonkin-Crine S, Anthierens S, Cals JW, Melbye H, Santer M, Moore M, Coenen S, Butler C, Hood K, Kelly M, Godycki-Cwirko M, Mierzecki A, Torres A, Llor C, Davies M, Mullee M, O'Reilly G, van der Velden A, Geraghty AW, Goossens H, Verheij T, Yardley

L; GRACE consortium. Effects of internet-based training on antibiotic prescribing rates for acute respiratory-tract infections: a multinational, cluster, randomised, factorial, controlled trial. Lancet. 2013 Oct 5;382(9899):1175-82. doi: 10.1016/S0140-6736(13)60994-0. Epub 2013 Jul 31. PMID: 23915885; PMCID: PMC3807804.
72. Liu L, Wang L, Zhou H, Yang J, Wang W, Luo X, Chang Y. Feedback Intervention for the Control of Glucocorticoid Prescription in Primary Care Institutions: A Cluster Randomized Cross-Over Controlled Trial in Southwest China. Risk Manag Healthc Policy. 2024 Jan 5;17:49-63. doi: 10.2147/RMHP.S441165. PMID: 38196917; PMCID: PMC10775693.
73. Llor C, Trapero-Bertran M, Sisó-Almirall A, Monfà R, Abellana R, García-Sangenís A, Moragas A, Morros R. Effects of C-reactive protein rapid testing and communication skills training on antibiotic prescribing for acute cough. A cluster factorial randomised controlled trial. NPJ Prim Care Respir Med. 2024 May 9;34(1):9. doi: 10.1038/s41533-024-00368-9. PMID: 38724543; PMCID: PMC11081949.
74. Lundborg CS, Wahlström R, Oke T, Tomson G, Diwan VK. Influencing prescribing for urinary tract infection and asthma in primary care in Sweden: a randomized controlled trial of an interactive educational intervention. J Clin Epidemiol. 1999 Aug;52(8):801-12. doi: 10.1016/s0895-4356(99)00036-0. PMID: 10465325.
75. Macfarlane J, Holmes W, Gard P, Thornhill D, Macfarlane R, Hubbard R. Reducing antibiotic use for acute bronchitis in primary care: blinded, randomised controlled trial of patient information leaflet. BMJ. 2002 Jan 12;324(7329):91-4. doi: 10.1136/bmj.324.7329.91. PMID: 11786454; PMCID: PMC64506.
76. Mann D, Hess R, McGinn T, Richardson S, Jones S, Palmisano J, Chokshi SK, Mishuris R, McCullagh L, Park L, Dinh-Le C, Smith P, Feldstein D. Impact of Clinical Decision Support on Antibiotic Prescribing for Acute Respiratory Infections: a Cluster Randomized Implementation Trial. J Gen Intern Med. 2020 Nov;35(Suppl 2):788-795. doi: 10.1007/s11606-020-06096-3. Epub 2020 Sep 1. PMID: 32875505; PMCID: PMC7652959.
77. Martens JD, van der Weijden T, Severens JL, de Clercq PA, de Bruijn DP, Kester AD, Winkens RA. The effect of computer reminders on GPs' prescribing behaviour: a cluster-randomised trial. Int J Med Inform. 2007 Dec;76 Suppl 3:S403-16. doi: 10.1016/j.ijmedinf.2007.04.005. Epub 2007 Jun 14. PMID: 17569575.
78. Martins CM, da Costa Teixeira AS, de Azevedo LF, Sá LM, Santos PA, do Couto ML, da Costa Pereira AM, Hespanhol AA, da Costa Santos CM. The effect of a test ordering software intervention on the prescription of unnecessary laboratory tests - a randomized controlled trial. BMC Med Inform Decis Mak. 2017 Feb 20;17(1):20. doi: 10.1186/s12911-017-0416-6. PMID: 28219437; PMCID: PMC5319139.
79. McCracken RK, Narayan S, Maclure M, Cooper I, Cui Z, Cullen W, Dormuth C, Hamilton MA, Nolan S, Singer J, Socías ME, Wong S, Klimas J. Evaluation of audit and feedback to family physicians on prescribing of opioid analgesics to opioid-naïve patients: A pragmatic randomized delay trial. Contemp Clin Trials. 2023 Nov;134:107354. doi: 10.1016/j.cct.2023.107354. Epub 2023 Oct 5. PMID: 37802223.
80. McGinn TG, McCullagh L, Kannry J, Knaus M, Sofianou A, Wisnivesky JP, Mann DM. Efficacy of an evidence-based clinical decision support in primary care practices: a randomized clinical trial. JAMA Intern Med. 2013 Sep 23;173(17):1584-91. doi: 10.1001/jamainternmed.2013.8980. PMID: 23896675.
81. McIsaac WJ, Goel V. Effect of an explicit decision-support tool on decisions to prescribe antibiotics for sore throat. Med Decis Making. 1998 Apr-Jun;18(2):220-8. doi: 10.1177/0272989X9801800211. PMID: 9566455.

82. McIsaac WJ, Goel V, To T, Permaul JA, Low DE. Effect on antibiotic prescribing of repeated clinical prompts to use a sore throat score: lessons from a failed community intervention study. J Fam Pract. 2002 Apr;51(4):339-44. PMID: 11978257.
83. McNulty C, Hawking M, Lecky D, Jones L, Owens R, Charlett A, Butler C, Moore P, Francis N. Effects of primary care antimicrobial stewardship outreach on antibiotic use by general practice staff: pragmatic randomized controlled trial of the TARGET antibiotics workshop. J Antimicrob Chemother. 2018 May 1;73(5):1423-1432. doi: 10.1093/jac/dky004. PMID: 29514268; PMCID: PMC5909634.
84. Meeker D, Knight TK, Friedberg MW, Linder JA, Goldstein NJ, Fox CR, Rothfeld A, Diaz G, Doctor JN. Nudging guideline-concordant antibiotic prescribing: a randomized clinical trial. JAMA Intern Med. 2014 Mar;174(3):425-31. doi: 10.1001/jamainternmed.2013.14191. PMID: 24474434; PMCID: PMC4648560.
85. Meeker D, Linder JA, Fox CR, Friedberg MW, Persell SD, Goldstein NJ, Knight TK, Hay JW, Doctor JN. Effect of Behavioral Interventions on Inappropriate Antibiotic Prescribing Among Primary Care Practices: A Randomized Clinical Trial. JAMA. 2016 Feb 9;315(6):562-70. doi: 10.1001/jama.2016.0275. PMID: 26864410; PMCID: PMC6689234.
86. Midlöv P, Bondesson A, Eriksson T, Nerbrand C, Höglund P. Effects of educational outreach visits on prescribing of benzodiazepines and antipsychotic drugs to elderly patients in primary health care in southern Sweden. Fam Pract. 2006 Feb;23(1):60-4. doi: 10.1093/fampra/cmi105. Epub 2005 Dec 6. PMID: 16332945.
87. Milos V, Jakobsson U, Westerlund T, Melander E, Mölstad S, Midlöv P. Theory-based interventions to reduce prescription of antibiotics--a randomized controlled trial in Sweden. Fam Pract. 2013 Dec;30(6):634-40. doi: 10.1093/fampra/cmt043. Epub 2013 Aug 19. PMID: 23960104.
88. Molina López T, Domínguez Camacho JC, Santos Lozano JM, Carbonell Carrillo A, Sánchez Acevedo J, Paz León ML. Eficacia de las sesiones educativas para modificar la prescripción de fármacos nuevos [Efficacy of educational sessions to modify the prescription of new drugs]. Aten Primaria. 2005 Oct 31;36(7):367-72. Spanish. doi: 10.1157/13080299. PMID: 16266650; PMCID: PMC7669177.
89. Naughton C, Feely J, Bennett K. A RCT evaluating the effectiveness and cost-effectiveness of academic detailing versus postal prescribing feedback in changing GP antibiotic prescribing. J Eval Clin Pract. 2009 Oct;15(5):807-12. doi: 10.1111/j.1365-2753.2008.01099.x. PMID: 19811593.
90. Nejad AS, Noori MR, Haghdoost AA, Bahaadinbeigy K, Abu-Hanna A, Eslami S. The effect of registry-based performance feedback via short text messages and traditional postal letters on prescribing parenteral steroids by general practitioners--A randomized controlled trial. Int J Med Inform. 2016 Mar;87:36-43. doi: 10.1016/j.ijmedinf.2015.12.008. Epub 2015 Dec 19. PMID: 26806710.
91. Nilsson G, Hjemdahl P, Hässler A, Vitols S, Wallén NH, Krakau I. Feedback on prescribing rate combined with problem-oriented pharmacotherapy education as a model to improve prescribing behaviour among general practitioners. Eur J Clin Pharmacol. 2001 Jan-Feb;56(11):843-8. doi: 10.1007/s002280000242. PMID: 11294376.
92. Oakeshott P, Kerry SM, Williams JE. Randomized controlled trial of the effect of the Royal College of Radiologists' guidelines on general practitioners' referrals for radiographic examination. Br J Gen Pract. 1994 May;44(382):197-200. PMID: 8204331; PMCID: PMC1238864.
93. O'Connell DL, Henry D, Tomlins R. Randomised controlled trial of effect of feedback on general practitioners' prescribing in Australia. BMJ. 1999 Feb 20;318(7182):507-11. doi: 10.1136/bmj.318.7182.507. PMID: 10024260; PMCID: PMC27749.

94. Pagaiya N, Garner P. Primary care nurses using guidelines in Thailand: a randomized controlled trial. Trop Med Int Health. 2005 May;10(5):471-7. doi: 10.1111/j.1365-3156.2005.01404.x. PMID: 15860094.
95. Palen TE, Sharpe RE Jr, Shetterly SM, Steiner JF. Randomized Clinical Trial of a Clinical Decision Support Tool for Improving the Appropriateness Scores for Ordering Imaging Studies in Primary and Specialty Care Ambulatory Clinics. AJR Am J Roentgenol. 2019 Nov;213(5):1015-1020. doi: 10.2214/AJR.19.21511. Epub 2019 Jul 16. PMID: 31310183.
96. Persell SD, Petito LC, Lee JY, Meeker D, Doctor JN, Goldstein NJ, Fox CR, Rowe TA, Linder JA, Chmiel R, Peprah YA, Brown T. Reducing Care Overuse in Older Patients Using Professional Norms and Accountability : A Cluster Randomized Controlled Trial. Ann Intern Med. 2024 Mar;177(3):324-334. doi: 10.7326/M23-2183. Epub 2024 Feb 6. Erratum in: Ann Intern Med. 2024 May;177(5):692. doi: 10.7326/L24-0117. PMID: 38315997.
97. Peterson GM, Radford J, Russell G, Zwar N, Mullan J, Batterham M, Mazza D, Eckermann S, Metusela C, Saunder T, Kitsos A, Bonney A. Cluster-randomised trial of the Effectiveness of Quality Incentive Payments in General Practice (EQuIP-GP): Prescribing of medicines outcomes. Res Social Adm Pharm. 2023 May;19(5):836-840. doi: 10.1016/j.sapharm.2023.01.011. Epub 2023 Jan 29. PMID: 36754667.
98. Phuong HL, Nga TT, Giao PT, Hung le Q, Binh TQ, Nam NV, Nagelkerke N, de Vries PJ. Randomised primary health center based interventions to improve the diagnosis and treatment of undifferentiated fever and dengue in Vietnam. BMC Health Serv Res. 2010 Sep 21;10:275. doi: 10.1186/1472-6963-10-275. PMID: 20858230; PMCID: PMC2955016.
99. Pimlott NJ, Hux JE, Wilson LM, Kahan M, Li C, Rosser WW. Educating physicians to reduce benzodiazepine use by elderly patients: a randomized controlled trial. CMAJ. 2003 Apr 1;168(7):835-9. PMID: 12668540; PMCID: PMC151988.
100. Pinto D, Heleno B, Rodrigues DS, Papoila AL, Santos I, Caetano PA. Effectiveness of educational outreach visits compared with usual guideline dissemination to improve family physician prescribing-an 18-month open cluster-randomized trial. Implement Sci. 2018 Sep 5;13(1):120. doi: 10.1186/s13012-018-0810-1. PMID: 30185197; PMCID: PMC6126017.
101. Poss-Doering R, Kronsteiner D, Kamradt M, Kaufmann-Kolle P, Andres E, Wambach V, Bleek J, Wensing M, ARena-Study Group, Szecsenyi J. Assessing Reduction of Antibiotic Prescribing for Acute, Non-Complicated Infections in Primary Care in Germany: Multi-Step Outcome Evaluation in the Cluster-Randomized Trial ARena. Antibiotics (Basel). 2021 Sep 24;10(10):1151. doi: 10.3390/antibiotics10101151. PMID: 34680732; PMCID: PMC8532997.
102. Raebel MA, Charles J, Dugan J, Carroll NM, Korner EJ, Brand DW, Magid DJ. Randomized trial to improve prescribing safety in ambulatory elderly patients. J Am Geriatr Soc. 2007 Jul;55(7):977-85. doi: 10.1111/j.1532-5415.2007.01202.x. PMID: 17608868.
103. Ray WA, Stein CM, Byrd V, Shorr R, Pichert JW, Gideon P, Arnold K, Brandt KD, Pincus T, Griffin MR. Educational program for physicians to reduce use of non-steroidal anti-inflammatory drugs among community-dwelling elderly persons: a randomized controlled trial. Med Care. 2001 May;39(5):425-35. doi: 10.1097/00005650-200105000-00003. PMID: 11317091.
104. Rognstad S, Brekke M, Fetveit A, Dalen I, Straand J. Prescription peer academic detailing to reduce inappropriate prescribing for older patients: a cluster randomised controlled trial. Br J Gen Pract. 2013 Aug;63(613):e554-62. doi: 10.3399/bjgp13X670688. PMID: 23972196; PMCID: PMC3722832.
105. Ruangkanchanasetr S. Laboratory investigation utilization in pediatric out-patient department Ramathibodi Hospital. J Med Assoc Thai. 1993 Oct;76 Suppl 2:194-208. PMID: 7822993.

106.Sacarny A, Barnett ML, Le J, Tetkoski F, Yokum D, Agrawal S. Effect of Peer Comparison Letters for High-Volume Primary Care Prescribers of Quetiapine in Older and Disabled Adults: A Randomized Clinical Trial. JAMA Psychiatry. 2018 Oct 1;75(10):1003-1011. doi: 10.1001/jamapsychiatry.2018.1867. PMID: 30073273; PMCID: PMC6233799.
107.Sacarny A, Yokum D, Finkelstein A, Agrawal S. Medicare Letters To Curb Overprescribing Of Controlled Substances Had No Detectable Effect On Providers. Health Aff (Millwood). 2016 Mar;35(3):471-9. doi: 10.1377/hlthaff.2015.1025. PMID: 26953302.
108.Sallis A, Bondaronek P, Sanders JG, Yu LM, Harris V, Vlaev I, Sanders M, Tonkin-Crine S, Chadborn T. Prescriber Commitment Posters to Increase Prudent Antibiotic Prescribing in English General Practice: A Cluster Randomized Controlled Trial. Antibiotics (Basel). 2020 Aug 7;9(8):490. doi: 10.3390/antibiotics9080490. PMID: 32784625; PMCID: PMC7569839.
109.Samore MH, Bateman K, Alder SC, Hannah E, Donnelly S, Stoddard GJ, Haddadin B, Rubin MA, Williamson J, Stults B, Rupper R, Stevenson K. Clinical decision support and appropriateness of antimicrobial prescribing: a randomized trial. JAMA. 2005 Nov 9;294(18):2305-14. doi: 10.1001/jama.294.18.2305. PMID: 16278358.
110.Schectman JM, Schroth WS, Verme D, Voss JD. Randomized controlled trial of education and feedback for implementation of guidelines for acute low back pain. J Gen Intern Med. 2003 Oct;18(10):773-80. doi: 10.1046/j.1525-1497.2003.10205.x. PMID: 14521638; PMCID: PMC1494929.
111.Schmiemann G, Greser A, Maun A, Bleidorn J, Schuster A, Miljukov O, Rücker V, Klingeberg A, Mentzel A, Minin V, Eckmanns T, Heintze C, Heuschmann P, Gágyor I. Effects of a multimodal intervention in primary care to reduce second line antibiotic prescriptions for urinary tract infections in women: parallel, cluster randomised, controlled trial. BMJ. 2023 Nov 2;383:e076305. doi: 10.1136/bmj-2023-076305. PMID: 37918836; PMCID: PMC10620739.
112.Schwartz KL, Shuldiner J, Langford BJ, Brown KA, Schultz SE, Leung V, Daneman N, Tadrous M, Witteman HO, Garber G, Grimshaw JM, Leis JA, Presseau J, Silverman MS, Taljaard M, Gomes T, Lacroix M, Brehaut J, Thavorn K, Gushue S, Friedman L, Zwarenstein M, Ivers N. Mailed feedback to primary care physicians on antibiotic prescribing for patients aged 65 years and older: pragmatic, factorial randomised controlled trial. BMJ. 2024 Jun 5;385:e079329. doi: 10.1136/bmj-2024-079329. Erratum in: BMJ. 2024 Jun 12;385:q1261. doi: 10.1136/bmj.q1261. PMID: 38839101; PMCID: PMC11151833.
113.Seager JM, Howell-Jones RS, Dunstan FD, Lewis MA, Richmond S, Thomas DW. A randomised controlled trial of clinical outreach education to rationalise antibiotic prescribing for acute dental pain in the primary care setting. Br Dent J. 2006 Aug 26;201(4):217-22; discussion 216. doi: 10.1038/sj.bdj.4813879. PMID: 16902573.
114.Sharma S, Gupta U, Roy Chaudhury R, Bapna JS. Prescribing Behaviour of Physicians. *Journal of Health Management*. 2002;4(1):55-71. doi:10.1177/097206340200400106
115.Shen X, Lu M, Feng R, Cheng J, Chai J, Xie M, Dong X, Jiang T, Wang D. Web-Based Just-in-Time Information and Feedback on Antibiotic Use for Village Doctors in Rural Anhui, China: Randomized Controlled Trial. J Med Internet Res. 2018 Feb 14;20(2):e53. doi: 10.2196/jmir.8922. PMID: 29444768; PMCID: PMC5830611.
116.Simon SR, Smith DH, Feldstein AC, Perrin N, Yang X, Zhou Y, Platt R, Soumerai SB. Computerized prescribing alerts and group academic detailing to reduce the use of potentially inappropriate medications in older people. J Am Geriatr Soc. 2006 Jun;54(6):963-8. doi: 10.1111/j.1532-5415.2006.00734.x. PMID: 16776793.
117.Simula AS, Jenkins HJ, Hancock MJ, Malmivaara A, Booth N, Karppinen J. Patient education booklet to support evidence-based low back pain care in primary care - a cluster randomized

controlled trial. BMC Fam Pract. 2021 Sep 7;22(1):178. doi: 10.1186/s12875-021-01529-2. PMID: 34493219; PMCID: PMC8422671.

118. Søndergaard J, Andersen M, Støvring H, Kragstrup J. Mailed prescriber feedback in addition to a clinical guideline has no impact: a randomised, controlled trial. Scand J Prim Health Care. 2003 Mar;21(1):47-51. doi: 10.1080/02813430310000564. PMID: 12718461.
119. Thomas RE, Croal BL, Ramsay C, Eccles M, Grimshaw J. Effect of enhanced feedback and brief educational reminder messages on laboratory test requesting in primary care: a cluster randomised trial. Lancet. 2006 Jun 17;367(9527):1990-6. doi: 10.1016/S0140-6736(06)68888-0. PMID: 16782489.
120. Tierney WM, Miller ME, McDonald CJ. The effect on test ordering of informing physicians of the charges for outpatient diagnostic tests. N Engl J Med. 1990 May 24;322(21):1499-504. doi: 10.1056/NEJM199005243222105. PMID: 2186274.
121. Torrente F, Bustin J, Triskier F, Ajzenman N, Tomio A, Mastai R, Lopez Boo F. Effect of a Social Norm Email Feedback Program on the Unnecessary Prescription of Nimodipine in Ambulatory Care of Older Adults: A Randomized Clinical Trial. JAMA Netw Open. 2020 Dec 1;3(12):e2027082. doi: 10.1001/jamanetworkopen.2020.27082. PMID: 33306114; PMCID: PMC7733153.
122. Trietsch J, van Steenkiste B, Grol R, Winkens B, Ulenkate H, Metsemakers J, van der Weijden T. Effect of audit and feedback with peer review on general practitioners' prescribing and test ordering performance: a cluster-randomized controlled trial. BMC Fam Pract. 2017 Apr 13;18(1):53. doi: 10.1186/s12875-017-0605-5. PMID: 28407754; PMCID: PMC5390393.
123. Urbiztondo I, Bjerrum L, Caballero L, Suarez MA, Olinisky M, Córdoba G. Decreasing Inappropriate Use of Antibiotics in Primary Care in Four Countries in South America-Cluster Randomized Controlled Trial. Antibiotics (Basel). 2017 Dec 14;6(4):38. doi: 10.3390/antibiotics6040038. PMID: 29240687; PMCID: PMC5745481.
124. van Bokhoven MA, Koch H, van der Weijden T, Weekers-Muyres AH, Bindels PJ, Grol RP, Dinant GJ. The effect of watchful waiting compared to immediate test ordering instructions on general practitioners' blood test ordering behaviour for patients with unexplained complaints; a randomized clinical trial (ISRCTN55755886). Implement Sci. 2012 Apr 4;7:29. doi: 10.1186/1748-5908-7-29. PMID: 22475083; PMCID: PMC3353203.
125. van der Velden AW, Kuyvenhoven MM, Verheij TJ. Improving antibiotic prescribing quality by an intervention embedded in the primary care practice accreditation: the ARTI4 randomized trial. J Antimicrob Chemother. 2016 Jan;71(1):257-63. doi: 10.1093/jac/dkv328. Epub 2015 Oct 21. PMID: 26490015.
126. van Driel ML, Coenen S, Dirven K, Lobbestael J, Janssens I, Van Royen P, Haaijer-Ruskamp FM, De Meyere M, De Maeseneer J, Christiaens T. What is the role of quality circles in strategies to optimise antibiotic prescribing? A pragmatic cluster-randomised controlled trial in primary care. Qual Saf Health Care. 2007 Jun;16(3):197-202. doi: 10.1136/qshc.2006.018663. PMID: 17545346; PMCID: PMC2464984.
127. van Eijk ME, Avorn J, Porsius AJ, de Boer A. Reducing prescribing of highly anticholinergic antidepressants for elderly people: randomised trial of group versus individual academic detailing. BMJ. 2001 Mar 17;322(7287):654-7. doi: 10.1136/bmj.322.7287.654. PMID: 11250852; PMCID: PMC26547.
128. Vugt SV, de Schepper E, van Delft S, Zuithoff N, de Wit N, Bindels P. Effectiveness of professional and patient-oriented strategies in reducing vitamin D and B12 test ordering in primary care: a cluster randomised intervention study. BJGP Open. 2021 Dec 14;5(6):BJGPO.2021.0113. doi: 10.3399/BJGPO.2021.0113. PMID: 34407963; PMCID: PMC9447297.

129. Verstappen WH, van der Weijden T, Dubois WI, Smeele I, Hermsen J, Tan FE, Grol RP. Improving test ordering in primary care: the added value of a small-group quality improvement strategy compared with classic feedback only. Ann Fam Med. 2004 Nov-Dec;2(6):569-75. doi: 10.1370/afm.244. PMID: 15576543; PMCID: PMC1466745.
130. Verstappen WH, van der Weijden T, Sijbrandij J, Smeele I, Hermsen J, Grimshaw J, Grol RP. Effect of a practice-based strategy on test ordering performance of primary care physicians: a randomized trial. JAMA. 2003 May 14;289(18):2407-12. doi: 10.1001/jama.289.18.2407. PMID: 12746365.
131. Vervloet M, Meulepas MA, Cals JW, Eimers M, van der Hoek LS, van Dijk L. Reducing antibiotic prescriptions for respiratory tract infections in family practice: results of a cluster randomized controlled trial evaluating a multifaceted peer-group-based intervention. NPJ Prim Care Respir Med. 2016 Feb 4;26:15083. doi: 10.1038/npjpcrm.2015.83. PMID: 26845640; PMCID: PMC4741286.
132. Vicentini M, Mancuso P, Giorgi Rossi P, Di Pede S, Pellati M, Gandolfi A, Zoboli D, Riccò D, Busani C, Ferretti A. A cluster randomized trial to measure the impact on nonsteroidal anti-inflammatory drug and proton pump inhibitor prescribing in Italy of distributing cost-free paracetamol to osteoarthritic patients. BMC Fam Pract. 2019 Dec 6;20(1):169. doi: 10.1186/s12875-019-1050-4. PMID: 31810456; PMCID: PMC6896368.
133. Weller D, May F, Rowett D, Esterman A, Pinnock C, Nicholson S, Doust J, Silagy C. Promoting better use of the PSA test in general practice: randomized controlled trial of educational strategies based on outreach visits and mailout. Fam Pract. 2003 Dec;20(6):655-61. doi: 10.1093/fampra/cmg606. PMID: 14701888.
134. Welschen I, Kuyvenhoven MM, Hoes AW, Verheij TJ. Effectiveness of a multiple intervention to reduce antibiotic prescribing for respiratory tract symptoms in primary care: randomised controlled trial. BMJ. 2004 Aug 21;329(7463):431. doi: 10.1136/bmj.38182.591238.EB. Epub 2004 Aug 5. PMID: 15297305; PMCID: PMC514206.
135. Winkens RA, Pop P, Bugter-Maessen AM, Grol RP, Kester AD, Beusmans GH, Knottnerus JA. Randomised controlled trial of routine individual feedback to improve rationality and reduce numbers of test requests. Lancet. 1995 Feb 25;345(8948):498-502. doi: 10.1016/s0140-6736(95)90588-x. PMID: 7861879.
136. Worrall G, Hutchinson J, Sherman G, Griffiths J. Diagnosing streptococcal sore throat in adults: randomized controlled trial of in-office aids. Can Fam Physician. 2007 Apr;53(4):666-71. Erratum in: Can Fam Physician. 2007 Jun;53(6):1006. PMID: 17872717; PMCID: PMC1952596.
137. Wächtler H, Kaduszkiewicz H, Kuhnert O, Malottki KA, Maaß S, Hedderich J, Wiese B, Donner-Banzhoff N, Hansmann-Wiest J. Influence of a guideline or an additional rapid strep test on antibiotic prescriptions for sore throat: the cluster randomized controlled trial of HALS (Hals und Antibiotika Leitlinien Strategien). BMC Prim Care. 2023 Mar 20;24(1):75. doi: 10.1186/s12875-023-01987-w. PMID: 36941540; PMCID: PMC10029262.
138. Yang J, Cui Z, Liao X, He X, Wang L, Wei D, Wu S, Chang Y. Effects of a feedback intervention on antibiotic prescription control in primary care institutions based on a Health Information System: a cluster randomized cross-over controlled trial. J Glob Antimicrob Resist. 2023 Jun;33:51-60. doi: 10.1016/j.jgar.2023.02.006. Epub 2023 Feb 23. PMID: 36828121.
139. Yip W, Powell-Jackson T, Chen W, Hu M, Fe E, Hu M, Jian W, Lu M, Han W, Hsiao WC. Capitation combined with pay-for-performance improves antibiotic prescribing practices in rural China. Health Aff (Millwood). 2014 Mar;33(3):502-10. doi: 10.1377/hlthaff.2013.0702. Epub 2014 Feb 26. PMID: 24572187.

140. Zwar N, Wolk J, Gordon J, Sanson-Fisher R, Kehoe L. Influencing antibiotic prescribing in general practice: a trial of prescriber feedback and management guidelines. Fam Pract. 1999 Oct;16(5):495-500. doi: 10.1093/fampra/16.5.495. PMID: 10533946.
