# Supplementary material for: Effectiveness of different de-implementation strategies in primary care: systematic review and meta-analysis
Source: BMJ Med. 2025 Sep 9;4(1):e001343. doi: 10.1136/bmjmed-2025-001343 (PMC12421606; doi:10.1136/bmjmed-2025-001343)
Supplement: Supplementary file 2 [file bmjmed-2025-001343.reviewer_comments.pdf]

**PEER REVIEW HISTORY**

BMJ Medicine publishes all reviews undertaken for accepted manuscripts. Reviewers are asked to complete a checklist review form and are provided with free text boxes to elaborate on their assessment. These free text comments are reproduced below.

**ARTICLE DETAILS**

| | |
|---|---|
| **TITLE (PROVISIONAL)** | Effectiveness of different de-implementation strategies in primary care: a systematic review and meta-analysis |
| **AUTHORS** | Raudasoja, Aleksi; Parpia, Sameer; Mustonen, Jussi; Vernooij, Robin; Falkenbach, Petra; Aoki, Yoshitaka; Barchuk, Anton; Blanker, Marco; Cartwright, Rufus; Crowder, Kathryn; Garcia-Perdomo, Herney; Gutschon, Rachel; Halme, Alex; Kilpeläinen, Tuomas; Kuitunen, Ilari; Lamberg, Tiina; Lang, Eddy; Matos, Jenifer; Nevalainen, Olli; Nordlund, Niko; Pourjamal, Negar; Raittio, Eero; richard, patrick; Violette, Philippe; Komulainen, Jorma; Sipilä, Raija; Tikkinen, Kari |

**VERSION 1 - REVIEW**

| | |
|---|---|
| **REVIEWER NAME** | Kar, Debasish |
| **REVIEWER AFFILIATION** | University of Plymouth Faculty of Health |
| **REVIEWER CONFLICT OF INTEREST** | None |
| **AI DISCLOSURE** | No |
| **DATE REVIEW RETURNED** | 19-Jan-2025 |

| | |
|---|---|
| **GENERAL COMMENTS** | Line 12 - Did the authors search for unpublished and grey literature?<br>Line 29 - Please add a sentence to describe PICO<br>Line 47 - As you have excluded primarily paediatric studies, consider adding it in the title that this systematic review is focused on adults. The majority of low-value care and inappropriate antibiotic prescribing is for children due to parental anxiety/expectations/health beliefs.<br>Line 56 - ? Intensity of low-value care<br><br>Results - line 31-32 - please explain what binary and continuous outcomes were measured for low-value care |

| | |
|---|---|
| **REVIEWER NAME** | Tri Yuniar, Cindra |
| **REVIEWER AFFILIATION** | Department of Pharmacology and Clinical Pharmacy, School of Pharmacy, Institut Teknologi Bandung, Bandung 40132, Indonesia |
| **REVIEWER CONFLICT OF INTEREST** | None |
| **AI DISCLOSURE** | No |
| **DATE REVIEW RETURNED** | 05-Feb-2025 |

| | |
|---|---|
| **GENERAL COMMENTS** | 1. Please mention the author(s) who did the step in every step (searching, identifying, and hand-researching of reports or study protocol). The review should be checked by minimum 3 authors for every step.<br>2. Can the authors explain the specific number of publications in |

| | |
|---|---|
| | every step? So, we will easily find the sort strategies, how many study are excluded, and how many study are added for meta-analysis, etc. Authors mentioned Figure 1 as the results of searching strategies, but the Figure 1 is about Risk of Bias, wrong picture?<br>3. This study was good and concise for intervention strategies in primary care |

| | |
|---|---|
| **REVIEWER NAME** | Riley, Richard |
| **REVIEWER AFFILIATION** | University of Birmingham, Institute of Applied Health Research |
| **REVIEWER CONFLICT OF INTEREST** | None |
| **AI DISCLOSURE** | No |
| **DATE REVIEW RETURNED** | 23-Feb-2025 |

| | |
|---|---|
| **GENERAL COMMENTS** | Thank you for the opportunity to review this systematic review. It is clear a huge amount of work has been undertaken to do this review and meta-analysis, and to write the study for publication. It is great to see such wealth of information in the supp material, and the paper is well-written.<br>I have reviewed this from a statistical perspective, and have a number of comments for the authors to consider, to help improve clarity of their meta-analyses and summary results, for the reader, and to address potential limitations.<br><br>1) Odds ratios are synthesised, but these relate to a specific time point (e.g. odds of low-value care use by 1 month, 6 months, 12 months?) – so I cannot understand how to interpret the results without a time point mentioned (e.g. in the abstract)<br>2) Following this, it appears the trials are synthesising results at widely different follow-up times (See tables in supp material), so what does the pooled OR actual relate to? Ratio of odds of low-value care use by when exactly? Further, continuous outcomes are converted to odds ratios, but again how is this compatible with the ORs from other studies using binary outcome definitions.<br>3) A related point: abstract and what this study ads needs to define what we mean by 'low-value care', and how differences in this definition were handled across studies?<br>4) The intervention of interest is broad (de-implementation strategies), and we have a number of different sub-types of this intervention of interest. These are examined in different numbers of studies, and so it is difficult to make decisions or inferences about what the 'best' de-implementation strategy is, based on pairwise meta-analyses alone. Therefore, I query why a network meta-analysis was not conducted to provide a more coherent synthesis of the evidence, especially when the paper makes statements about what has the 'greatest impact' and whether multiple strategies do better than single strategies. Therefore, I recommend a network meta-analysis is added or, at the bare minimum, the conclusions (about which is best etc) are tempered and limitations about this made clear throughout, especially the discussion, where further research for a network MA can be signposted.<br>5) The abstract and results section need to discuss more whether there is heterogeneity in the effects across studies. The authors report I-squared, but this does not actually measure heterogeneity directly. Rather, we need the estimate of tau (between study SD). Further, given that there most likely is heterogeneity, focusing only on the summary result is questionable and a wonder whether a |

| | |
|---|---|
| | prediction interval may be a complementary summary to provide.<br>6) In the methods section, it says “Methodologically trained reviewers applied these forms to screen study reports for eligibility, data extraction and risk of bias assessment independently and in duplicate. Disagreements were resolved through discussion” – please clarity on exactly how many reviewers were involved, and how many were involved in the resolving disagreements.<br>7) Given that most studies are cluster RCTs, we need some assurance that the extracted effect estimates actually accounted for the clustering of patients within e.g. practices. I can’t see that mentioned.<br>8) Some of the interpretations need to be clearer in the discussion, regarding the magnitude of effects. e.g. ‘We found a 16% relative effect of patient education alone and a 30-40% relative decrease in low-value care with patient education combined with provider education and other strategies.’ Relative decrease in what? Use? Risk of use? Odds of use? By what time point? Etc<br>9) Table 1 provides relative effects and absolute risk reduction, but this seems overly strong given the heterogeneity in both baseline risks across studies and patients, and treatment effects across studies. I worry that the ‘absolute effect’ provided relates to some average effect and median patient, and thus won’t apply to most studies or individuals in practice, and hides the heterogeneity. Also, CIs are strangely missing from these. I would remove these results entirely.<br><br>In summary, in the context of clear hard work and a vast review, I hope these comments help the authors to improve their article moving forwards. |

| | |
|---|---|
| **REVIEWER NAME** | Levinson, Wendy |
| **REVIEWER AFFILIATION** | St Michael's Hospital |
| **REVIEWER CONFLICT OF INTEREST** | None |
| **AI DISCLOSURE** | No |
| **DATE REVIEW RETURNED** | 06-Mar-2025 |

| | |
|---|---|
| **GENERAL COMMENTS** | This is an interesting paper which addresses a common issue—how do we best influence practice in primary care. While I am aware of the literature, this does pull it together in a useful and cogent fashion. The findings related to the strength of patient education as an intervention was new to me—I was not aware of the relative strength of this intervention compared to other approaches. On the other hand, the findings that multiple strategies combined are best was not new information—but confirmation.<br><br>I found the tables and appendices hard to read. Overall the paper was methodologically challenging and somewhat overwhelming. Could some of the key tables or plots be incorporated more simply?<br><br>Overall, the paper is highly relevant to the efforts to reduce low value care and the primary care setting is particularly important.<br><br>Thank you for this work. |

## VERSION 1 – AUTHOR RESPONSE

Reviewer: 1
Dr. Debasish Kar, University of Plymouth Faculty of Health
Comments to the Author

**Comment #1:** *"Line 12 - Did the authors search for unpublished and grey literature?"*

RESPONSE

1) First, we searched MEDLINE and Scopus. Second, we identified systematic reviews, searched their reference lists, and added potential articles to the selection process. Third, we hand-searched for study protocols and other additional study reports. Although we did not specifically search for conference abstracts, our search identified also conference abstracts. We did not, however, include conference abstracts because they are unlikely to provide enough information required and may also risk for publication bias.
2) Prompted by the reviewer comment, we have revised the manuscript as follows: *"If we did not find full text manuscript for a conference abstract, we excluded them."* to the methods text.
3) Page 7, Line 186

**Comment #2:** *"Line 29 - Please add a sentence to describe PICO"*

RESPONSE

1) Thank you for the comment. We revised the PICO description at the end of the introduction section to include also the comparator which was previously missing.
2) The revised descriptions is as follows: *"We conducted a comprehensive systematic review and meta-analysis of the effectiveness of different de-implementation strategies compared to no intervention or other de-implementation strategies in reducing low-value care use in primary care."*
3) Page 6, Lines 155-157

**Comment #3:** *"Line 47 - As you have excluded primarily paediatric studies, consider adding it in the title that this systematic review is focused on adults. The majority of low-value care and inappropriate antibiotic prescribing is for children due to parental anxiety/expectations/health beliefs."*

RESPONSE

1) Thank you for pointing out the importance of pediatric low-value practices. As we considered the de-implementation process potentially different for paediatric than adult patients, we excluded trials that primarily focused on paediatric practices. We included trials where all patients were adult or where the majority of the patient population was adult, even if a minority of participants were children. Therefore, we believe that specifying 'adult patients' in the title would not necessarily be the most optimal choice.
2) Prompted by the reviewer suggestion, we have clarified this issue in the revised methods as follows: *"We excluded trials that primarily studied paediatric practices (i.e., trials were excluded if they reported that majority of participants were children; trials were included if children were involved but comprised less than 50% of the study population)."*
3) Page 7, Line 184-187

**Comment #4:** *“Line 56 - ? Intensity of low-value care”*

RESPONSE

1) We checked all instances of 'Line 56' in each page but were unable to find any reference to 'Intensity of low-value care.' Could the reviewer or editors kindly clarify the nature of this comment?

**Comment #5:** *“line 31-32 - please explain what binary and continuous outcomes were measured for low-value care”*

RESPONSE

1) Thank you for the comment. Prompted by this comment, we have revised and clarified the text.
2) The revised text is the following: *“Of all trials, 62 (44%) trials used binary outcomes and 78 (56%) continuous outcomes to measure low-value care use. Of the trials that used binary outcomes, 42 aimed to reduce the use of antibiotics, 8 other drug treatments, 9 imaging, and 5 laboratory testing; and of the trials that used continuous outcomes, 33 aimed to reduce the use of antibiotics, 34 other drug treatments, 8 imaging, and 10 laboratory testing.”*
3) Page 11, Lines 289-294

Reviewer: 2
Dr. Cindra Tri Yuniar, Department of Pharmacology and Clinical Pharmacy, School of Pharmacy, Institut Teknologi Bandung, Bandung 40132, Indonesia
Comments to the Author

**Comment #1:** *"Please mention the author(s) who did the step in every step (searching, identifying, and hand-researching of reports or study protocol). The review should be checked by minimum 3 authors for every step."*

1) Thank you for the comment. Cochrane Handbook states as follows: "Use (at least) two people working independently to determine whether each study meets the eligibility criteria. Ideally, screening of titles and abstracts to remove irrelevant reports should also be done in duplicate by two people working independently (although it is acceptable that this initial screening of titles and abstracts is undertaken by only one person)." This is the guidance we have followed. Prompted by the reviewer comment, in the revised manuscript, besides citing the Cochrane Handbook, we revised the text.
We also followed the BMJ Medicine author guidance, which states "*Contributorship – we publish a contributorship statement at the end of the paper, giving details of who did what in planning, conducting, and reporting the work. This should include all author contributors and may include non-author contributors."* In line with this, we have detailed the authors involved in each step of the process in the **Contributions** section at the end of the article. If the Editors prefer that this information also be included in the main text, we are open to doing so. However, in order to maintain conciseness, we have not yet added it to the main text.
2) Revised text is as follows: ""*We developed standardized forms with detailed guidance for screening abstracts and full texts, data extraction and assessment of risk of bias. For each study report, two methodologically trained reviewers applied these forms to screen for eligibility, data extraction and risk of bias assessment independently and in duplicate (). Disagreements were resolved through discussion between the two reviewers and, if necessary, with a clinician-methodologist adjudicator.*""
3) Page 8, Line 207-211

**Comment #2:** *"Can the authors explain the specific number of publications in every step? So, we will easily find the sort strategies, how many study are excluded, and how many study are added for meta-analysis, etc. Authors mentioned Figure 1 as the results of searching strategies, but the Figure 1 is about Risk of Bias, wrong picture?"*

RESPONSE

1) Thank you for the comment. Figure 1 is the flow chart of the study that is part of the main manuscript. Supplementary Figure 1 is the risk of bias figure that is part of the Supplementary information / Appendix. Prompted by the reviewer, we have checked citations to the each figure and found that once we cited Supplementary Figure 1 wrongly as Supplementary Figure 2.
2) We have revised the citation of Supplemantary Figure 1 as correct.
3) Page 11, line 307

**Comment #3:** *"This study was good and concise for intervention strategies in primary care"*

RESPONSE

1) Thank you for your comments and advice how to improve our paper. No changes required based on this comment.

Reviewer: 3
Prof. Richard Riley, University of Birmingham
Comments to the Author
Thank you for the opportunity to review this systematic review. It is clear a huge amount of work has been undertaken to do this review and meta-analysis, and to write the study for publication. It is great to see such wealth of information in the supp material, and the paper is well-written.
I have reviewed this from a statistical perspective, and have a number of comments for the authors to consider, to help improve clarity of their meta-analyses and summary results, for the reader, and to address potential limitations.

**Comment #1:** *"Odds ratios are synthesised, but these relate to a specific time point (e.g. odds of low-value care use by 1 month, 6 months, 12 months?) – so I cannot understand how to interpret the results without a time point mentioned (e.g. in the abstract). Following this, it appears the trials are synthesising results at widely different follow-up times (See tables in supp material), so what does the pooled OR actual relate to? Ratio of odds of low-value care use by when exactly?"*

RESPONSE

1) Thank you for the comment. We considered this issue carefully both before and after conducting the analyses. Of the 140 trials, only seven (5%) reported both short - and long-term follow-up data. In four trials, the point estimates were similar, in two smaller (indicating a higher impact) for short-term follow-up, and in one smaller for long-term follow-up. Based on this limited evidence, the impact of follow-up duration is uncertain but probably not substantial.
2) As suggested, the revised results provide information on median (with interquartile range) follow-up time for each intervention category. Additionally, the revised abstract provides median (with interquartile range) follow-up time for all trials. Finally, we have also added discussion on this issue as a potential limitation of our study as follows: "Third, we were unable to generate precise estimates for specific time points. However, this may not represent a major limitation. Seven trials provided both short- and long-term follow-up data. In four of these trials, the point estimates were similar, in two smaller (indicating a higher impact) for short-term follow-up, and in one smaller for long-term follow-up. Based on this limited evidence, the impact of follow-up duration is uncertain but probably not substantial."
3) Page 4, Line 113; page 12-14; and page 18, lines 534-536

**Comment #2:** *"Further, continuous outcomes are converted to odds ratios, but again how is this compatible with the ORs from other studies using binary outcome definitions."*

RESPONSE

1) Thank you for the very important comment on converted continuous outcomes.

   When planning the analysis, we discussed these issues extensively. We identified two approaches for the analysis which both have some but different limitations:

1. What are the limitations of providing two effect estimates in each intervention category, one for continuous and one for binary outcomes?
    a. Only two of the eight intervention categories (provider education; provider education + feedback) included at least three trials reporting both binary and continuous outcomes, allowing for pooling. For the remaining six categories, we could not provide both continuous and binary effect estimates, making comparisons between categories difficult due to differences in outcome types.
    b. For two of the eight intervention categories, effect estimates would be available for both continuous and binary outcomes. However, interpreting these results would be challenging. Differences in estimates could reflect true variation in intervention effects between studies using different outcome types, differences in measurement and interpretation, or random error due to limited statistical power.

2. One effect size (OR, odds ratio) for all intervention categories
    a. When a point estimate is converted to an odds ratio (OR), the result depends on the standard deviation. Although this conversion is suggested as an option in Cochrane guidance, it can make the effect estimate harder to interpret. This issue is similar to the challenges encountered when using standardized mean differences for pooling.
    b. Dependency on variance affects the effect estimates, potentially leading to trials with continuous outcomes receiving less weight in the meta-analysis.

Considering these issues, we decided to primarily provide one pooled effect estimate (odds ratio) for each outcome, conduct sensitivity analyses on continuous and binary outcomes (for each outcome when possible), and discuss the limitations as well as interpretation of sensitivity analysis in the “strengths and limitations” section.

Three out of four sensitivity analyses using continuous outcomes suggested no credible differences with the pooled analyses. These analyses included three intervention categories that had at least three trials with continuous outcomes. One out of four sensitivity analyses using continuous outcomes suggested smaller impact (continuous outcomes on provider education combined with audit and feedback) with the pooled analyses. All five sensitivity analyses using binary outcomes suggested very similar effects with the pooled analyses. Overall, results of these nine sensitivity analyses are reassuring for our analysis methods.

In addition to testing the credibility of the estimates in our primary analysis, the sensitivity analyses will also help more experienced readers to understand the effects of different de-implementation interventions.

2) We revised the text in the supplementary methods as well as in the limitations section of the main text to better describe our approach and justifications for it. The revised texts are as follows: *“We decided to provide one pooled effect estimate (odds ratio) for each outcome. An alternative approach would have been to provide separate pooled effect estimates for continuous and binary outcomes (we did these as sensitivity analyses). Providing separate effect sizes for each outcome would likely decrease the interpretability and comparability of findings between different intervention types.”*

The revised text on limitations is as follows: “Second, since the included RCTs used both continuous and binary outcomes, we converted continuous outcomes to odds ratios. This method may have resulted in larger effect sizes and smaller weight in meta-analyses as the effect sizes are dependent on both standard deviation and

magnitude of effect. We therefore conducted sensitivity analyses separately for both binary and continuous outcomes, which showed no large differences."

3) Page 18, Lines 526-530, and supplementary Methods 5

**Comment #3:** *"A related point: abstract and what this study ads needs to define what we mean by 'low-value care', and how differences in this definition were handled across studies?"*

RESPONSE

1) Thank you. We agree with the comment and revised these sections accordingly.
2) Revisions:
   ***a.*** We added short definition to the abstract: *"Objective: To evaluate the effectiveness of various de-implementation interventions in primary care targeting low-value care, that is care (treatments or tests) providing no or limited value for patients. in primary care. (low-value care)."* Furthermore, we clarified the term in the key messages box: *"Low-value care provides little to no value for patients, may be harmful for patients, and by wasting limited resources, is a major problem in modern healthcare, including primary care."*
   b. We revised supplementary Methods 5 as follows: "*In a scoping review conducted before this study, we categorized the outcomes (used in de-implementation trials) to low-value care and total volume care outcomes. We judged total volume care (potentially including both low-value care and appropriate care) as indirect evidence and considered rating down the evidence certainty if half or more trials provided only total volume outcomes.* "
3) Pages 4, lines 100-102, page 5, and supplementary Methods 5.

**Comment #4:** "*The intervention of interest is broad (de-implementation strategies), and we have a number of different sub-types of this intervention of interest. These are examined in different numbers of studies, and so it is difficult to make decisions or inferences about what the 'best' de-implementation strategy is, based on pairwise meta-analyses alone. Therefore, I query why a network meta-analysis was not conducted to provide a more coherent synthesis of the evidence, especially when the paper makes statements about what has the 'greatest impact' and whether multiple strategies do better than single strategies. Therefore, I recommend a network meta-analysis is added or, at the bare minimum, the conclusions (about which is best etc) are tempered and limitations about this made clear throughout, especially the discussion, where further research for a network MA can be signposted."*

RESPONSE

1) We agree with the comment and revised the abstract and main text accordingly.
2) We toned down the abstract and discussion as well as recommended network meta-analysis to be conducted in the future.
   Our revised abstract states as follows: "*Single strategies may slightly reduce low-value care use but achieving a meaningful impact on low-value care may require exploiting multiple strategies. Of these multiple strategies, those incorporating patient education may have the greatest impact on low-value care use."*
   The revised first paragraph of the Discussion includes the following sentence: *"Interventions with multiple strategies, particularly those incorporating patient education, may lead to greater impact than single strategies"*

We added the following sentence to the Limitations: "*Fifth, we had a limited number of direct comparisons between different de-implementation strategies. Future research could explore this further, for example by conducting a network meta-analysis to compare the effectiveness of various strategies.*"

3) Page 4, Lines 126-127 and page 16, line 444, and page 18, line 540-542

**Comment #5:** "*The abstract and results section need to discuss more whether there is heterogeneity in the effects across studies. The authors report I-squared, but this does not actually measure heterogeneity directly. Rather, we need the estimate of tau (between study SD). Further, given that there most likely is heterogeneity, focusing only on the summary result is questionable and a wonder whether a prediction interval may be a complementary summary to provide.*"

1) Thank you for the comment. We agree and revised the text and supplement accordingly.
2) We added tau to each meta-analysis in the results section as well as to forest plots in the Supplementary file for each intervention category.

**Comment #6:** "*In the methods section, it says "Methodologically trained reviewers applied these forms to screen study reports for eligibility, data extraction and risk of bias assessment independently and in duplicate. Disagreements were resolved through discussion" – please clarity on exactly how many reviewers were involved, and how many were involved in the resolving disagreements.*"

1) We agree with the comment and add these clarifications to the methods section.
2) The revised text in the methods is as follows: *"For each study report, two methodologically trained reviewers applied these forms to screen study reports for eligibility, data extraction and risk of bias assessment independently. Disagreements were resolved through discussion between the two reviewers and, if necessary, with a clinician-methodologist adjudicator."*
3) Page 8, lines 208-211

**Comment #7:** "*Given that most studies are cluster RCTs, we need some assurance that the extracted effect estimates actually accounted for the clustering of patients within e.g. practices. I can't see that mentioned.*"

1) We agree with the comment and revised the main text (methods section) accordingly. Furthermore, the details on the issue are in Supplementary Methods 5: "Primarily, we used reported ICC or if that was not available, median ICC from all trials: for binary variable, practice-level ICC 0.105 and provider-level ICC 0.22; and for continuous variables ICC 0.305."
2) The revised text in the methods is as follows: *"For cluster RCTs, if effect estimates did not account for clustering or only arm-specific estimates were available, we adjusted for clustering (Supplementary Methods 5). "*
3) Page 9, Lines 247-249

**Comment #8:** "*Some of the interpretations need to be clearer in the discussion, regarding the magnitude of effects. e.g. 'We found a 16% relative effect of patient education alone and a 30-40% relative decrease in low-value care with patient education combined with provider education and other strategies.' Relative decrease in what? Use? Risk of use? Odds of use? By what time point? Etc"*

1) Thank you for the comments. We agree that these issues need clarification.
2) We have revised the following sentences as follows:
   First paragraph of the discussion: "*Overall, de-implementation strategies led to relative risk reductions of 10% to 35% in low-value care use (median follow up 287 days).*"
   *Implications: "The estimated 23% relative risk reduction in low-value care use from these interventions may be further enhanced by increasing the intervention intensity."* And *"We found low certainty evidence that provider education leads to a 10% relative risk decrease in low-value care use (Table 1)." and "We found a 16% relative risk reduction for patient education alone and a 30-34% relative risk decrease in low-value care use with patient education combined with provider education and other strategies"*
3) Page 16-17, Lines 443-489

**Comment #9:** "*Table 1 provides relative effects and absolute risk reduction, but this seems overly strong given the heterogeneity in both baseline risks across studies and patients, and treatment effects across studies. I worry that the 'absolute effect' provided relates to some average effect and median patient, and thus won't apply to most studies or individuals in practice, and hides the heterogeneity. Also, CIs are strangely missing from these. I would remove these results entirely."*

1) Although we agree with the reviewer that these estimates have uncertainties, we think understanding the absolute effect of intervention would be of utmost importance to clinicians or other decision makers. We measured the relative effect by using the median risk of low-value care used in control groups of the included trials in each intervention category. Due to better readability, this method, despite its limitations, is suggested by Cochrane handbook (section 15.4.4.4).
2) Revisions:
   a. Prompted by the reviewer comments, we removed the absolute effect from the Table 1. The removed information is available in the revised supplementary file table 16.
   b. Regarding the relative effect estimates, we added confidence intervals to them and specified the methods used for these estimations in the footnotes.
   c. For consistency, we also removed the absolute effects from the main text.
3) Table 1, supplementary Table 16

In summary, in the context of clear hard work and a vast review, I hope these comments help the authors to improve their article moving forwards.

Prof Richard Riley
BMJ Statistics Editor.

RESPONSE: Thank you very much for the great comments. Hope our response improved the paper. We are happy to discuss further revisions when needed.

Reviewer: 4
Prof. Wendy Levinson, St Michael's Hospital, University of Toronto - St George Campus

**Comment #1:** *"This is an interesting paper which addresses a common issue—how do we best influence practice in primary care. While I am aware of the literature, this does pull it together in a useful and cogent fashion. The findings related to the strength of patient education as an intervention was new to me—I was not aware of the relative strength of this intervention compared to other approaches. On the other hand, the findings that multiple strategies combined are best was not new information—but confirmation.*

*I found the tables and appendices hard to read. Overall the paper was methodologically challenging and somewhat overwhelming. Could some of the key tables or plots be incorporated more simply?"*

RESPONSE

1) Thank you for this important comment. Prompted by this and other comments, we have tried to simplify the text and tables of the manuscript. We agree that we have a large number of appendices. They are, however, not meant to be used by all readers rather for those conducting research on this area or otherwise interested in the details of our methods and results.
2) We have revised Table 1 by erasing the absolute effect estimates. We believe the relative estimates are the easiest to apply by decision makers, so we left them to Table 1. Furthermore, we added the relative effect for provider education combined with audit and feedback to the key messages box. We hope these revisions have made the paper easier to read and are open to further revisions if Editors and reviewers see needed.
3) Table 1, and page 4

**Comment #2:** "*Overall, the paper is highly relevant to the efforts to reduce low value care and the primary care setting is particularly important.*

*Thank you for this work."*

RESPONSE

1) Thank you for the valuable feedback and positive comments.

**VERSION 2 – REVIEW**

| | |
|---|---|
| **REVIEWER NAME** | Riley, Richard |

| | |
|---|---|
| **REVIEWER AFFILIATION** | University of Birmingham, Institute of Applied Health Research |
| **REVIEWER CONFLICT OF INTEREST** | None |
| **AI DISCLOSURE** | No |
| **DATE REVIEW RETURNED** | 16-May-2025 |

| | |
|---|---|
| **GENERAL COMMENTS** | I would like to thank the reviewer for a very clear and thorough response to my previous comments. The article revision is improved. I have a few important comments remaining, which I am sure the authors can address.<br><br>1) I mentioned about doing the network meta-analysis – the authors haven't don't his but have included this as a note for further research, which is fine, but this means they must restrain from making any comparisons across the interventions in terms of which is 'best'. Currently, they still compare them, but as this is not done using a NMA, it is inappropriate. For example in the abstract conclusion they say 'Of these multiple strategies, those incorporating patient education may have the greatest impact on low -value care use.' – also see the first paragraph in the main discussion of the paper. The authors need to carefully remove all such statements, and I would also suggest recommending the NMA in the abstract conclusions.<br>2) 'We converted effect estimates from continuous variables to ORs according to Cochrane Handbook guidance' – please state explicitly what you did for clarity (the Handbook is a big book!).<br>3) Methods section needs to define how heterogeneity was quantified and examined (the authors did not respond about my suggestion to also include 95% prediction intervals, given the anticipated heterogeneity this makes more sense to me than focusing on summary estimates and CIs: https://pubmed.ncbi.nlm.nih.gov/21310794/)<br>4) The authors respond well to my concerns about different time points, but this is still not addressed in the methods section that I can tell. I think there needs to be discussion about this in the 'outcomes and variables' section, about what time points the authors were actually interested in, as this is a key aspect of their estimand.<br>5) Note that the estimate of tau (or tau^2) is not reporting in the main text for all the results. I think it should accompany any meta-analysis result given. Again, think about a prediction interval too. Tau is not provided in Table 1 of results either. Neither Figure 2 or 3.<br>6) 'However, tailored interventions of provider education combined with patient education (vs interventions without tailoring) led to larger reductions in low-value care use …' – this is a meta-regression result, so is prone to study-level confounding. Hence rather than saying 'led' (which is causal language) it is preferable to say is 'associated with'. Be cautious about these meta-regression results for this reason.<br><br>I look forward to receiving a further revision, and thank the authors in advance. |

## VERSION 2 – AUTHOR RESPONSE

Reviewer: 3
Prof. Richard Riley, University of Birmingham
Comments to the Author

**Comment #1:** *"I mentioned about doing the network meta-analysis – the authors haven't don't his but have included this as a note for further research, which is fine, but this means they must restrain from making any comparisons across the interventions in terms of which is 'best'. Currently, they still compare them, but as this is not done using a NMA, it is inappropriate. For example in the abstract conclusion they say 'Of these multiple strategies, those incorporating patient education may have the greatest impact on low-value care use.' – also see the first paragraph in the main discussion of the paper. The authors need to carefully remove all such statements, and I would also suggest recommending the NMA in the abstract conclusions."*

RESPONSE

4) Thank you for the comment. We have revised the text accordingly. As suggested, the revised manuscript no longer contains any statements claiming superiority of any intervention.
*5)* As recommended by the reviewer, we previously revised the sentence *"Single strategies may slightly reduce low-value care use but achieving a meaningful impact on low-value care may require exploiting multiple strategies."* and added a suggestion about the network meta-analysis in the limitations section: "*Fifth, we had a limited number of direct comparisons between different de-implementation strategies. Future research could explore this further, for example by conducting a network meta-analysis to compare the effectiveness of various strategies."*
We have now further revised the sentence in the Discussion from "Interventions with multiple strategies, particularly those incorporating patient education, may lead to greater impact than single strategies.*"* to *"Multi-strategy interventions including patient education, led to relative risk reductions of 30% to 35% in the use of low-value care."*. Additionally, we removed the sentence from the Abstract: *"Of these multiple strategies, those incorporating patient education may have the greatest impact on low-value care use*."
We also added the following sentence to the *Key Messages* box: *"A network meta-analysis could help future research explore which strategies have the greatest impact."* We are happy to move this to the Abstract if the Editors prefer, although we believe it fits more naturally in the *Key Messages* box.
Overall, we made these changes *to avoid any implication of direct comparison across interventions regarding which strategy is 'best'.*
6) page 4, lines 125-126, page 5, and page 16, lines 446-448,

**Comment #2:** *"'We converted effect estimates from continuous variables to ORs according to Cochrane Handbook guidance' – please state explicitly what you did for clarity (the Handbook is a big book!)."*

RESPONSE

4) Thank you for the comment! We have revised the text as suggested.
*5)* First, we added a further explanation in the main text: *"We converted effect estimates from continuous variables to standardized mean differences, and subsequently to ORs, following the guidance of the Cochrane Handbook (Supplementary Methods 5)."*
Second, we added the used formulas to the supplement (Supplementary Methods 5):
*"Below are all formulas used in the conversions:*

*1) $SMD = MD / SD^{pooled}$*
*2) $SE = (CI^{upper} – CI^{lower})/2 \times t$*
*3) $SD = SE / \sqrt{(1/N_1 + 1/N_2)}$*
*4) $\ln OR = SMD \times \pi/\sqrt{3}$”*

6) Page 9, lines 240-242, Supplementary Methods 5

**Comment #3:** *“Methods section needs to define how heterogeneity was quantified and examined (the authors did not respond about my suggestion to also include 95% prediction intervals, given the anticipated heterogeneity this makes more sense to me than focusing on summary estimates and CIs: https://pubmed.ncbi.nlm.nih.gov/21310794/)”*

RESPONSE

4) Thank you for the comment! We have revised the text as suggested. We added prediction intervals to the main analyses, updated the forest plots in the supplemental file, and cited the recommended reference in the Methods. In the revised Methods section, we described how we quantified heterogeneity.
5) Methods section now states: *“We calculated $I^2$ statistic and tau$^2$ to analyse the heterogeneity in the results. Furthermore, we calculated 95 % prediction intervals to estimate the expected range of effects in different settings.[18]”*
6) Page 9, lines 250-251

**Comment #4:** *“The authors respond well to my concerns about different time points, but this is still not addressed in the methods section that I can tell. I think there needs to be discussion about this in the ‘outcomes and variables’ section, about what time points the authors were actually interested in, as this is a key aspect of their estimand.”*

RESPONSE

2) Thank you for the comment! We have revised as suggested.
3) The revised Methods section states: *“If outcomes were measured at multiple time points, we extracted data for the last measurement before one year (short-term follow-up) and from the last at or beyond one year (long-term follow-up).”*
It alsostates: *“To provide a single summary estimate, we used ORs to represent the treatment effects for both continuous and binary outcomes at the final time point measured.”*
4) Page 9, lines 239-240

**Comment #5:** *“Note that the estimate of tau (or tau^2) is not reporting in the main text for all the results. I think it should accompany any meta-analysis result given. Again, think about a prediction interval too. Tau is not provided in Table 1 of results either. Neither Figure 2 or 3.”*

RESPONSE

4) Thank you for the comment! We previously provided tau^2 only for the main analysis. We have now added them to all analyses in the main text and to the forest plots in the supplementary file. Furthermore, we added tau to Table 1.
Since Figure 2 already presents the certainty of evidence, which also accounts for heterogeneity, we believe that adding $\tau^2$ may not be unnecessary. However, we have uploaded two versions of the figures to the submission system: with a $\tau^2$ column and

without. If the Editors consider it necessary, we are happy to use the version that includes $\tau^2$ in the publication.

5) The results section now includes $tau^2$ for all analyses. Furthermore, we have provided updated forest plots including $tau^2$ in the revised supplemental file.
6) Pages 12-15, Supplementary Figures 2-30

**Comment #6:** *"'However, tailored interventions of provider education combined with patient education (vs interventions without tailoring) led to larger reductions in low-value care use …' – this is a meta-regression result, so is prone to study-level confounding. Hence rather than saying 'led' (which is causal language) it is preferable to say is 'associated with'. Be cautious about these meta-regression results for this reason."*

RESPONSE

1) Thank you for the comment! We have revised the text accordingly.
2) The section now states: "*However, tailored interventions of provider education combined with patient education (vs interventions without tailoring) were associated with larger reductions in targeted low-value care use*…"
3) Page 15, line 419

**VERSION 3 – REVIEW**

| | |
|---|---|
| **REVIEWER NAME** | Riley, Richard |
| **REVIEWER AFFILIATION** | University of Birmingham, Institute of Applied Health Research |
| **REVIEWER CONFLICT OF INTEREST** | None |
| **AI DISCLOSURE** | No |
| **DATE REVIEW RETURNED** | 11-Jul-2025 |

| | |
|---|---|
| **GENERAL COMMENTS** | I thank the authors for addressing the comments I gave, and revising the article thoroughly. The results are now more complete and clear, and the conclusions better aligned with the evidence, with limitations noted, in particular the need for a network MA.<br><br>My only remaining comment is for the authors to interpret the heterogeneity results in the Results section. They do not discuss heterogeneity at all, although it is good to see they do present the prediction intervals and tau2 estimates now. The summary results tell us the best estimate of what happens on average, but the prediction intervals disseminate the range of potential true effects across settings; these are often quite wide so it is important to note more clearly that the heterogeneity may lead to the effect varying across settings (i.e. the summary effect is not the complete picture). The heterogeneity is briefly mentioned in the Discussion as a limitation ('the heterogeneity of the study results was high, likely at least partly explained by the variety of interventions and study contexts), but should be disseminated better in the results section too. |

| | |
|---|---|
| | Table 1: the column ‘estimated relative risk change’ … I think the authors mean ‘risk difference’ here? Or is this a % reduction in the risk? Relative risk change is odd wording, as I don’t think it is a change in the relative risk, but the actual risk? Please explain the calculation in a footnote below the table.<br><br>I look forward to this being published in BMJ Medicine. |

**VERSION 3 – AUTHOR RESPONSE**

Reviewer: 3
Prof. Richard Riley, University of Birmingham
Comments to the Author

**Comment #1:** *“My only remaining comment is for the authors to interpret the heterogeneity results in the Results section. They do not discuss heterogeneity at all, although it is good to see they do present the prediction intervals and tau2 estimates now. The summary results tell us the best estimate of what happens on average, but the prediction intervals disseminate the range of potential true effects across settings; these are often quite wide so it is important to note more clearly that the heterogeneity may lead to the effect varying across settings (i.e. the summary effect is not the complete picture). The heterogeneity is briefly mentioned in the Discussion as a limitation (‘the heterogeneity of the study results was high, likely at least partly explained by the variety of interventions and study contexts), but should be disseminated better in the results section too.”*

RESPONSE

7) Thank you for the comment. We have revised the text accordingly.
8) We added the following paragraph to the results section: “The heterogeneity of results was moderate or high in all intervention categories except one category (patient education combined with provider education and decision support). Prediction intervals were wide, including the null effect in all intervention categories, which suggests that the impact likely varies across different settings and specific interventions.“
9) page 14, lines 385-388

**Comment #2:** *“‘Table 1: the column ‘estimated relative risk change’ … I think the authors mean ‘risk difference’ here? Or is this a % reduction in the risk? Relative risk change is odd wording, as I don’t think it is a change in the relative risk, but the actual risk? Please explain the calculation in a footnote below the table.”*

RESPONSE

7) Thank you for the comment! We have revised the text as suggested.
8) We changed the column title from “*Estimated relative risk change”* to *“Estimated relative change”.* Furthermore, we added further details of the calculation to the footnote: * We estimated the relative change in two steps. First, we calculated the relative risk (RR) using the median control risk of having low-value care at follow-up within each intervention category. Second, we calculated relative change as (1 − RR) × 100.

9) Table 1
